# Observation of Josephson harmonics in tunnel junctions

**DOI:** 10.1038/s41567-024-02400-8

**Published:** 2024-02-14

**Authors:** Dennis Willsch, Dennis Rieger, Patrick Winkel, Madita Willsch, Christian Dickel, Jonas Krause, Yoichi Ando, Raphaël Lescanne, Zaki Leghtas, Nicholas T. Bronn, Pratiti Deb, Olivia Lanes, Zlatko K. Minev, Benedikt Dennig, Simon Geisert, Simon Günzler, Sören Ihssen, Patrick Paluch, Thomas Reisinger, Roudy Hanna, Jin Hee Bae, Peter Schüffelgen, Detlev Grützmacher, Luiza Buimaga-Iarinca, Cristian Morari, Wolfgang Wernsdorfer, David P. DiVincenzo, Kristel Michielsen, Gianluigi Catelani, Ioan M. Pop

**Affiliations:** 1 Jülich Supercomputing Centre, Forschungszentrum Jülich, Jülich, Germany; 2 IQMT, Karlsruhe Institute of Technology, Eggenstein-Leopoldshafen, Germany; 3 PHI, Karlsruhe Institute of Technology, Karlsruhe, Germany; 4 Departments of Applied Physics and Physics, Yale University, New Haven, CT USA; 5 Yale Quantum Institute, Yale University, New Haven, CT USA; 6 AIDAS, Jülich, Germany; 7 Physics Institute II, University of Cologne, Köln, Germany; 8 LPENS, Mines Paris-PSL, ENS-PSL, Inria, Université PSL, CNRS, Paris, France; 9 Alice & Bob, Paris, France; 10 IBM Quantum, IBM T. J. Watson Research Center, Yorktown Heights, NY USA; 11 PGI-9, Forschungszentrum Jülich and JARA Jülich-Aachen Research Alliance, Jülich, Germany; 12 RWTH Aachen University, Aachen, Germany; 13 CETATEA, INCDTIM, Cluj-Napoca, Romania; 14 PGI-2, Forschungszentrum Jülich, Jülich, Germany; 15 PGI-11, Forschungszentrum Jülich, Jülich, Germany; 16 Quantum Research Center, Technology Innovation Institute, Abu Dhabi, UAE

**Keywords:** Qubits, Superconducting properties and materials, Superconducting properties and materials

## Abstract

Approaches to developing large-scale superconducting quantum processors must cope with the numerous microscopic degrees of freedom that are ubiquitous in solid-state devices. State-of-the-art superconducting qubits employ aluminium oxide (AlO_*x*_) tunnel Josephson junctions as the sources of nonlinearity necessary to perform quantum operations. Analyses of these junctions typically assume an idealized, purely sinusoidal current–phase relation. However, this relation is expected to hold only in the limit of vanishingly low-transparency channels in the AlO_*x*_ barrier. Here we show that the standard current–phase relation fails to accurately describe the energy spectra of transmon artificial atoms across various samples and laboratories. Instead, a mesoscopic model of tunnelling through an inhomogeneous AlO_*x*_ barrier predicts percent-level contributions from higher Josephson harmonics. By including these in the transmon Hamiltonian, we obtain orders of magnitude better agreement between the computed and measured energy spectra. The presence and impact of Josephson harmonics has important implications for developing AlO_*x*_-based quantum technologies including quantum computers and parametric amplifiers. As an example, we show that engineered Josephson harmonics can reduce the charge dispersion and associated errors in transmon qubits by an order of magnitude while preserving their anharmonicity.

## Abstract (web summary)

The standard current–phase relation in tunnel Josephson junctions involves a single sinusoidal term, but real junctions are more complicated. The effects of higher Josephson harmonics have now been identified in superconducting qubit devices.

## Main

The Josephson effect^1,2^ is the keystone of quantum information processing with superconducting hardware: it constitutes a unique source of low-loss nonlinearity, which is essential for the implementation of superconducting quantum bits, and it plays a similarly fundamental role as the nonlinear current–voltage relation of diodes in semiconductor circuitry. In particular, tunnel Josephson junctions (JJs), formed by two overlapping superconducting films separated by a thin insulating barrier, have enabled superconducting hardware to become one of the leading platforms for the realization of fault-tolerant quantum computers^3–6^. JJs are also at the heart of quantum limited amplification^7^, metrological applications^8^ such as the definition of the voltage^9^ and a possible future current standard^10^, and they enable quantum detectors such as the microwave photon counter^11^. With the advancement^12–14^ of superconducting artificial atom technology, the measurement and understanding of subtle features in the Josephson effect, similar to the fine structure discovered in natural atoms, is increasingly relevant in setting the accuracy of both circuit control and circuit models.

**Fig. 1:**
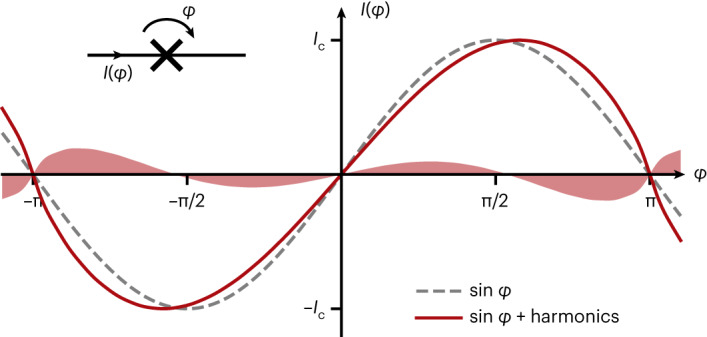
Josephson harmonics are relevant for the C*φ*R of tunnel junctions. The nonlinear C*φ*R is the fingerprint of a JJ, which relates the supercurrent *I*(*φ*) to the phase *φ* (inset). For tunnel JJs, the C*φ*R has been considered to be purely sinusoidal (dashed grey line; equation (1)), with the maximum given by the critical current *I*_c_. However, as we show in this work, even in tunnel JJs, the underlying microscopic complexity of the charge transport can manifest in the contribution of higher harmonics to the C*φ*R. As an example, the red line shows a C*φ*R consistent with measured data (CD1 of the KIT sample), which includes the harmonics expected from a mesoscopic model assuming an inhomogeneous AlO_*x*_ barrier. The shaded red area shows the difference from the purely sinusoidal C*φ*R. We provide C*φ*Rs for all other measured samples in Supplementary Fig. 7.

Although the mesoscopic dimensions of JJs imply the existence of many conduction channels, for tunnel junctions this complexity is usually condensed into a single effective parameter, the critical current *I*_c_, in the well-known Josephson current–phase relation, C*φ*R (grey line in Fig. 1):

1 $$I(\varphi )={I}_{{{{\rm{c}}}}}\sin \varphi \,,$$

where *φ* is the superconducting phase difference across the junction. This simplification is remarkable given the fact that other types of junctions, such as weak links, point contacts and ferromagnetic JJs, generally exhibit non-sinusoidal C*φ*Rs containing higher Josephson harmonics: $\sin (2\varphi )$, $\sin (3\varphi )$ and so on^15–21^. Here we show that Josephson harmonics are also relevant for tunnel JJs (Fig. 1).

**Fig. 2:**
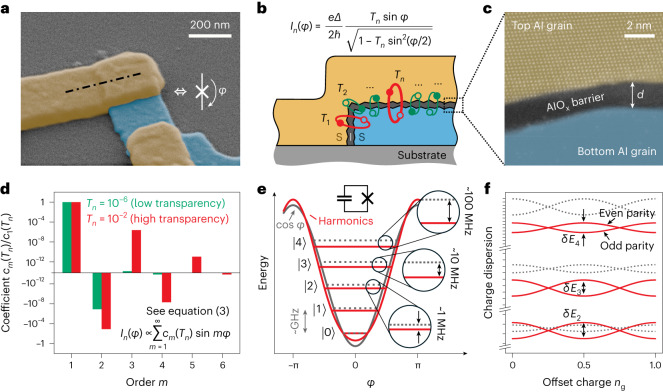
Josephson harmonics result from junction barrier inhomogeneity. **a**, False-coloured scanning electron microscope image of a typical Al–AlO_*x*_–Al JJ fabricated at KIT. The bottom and top electrodes are coloured blue and yellow, respectively. Inset, circuit symbol for a JJ with phase difference *φ* across the barrier. **b**, Cross-section schematic of the superconductor–insulator–superconductor JJ at the location indicated by the dash-dotted line in **a**. The supercurrent *I*_*n*_(*φ*) of each conduction channel *n* = 1, …, *N* depends on its transparency *T*_*n*_ (equation (2)). We sketch a distribution of multiple low and a few high transparencies *T*_1_, …, *T*_*N*_ in green and red, respectively. **c**, False-coloured high-angle annular dark field STEM image centred on the AlO_*x*_ tunnel barrier of a typical JJ fabricated at KIT, with average thickness *d* ≈ 2 nm as indicated by the white arrow. Individual columns of atoms of the Al grain in the top electrode are visible due to zone axis alignment, which is not the case for the bottom Al electrode (additional STEM images with thickness variations and structural defects such as grain boundaries are shown in Supplementary Fig. 27). **d**, Normalized Fourier coefficients *c*_*m*_(*T*_*n*_) of the JJ C*φ*R (equation (2)) for a low (10^−6^, green) and high (10^−2^, red) transparency channel. Note the alternating sign for even and odd order *m* and the fact that high-transparency channel coefficients (in red) remain relevant to higher order. **e**, Sketch of how the higher-order terms in the JJ Hamiltonian modulate the potential and shift the energy levels (red) of superconducting artificial atoms compared to a purely cos*φ* potential (grey). In this Article, we focus on transmon devices, which consist of a large capacitor in parallel to the JJ (refer to the circuit schematic inset). The discrepancy between the models generally increases at higher levels. **f**, The higher-order Josephson harmonics also influence the charge dispersion of the transmon levels versus offset charge *n*_g_. The two branches per energy level correspond to a change between even and odd charge parity (that is, quasiparticle tunnelling^79,80^; Supplementary Fig. 23 in Supplementary Section IIIC).

To understand the limits of the approximation equation (1) for tunnel junctions, we have to take a closer look at commonly used Al–AlO_*x*_–Al JJs, fabricated by shadow evaporation^22^ and schematized in Fig. 2a–c, which reveals a complex microscopic reality. The C*φ*R of the junction is obtained by summing the supercurrents of *N* conduction channels, $I(\varphi )=\mathop{\sum }\nolimits_{n = 1}^{N}{I}_{n}(\varphi )$. Each channel (Fig. 2b) has a transparency-dependent C*φ*R (refs. ^16,23^) that can be expressed as a Fourier series:

2 $${I}_{n}(\varphi )\propto \frac{{T}_{n}\sin \varphi }{\sqrt{1-{T}_{n}{\sin }^{2}(\varphi /2)}}=\,\hspace{2.22144pt}\mathop{\sum }\limits_{m=1}^{\infty }\,\hspace{2.22144pt}{c}_{m}({T}_{n})\sin (m\varphi )\,.$$

The conduction channel transparency *T*_*n*_ is defined as the tunnel probability for an electron impinging on the insulating barrier of channel *n*, and *c*_*m*_(*T*_*n*_) are the order *m* Fourier coefficients for *I*_*n*_(*φ*). These coefficients alternate in sign and decay in magnitude with increasing order *m* (Fig. 2d). The ratio ∣*c*_*m*+1_/*c*_*m*_∣ of successive coefficients increases with *T*_*n*_ (Supplementary Section IA): the more transparent a channel, the more relevant the contribution of higher harmonics. To put it simply, in higher-transparency channels, it is more likely for Cooper pairs to tunnel together in groups of *m*, which correspond to the $\sin (m\varphi )$ terms in the C*φ*R.

In the limit *T*_*n*_ → 0, only the $\sin \varphi$ term of equation (2) survives. If all channels in a JJ are in this limit, we recover the purely sinusoidal C*φ*R of equation (1), with the critical current of the junction *I*_c_ proportional to the sum of transparencies. Assuming a perfectly homogeneous barrier, for a typical junction with ~μm^2^ area and resistance comparable to the resistance quantum, one expects *N* ≈ 10^6^ and *T*_*n*_ ≈ 10^−6^ (refs. ^24,25^), leading to negligible (below 10^−6^) corrections to the purely sinusoidal C*φ*R.

But is this the reality? Here we argue that in the presence of contaminants, atomic scale defects^26^ and random crystalline orientations of the grains in contact, evidenced by scanning transmission electron microscope (STEM) images and molecular dynamics simulations (Fig. 2c and Supplementary Section IV), we have reasons to doubt it. In fact, about two decades ago, AlO_*x*_ barrier inhomogeneity motivated the transition in magnetic junctions to more uniform oxides such as MgO (refs. ^27–29^). Consequently, we expect a distribution of transparencies in AlO_*x*_ (refs. ^30,31^) with possibly a few relatively high-transparency channels^32,33^ introducing measurable corrections to the C*φ*R (Fig. 1). The microscopic structure of each barrier is therefore imprinted on the C*φ*R of the JJ, and the challenge is how to experimentally access this information.

For our study of tunnel JJs, we use transmon devices^34^, in which a JJ is only shunted by a large capacitor to form a nonlinear oscillator with the potential energy defined by the C*φ*R of the junction (Fig. 2e). The resulting individually addressable transition frequencies in the microwave regime can be measured using circuit quantum electrodynamics techniques^35^. We compare the spectra of multiple samples to the prediction of the standard transmon Hamiltonian based on a sinusoidal C*φ*R (equation (1)) and find increasing deviations for the higher energy levels of all samples, as sketched in Fig. 2e,f. Only by accounting for higher harmonics in the C*φ*R are we able to accurately describe the entire energy spectrum. A similar methodology was used in ref. ^18^ to reconstruct the C*φ*R of a semiconductor nanowire Josephson element. While our study focuses on transmon qubits, the conclusions we draw regarding the C*φ*R of tunnel junctions should trigger a re-evaluation of the current models for tunnel-JJ-based devices used in quantum technology and metrology^35–39^.

Since transmons are widely available in the community, we are able to measure and model the spectra of multiple samples from laboratories around the globe: fixed-frequency transmons fabricated and measured at the Karlsruhe Institute of Technology (KIT; Supplementary Fig. 18) in three cooldowns (CDs; Supplementary Fig. 19) and Ecole Normale Supérieure (ENS) Paris (same device as in ref. ^40^), a tunable transmon subject to an in-plane magnetic field at the University of Cologne (Köln; identical setup and similar device as in ref. ^41^; Supplementary Fig. 23) and 20 qubits from the IBM Hanoi processor (IBM). All transmons are based on standard Al–AlO_*x*_–Al tunnel junctions (Fig. 2) and are measured in either a three-dimensional architecture or a two-dimensional coplanar waveguide geometry (for detailed descriptions of each sample, see Supplementary Section III). The spectroscopy data consists of (1) transition frequencies *f*_0*j*_ into transmon states *j* = 1, 2, … up to *j* = 6, each measured as *j*-photon transitions at frequencies *f*_0*j*_/*j*, and (2) the resonator frequencies ${f}_{{{{\rm{res}}}},\;j}$ depending on the transmon state *j* = 0, 1 (Methods).

**Fig. 3:**
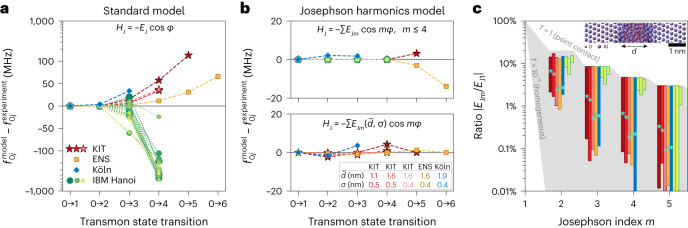
Standard transmon model fails to describe the measured frequency spectra. **a**, Differences between the frequencies ${f}_{0j}^{{{\,{\rm{model}}}}}$ predicted by the standard transmon model in equation (3) and the measured transitions ${f}_{0j}^{{{\,{\rm{experiment}}}}}$. The markers indicate the different experiments at KIT (red stars), ENS (yellow squares), Köln (blue diamonds) and IBM (green circles). For the KIT experiment, we show results for three successive CDs of the same sample (CD1–3, dark red to bright red, respectively). For the Köln experiment, we chose a set of measured transitions at a fixed magnetic field (blue arrow in Fig. 4a). For the IBM experiment, we show results for 20 qubits in the IBM Hanoi device, using different marker sizes and shades of green. Measurement imprecisions are on the order of 1 MHz and not visible in the figure. Note that the scale on the vertical axis is linear between ±100 MHz and logarithmic onward. Dashed and dotted lines are guides to the eye. **b**, Same as **a**, with ${f}_{0j}^{{{\,{\rm{model}}}}}$ given by the Josephson harmonics Hamiltonian in equation (4). Top, model truncated at *E*_J4_. Bottom, mesoscopic model of tunnelling through an inhomogeneous AlO_*x*_ barrier, where ${E}_{{{{\rm{J}}}}m}(\bar{d},\sigma )$ is parameterized in terms of the average barrier thickness $\bar{d}$ and the standard deviation *σ* (equation (5); the fit values are listed in the table inset). **c**, Ranges of the Josephson harmonics ratios ∣*E*_J*m*_/*E*_J1_∣ that are consistent with the measured spectra. The ranges are represented by coloured vertical bars using the same colouring as in **a**. For the IBM Hanoi device, we show the ranges for qubits 0–2 from left to right (ranges for the other qubits are shown in Supplementary Section IC3). The shaded grey area highlights the region between two limiting cases: the fully open quantum point contact with unit transparency and a homogeneous barrier with *T*_*n*_ = 10^−6^ for all *n*. Turquoise markers on the vertical bars indicate the harmonics ratios calculated from the mesoscopic model, where the average thickness $\bar{d}$ and the standard deviation *σ* are given in **b**. Inset, an Al–AlO_*x*_–Al junction obtained from molecular dynamics simulations (Supplementary Fig. 25) with average barrier thickness $\bar{d}=1.5\,{{{\rm{nm}}}}$ (Fig. 2c).

In Fig. 3, we compare the measured transition frequencies to predictions ${f}_{0j}^{{{\;{\rm{model}}}}}$, obtained by exact diagonalization of two different model Hamiltonians. The first model is the standard transmon model, which has served the community for over 15 years^34^

3 $${H}_{{{{\rm{std}}}}}=4{E}_{{{{\rm{C}}}}}{(n-{n}_{{{{\rm{g}}}}})}^{2}-{E}_{{{{\rm{J}}}}}\cos \varphi +{H}_{{{{\rm{res}}}}}\,,$$

where *E*_C_ is the charging energy, *E*_J_ is the Josephson energy, *n*_g_ is the offset charge and the operators *n* and *φ* represent the charge normalized by twice the electron charge and the phase difference across the junction, respectively. All models include the readout resonator Hamiltonian given by ${H}_{{{{\rm{res}}}}}={{\varOmega }}{a}^{{\dagger} }a+Gn(a+{a}^{{\dagger} })$, where *Ω* is the bare resonator frequency, *G* is the electrostatic coupling strength and *a*^†^ (*a*) is the bosonic creation (annihilation) operator. Including ${H}_{{{{\rm{res}}}}}$ ensures that dressing of the states due to transmon-resonator hybridization is taken into account^34,35,42,43^.

We obtain the parameter set (*E*_C_, *E*_J_, *Ω*, *G*) of the standard transmon model in equation (3) by solving the inverse eigenvalue problem (IEP)^44–47^ for the measured spectroscopy data (Methods). For the Köln sample, these data include the offset charge dispersion (additional data for different magnetic fields are given in Supplementary Section IID). We note that the IEP is the very same science problem that was historically solved to model the energy spectra of natural atoms and molecules (see for example refs. ^48–50^), which led to the discovery of the fine structure.

In Fig. 3a, we show that the standard transmon model in equation (3) fails to describe the measured frequency spectra for all samples. The observed deviations are much larger than the measurement imprecision, for which we can set a conservative upper bound on the order of 1 MHz. While the standard transmon model with two parameters can trivially match the *f*_01_ and *f*_02_ transitions, the measured *f*_03_ can already deviate by more than 10 MHz. The deviations are positive for the KIT, ENS and Köln samples, while the IBM transmons mostly show negative deviations (Supplementary Section IC5). It is important to remark that other corrections, such as the stray inductance in the JJ leads, hidden modes coupled to the qubit, the coupling between qubits as present on the IBM multi-qubit device, or an asymmetry in the superconducting energy gaps, while being relevant, cannot fully account for the measured discrepancy (Supplementary Section ID). Notably, similar deviations can be found in previously published transmon spectra^41,51–53^, as we detail in Supplementary Fig. 4 and Supplementary Sections IC2 and IC4.

In Fig. 3b, we demonstrate that orders of magnitude better agreement with our measured spectra can be achieved by using the Josephson harmonics model:

4 $${H}_{{{{\rm{har}}}}}=4{E}_{{{{\rm{C}}}}}{(n-{n}_{{{{\rm{g}}}}})}^{2}-\mathop{\sum}\limits_{m\ge 1}{E}_{{{{\rm{J}}}}m}\cos (m\varphi )+{H}_{{{{\rm{res}}}}}\,.$$

In general, the values *E*_J*m*_ are a fingerprint of each junction’s channel-transparency distribution *ρ*(*T*) with many degrees of freedom. Here we consider two simplified models (further models are discussed in Supplementary Section IC): (1) a phenomenological model truncated at *E*_J4_ (top panel) and (2) a mesoscopic model of tunnelling through a non-uniform oxide barrier (bottom panel). We note that the phenomenological *E*_J4_ model guarantees agreement for the lowest four transitions (Methods), and while many samples have physically reasonable *E*_J*m*_ coefficients when truncating at *E*_J4_, a few JJs require terms up to *E*_J6_ (Supplementary Section IC3).

The mesoscopic model allows us to derive $\rho (T;\bar{d},\sigma )$ based on a Gaussian thickness distribution with average thickness $\bar{d}$ and standard deviation *σ* (Supplementary Section IB4). As a consequence, all Josephson harmonics for *m* ≥ 2 are parameterized in terms of the two parameters $\bar{d}$ and *σ* according to

5 $${E}_{{{{\rm{J}}}}m}(\bar{d},\sigma )\propto \int\nolimits_{0}^{1}\,{c}_{m}(T)\,\rho (T;\bar{d},\sigma )\,{{{\rm{d}}}}T\,,$$

where the Fourier coefficients *c*_*m*_(*T*) (equation (2) and Fig. 2d) are weighted by the channel-transparency distribution $\rho (T;\bar{d},\sigma )$. In this model, relatively large ratios ∣*E*_J*m*_/*E*_J1_∣ originate from higher-transparency contributions from the narrower regions of the barrier (compare the STEM images in Supplementary Fig. 27). The model can describe the samples at KIT, ENS and Köln (Fig. 3b) but not the IBM device (Supplementary Section IB4). The model parameters $\bar{d}$ and *σ* (Fig. 3b) are comparable to results from molecular dynamics simulation and STEM pictures of the oxide barrier (Supplementary Section IV).

In Fig. 3c, we indicate the ranges of *E*_J*m*_ coefficients consistent with the measured spectra. The bars represent the lower and upper limits of Josephson harmonics ratios ∣*E*_J*m*_/*E*_J1_∣. The corresponding $\sin (m\varphi )$ contribution to the C*φ*R is given by *m*∣*E*_J*m*_/*E*_J1_∣ (see Fig. 1 for the KIT sample). The ratios lie between two limiting cases spanning the physical regime (shaded grey area): (1) the upper limit, ∣*E*_J*m*_/*E*_J1_∣ = 3/(4*m*^2^ − 1), corresponds to an open quantum point contact—that is, one channel with *T* = 1—and (2) the lower limit, ∣*E*_J*m*_/*E*_J1_∣ ≈ (*T*/4)^*m*−1^/*m*^3/2^, corresponds to a perfectly homogeneous low-transparency barrier (*T*_*n*_ = *T* = 10^−6^ for all *n*). For the scanning routine, we include harmonics up to *E*_J10_ to obtain results within the physical regime and to see when truncation is possible (Methods). Remarkably, for all samples, the *E*_J2_ contribution is in the few percent range even after considering additional corrections such as series inductance or gap asymmetry in the superconducting electrodes (Supplementary Section ID).

The Josephson harmonics ratios computed from the mesoscopic model in equation (5) are shown with turquoise markers. Notice that the barrier evolved between CDs of the KIT sample due to ageing (CD1 to CD2) and thermal annealing (CD2 to CD3) (Supplementary Section IIIA). Even for the most homogeneous barrier (CD3), the second-harmonic contribution is *E*_J2_/*E*_J1_ ≈ −2.4%, implying that there would be at least one conduction channel with a transparency *T* ≥ 0.29 (Supplementary Section IA). The methodology presented in Fig. 3 can serve as a tool to characterize Josephson harmonics and tunnel barrier homogeneity, independent of circuit design.

**Fig. 4:**
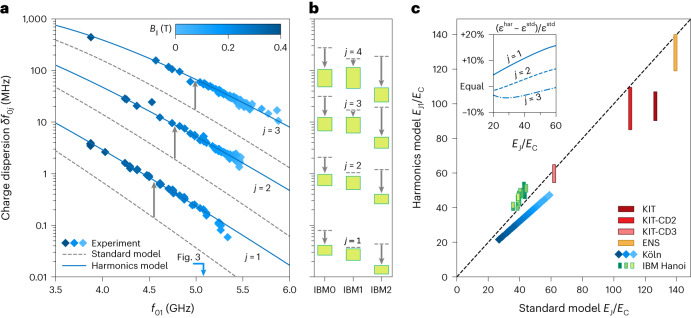
Influence of Josephson harmonics on the charge dispersion. **a**, Measured charge dispersion *δf*_0*j*_ (blue diamonds) of states *j* = 1, 2, 3 for the experiment in Köln, plotted as a function of the *f*_01_ frequency. All transition frequencies are tuned, as the Josephson energy is suppressed by up to 35% by means of an in-plane magnetic field *B*_∥_ swept to 0.4 T. The standard model in equation (3), shown in dashed grey lines, underestimates the charge dispersion by a factor of 2–7 (grey arrows), while the Josephson harmonics model in equation (4) plotted in solid blue overlaps the measured data. Note that both are computed with the same parameters used for Fig. 3; the Josephson energy is reduced with increasing magnetic field, and the other parameters such as the *E*_J*m*_/*E*_J1_ ratios are kept constant. The blue arrow indicates *f*_01_ = 5.079 GHz, corresponding to the dataset shown in Fig. 3. **b**, Evidence that Josephson harmonics can reduce the charge dispersion by an order of magnitude (grey arrows). The dashed grey lines represent the standard model predictions. In contrast, the green bars show results from all Josephson harmonics models. The data correspond to IBM qubits 0–2 (green bars in Fig. 3c) for the levels *j* = 1, 2, 3, 4; results for all other samples are shown in Supplementary Fig. 6. **c**, The values of *E*_J1_/*E*_C_ change compared to the standard model *E*_J_/*E*_C_, which constitutes the main correction to the predicted charge dispersions in **a** and **b**. The bars represent the range of suitable ratios *E*_J1_/*E*_C_ (Fig. 3c) for the successive CDs of the KIT sample (red bars), the ENS sample (yellow bar), the Köln sample (blue diamonds, using the same colour coding as in s**)** and the IBM Hanoi device (green bars). The dashed diagonal line indicates the case in which the ratios *E*_J1_/*E*_C_ of the harmonics model and *E*_J_/*E*_C_ of the standard model are equal. Inset, correction (*ε*^har^ − *ε*^std^)/*ε*^std^ to the relative charge dispersion *ε* = *δf*_0*j*_/*f*_01_ for fixed ${E}_{{{{\rm{J}}}}}^{{{{\rm{std}}}}}/{E}_{{{{\rm{C}}}}}^{{{{\rm{std}}}}}={E}_{{{{\rm{J}}}}1}^{{{{\rm{har}}}}}/{E}_{{{{\rm{C}}}}}^{{{{\rm{har}}}}}$ for the Köln sample, where *ε*^std^ is given by the standard charge dispersion^34^ and *ε*^har^ is computed using the Josephson harmonics model.

Since the charge dispersion increases for higher transmon levels (even for the standard transmon Hamiltonian^34^; Fig. 2f) and is exponentially sensitive to the shape of the JJ potential (Fig. 2e), a natural question arises: what are the consequences of the Josephson harmonics on the transmon’s susceptibility to offset charges? In Fig. 4a, we show the measured charge dispersion *δf*_0*j*_ of the Köln device for states *j* = 1, 2, 3 versus the first transition frequency *f*_01_, which is tuned by an in-plane magnetic field *B*_∥_ of up to 0.4 T (see Supplementary Section IIIC for details). The charge dispersion predicted by the standard model (dashed grey lines) falls short of the measurements by a factor of 2–7 for the three measured transitions. In contrast, when using the Josephson harmonics model, the computed charge dispersion matches the data (blue lines). We emphasize that for both models, we use the same parameters as in the Fig. 3 analysis (that is, the standard model and the *E*_J4_ model) and vary the first Josephson energy to match the qubit frequency *f*_01_ for different magnetic fields while keeping the *E*_J*m*_/*E*_J1_ ratios constant.

Interestingly, the presence of large Josephson harmonics, as in the case of the IBM qubits (Fig. 3c), can also reduce the charge dispersion, which directly decreases charge noise decoherence. We show evidence for this in Fig. 4b, on the first three IBM qubits, for which the charge dispersion of the qubit transition can be a factor of 4 lower than expected from the standard transmon model. This observation indicates a possible optimization route in which Josephson harmonics are engineered (for example, by shaping the channel transparencies or adding inductive elements in series) and the spectrum is steered towards regions of reduced charge dispersion and increased anharmonicity (Supplementary Fig. 8). A recent work^54^ proposes a similar approach to engineer arbitrary-shaped C*φ*Rs using networks of effective high-transparency JJs, each of which is a series of tunnel JJs.

The main reason for the failure of the standard transmon model in describing the charge dispersion (when fitted to *f*_01_ and *f*_02_) is that it misjudges the value of *E*_J_/*E*_C_. To quantify this effect, in Fig. 4c we plot the values of *E*_J1_/*E*_C_ from the Josephson harmonics model against the value of *E*_J_/*E*_C_ from the standard model. Indeed, the *E*_J1_/*E*_C_ ranges for many of our measurements are not compatible with the standard model *E*_J_/*E*_C_ ratio (dashed diagonal). We note that when evaluated for the same *E*_J_/*E*_C_, the Josephson harmonics correction to the charge dispersion is relatively small (inset of Fig. 4c).

In summary, we have shown that for ubiquitous AlO_*x*_ tunnel junctions, the microscopic structure, currently underappreciated in its complexity, causes level shifts and modifies the charge dispersion in superconducting artificial atoms. In order to fully describe the measured transmon energy spectra, we amend the standard $\sin \varphi$ Josephson C*φ*R for tunnel junctions to include higher-order $\sin (m\varphi )$ harmonics, with the relative amplitude of the *m* = 2 term in the few percent range. We confirm this finding in various sample geometries from four different laboratories, and we argue that the source of the Josephson harmonics is the presence of relatively higher-transparency channels with *T* ≫ 10^−6^ in the AlO_x_ tunnel barrier. The methodology shown here can reveal percent-level deviations from a sinusoidal C*φ*R, which are hard to detect in more standard measurements based on asymmetric direct current superconducting quantum interference devices^55^.

The observation of Josephson harmonics in tunnel junctions highlights the need to revisit established models for superconducting circuits. Our work directly impacts the design and measurement of transmon qubits and processors: As an illustration, we show that by engineering Josephson harmonics, the dephasing due to charge noise can be reduced by an order of magnitude without sacrificing anharmonicity. These results ask for future research studying the implications of Josephson harmonics and associated Andreev bound states in other tunnel-JJ-based circuits, for example fluxonium or generalized flux qubits^56^.

In general, we expect the inclusion of the harmonics will refine the understanding of superconducting artificial atoms and will directly benefit, among others, quantum gate and computation schemes relying on higher levels^57–63^, quantum-non-demolition readout fidelities^64–66^ and frequency crowding mitigation in quantum processors^67^. Josephson harmonics will probably also have to be accounted for in topological JJ circuits^68–70^, parametric pumping schemes employed in microwave amplifiers and bosonic codes^71,72^, amplification and mixing^7,73,74^, JJ metrological devices^8–10^, Floquet qubits^75,76^, protected Josephson qubits^68,70,77^ and so on, and they can be harnessed to realize Josephson diodes^78^. As devices become increasingly sophisticated with progressively smaller error margins, higher-order Josephson harmonics will need to be either suppressed via the development of highly uniform and low-transparency barriers or engineered and included as an integral part of the device physics.

## Methods

### Diagonalizing the Hamiltonians to obtain model predictions

We construct the matrices of *H*_std_ in equation (3) and *H*_har_ in equation (4) by first diagonalizing the bare transmon matrix (excluding ${H}_{{{{\rm{res}}}}}$) in the charge basis $\{\left\vert n\right\rangle \}$, where $4{E}_{{{{\rm{C}}}}}{(n-{n}_{{{{\rm{g}}}}})}^{2}={\sum }_{n}4{E}_{{{{\rm{C}}}}}{(n-{n}_{{{{\rm{g}}}}})}^{2}\left\vert n\right\rangle \,\left\langle n\right\vert$ is diagonal and $-{E}_{{{{\rm{J}}}}m}\cos (m\varphi )=-{\sum }_{n}{E}_{{{{\rm{J}}}}m}/2\,(\left\vert n\right\rangle \,\left\langle n+m\right\vert +\left\vert n+m\right\rangle \,\left\langle n\right\vert )$ has constant entries −*E*_J*m*_/2 on the *m*th subdiagonal (we ensure enough terms by generally verifying that the predictions do not change if more terms are included). This yields the transmon eigenenergies *E*_*j*_ and eigenstates $\left\vert\, j\right\rangle$. Then we diagonalize the joint transmon-resonator Hamiltonian ${H}_{{{{\rm{std}}}}/{{{\rm{har}}}}}={\sum }_{j}{E}_{j}\left\vert\, j\right\rangle \,\left\langle\, j\right\vert +{{\varOmega }}{a}^{{\dagger} }a+{\sum }_{j,{j}^{{\prime} }}G\left\vert\, j\right\rangle \,\left\langle\, j\right\vert \,n\,\left\vert\, {j}^{{\prime} }\right\rangle \,\left\langle\, {j}^{{\prime} }\right\vert (a+{a}^{{\dagger} })$, where $a={\sum }_{k}\sqrt{k+1}\left\vert k\right\rangle \,\left\langle k+1\right\vert$. To each resulting eigenenergy ${E}_{\overline{l}}$ and eigenstate $\left\vert \overline{l}\,\right\rangle$, we assign a photon label *k* and a transmon label *j* based on the largest overlap $\mathrm{max}_{k,\;j}| \left\langle kj| \overline{l}\,\right\rangle |$ (this only works for small *k*; Supplementary Section IIC), which yields the dressed energies ${E}_{\overline{kj}}$ and states $\left\vert \overline{kj}\,\right\rangle$. This procedure is done for both *n*_g_ = 0 and *n*_g_ = 1/2. From the resulting dressed energies ${E}_{\overline{kj}}({n}_{{{{\rm{g}}}}})$, we compute the transmon transition frequencies ${f}_{0j}^{{{\;{\rm{model}}}}}({n}_{{{{\rm{g}}}}})=({E}_{\overline{0j}}({n}_{{{{\rm{g}}}}})-{E}_{\overline{00}}({n}_{{{{\rm{g}}}}}))/2\uppi$ and the resonator frequencies ${f}_{{{{\rm{res}}}},\,j}^{{{\,{\rm{model}}}}}({n}_{{{{\rm{g}}}}})=({E}_{\overline{1j}}({n}_{{{{\rm{g}}}}})-{E}_{\overline{0j}}({n}_{{{{\rm{g}}}}}))/2\uppi$ (setting *ℏ* = 1). The predicted frequencies are then given by ${f}_{0j}^{{{\,{\rm{model}}}}}=({f}_{0j}^{{{\,{\rm{model}}}}}(0)+{f}_{0j}^{{{\,{\rm{model}}}}}(1/2))/2$, ${f}_{{{{\rm{res}}}},j}^{{{\,{\rm{model}}}}}=({f}_{{{{\rm{res}}}},j}^{{{\,{\rm{model}}}}}(0)+{f}_{{{{\rm{res}}}},j}^{{{\,{\rm{model}}}}}(1/2))/2$, and the charge dispersion is $\delta {f}_{0j}^{{{\,{\rm{model}}}}}=|\, {f}_{0j}^{{{\,{\rm{model}}}}}(0)-{f}_{0j}^{{{\,{\rm{model}}}}}(1/2)|$. We consistently use *n* = −*N*, …, *N* with *N* = 14 and thus 2*N* + 1 = 29 charge states, *j* = 0, …, *M* − 1 with *M* = 12 transmon states and *k* = 0, …, *K* − 1 with *K* = 9 resonator states, where *N*, *M* and *K* have been chosen by verifying that the model predictions change by less than a few kHz when adding more states.

### Solving the IEP to obtain model parameters

The inverse problem^47,81^ to obtain the parameters **x**^std^ of the standard model Hamiltonian in equation (3) and **x**^har^ of the harmonics model Hamiltonian in equation (4), such that the linear combinations of eigenvalues ${{{\bf{f}}}}=(\,{f}_{01}^{{{\,{\rm{model}}}}},{f}_{02}^{{{\,{\rm{model}}}}},\ldots ,{f}_{0{N}_{f}}^{{{\,{\rm{model}}}}},{f}_{{{{\rm{res}}}},0}^{{{\,{\rm{model}}}}},{f}_{{{{\rm{res}}}},1}^{{{\,{\rm{model}}}}})$ agree with the measured data, is an instance of the Hamiltonian parameterized IEP (HamPIEP; Supplementary Section IIA2). We solve the HamPIEP using the globally convergent Newton method^82^ with cubic line search and backtracking^83^ and the Broyden–Fletcher–Goldfarb–Shanno algorithm^84^ as implemented in TensorFlow Probability^85,86^. The Jacobian ∂**f**/∂**x** is obtained by performing automatic differentiation through the diagonalization with TensorFlow. For the *E*_J4_ model shown in Fig. 3b, the IEP is solved unambiguously for **x** = (*E*_J1_, *E*_J2_, *E*_J3_, *E*_J4_, *Ω*, *G*) using the lowest four transmon transition frequencies, and we fix the values ${E}_{{{{\rm{C}}}}}^{{{{\rm{KIT}}}}}/h=$ 242 MHz, ${E}_{{{{\rm{C}}}}}^{{{{\rm{ENS}}}}}/h=$ 180 MHz and ${E}_{{{{\rm{C}}}}}^{{{{\rm{IBM}}}}}/h=$ 300 MHz, respectively, to make the models consistent with further available information such as accurate finite-element simulations (Supplementary Section IIIA) or knowledge of the transmon capacitance. For the mesoscopic model (Supplementary Section IB4), the parameters ${{{\bf{x}}}}=(\bar{d},\sigma ,{E}_{{{{\rm{C}}}}},{E}_{{{{\rm{J}}}}},{{\varOmega }},G)$ are found by minimizing the function $\mathop{\sum }\nolimits_{j = 1}^{{N}_{f}}|\;{f}_{0j}^{{{\,{\rm{model}}}}}/j-{f}_{0j}^{{{\,{\rm{experiment}}}}}/j| +\mathop{\sum }\nolimits_{j = 0}^{1}|\, {f}_{{{{\rm{res}}}},\,j}^{{{\,{\rm{model}}}}}-{f}_{{{{\rm{res}}}},\,j}^{{{\,{\rm{experiment}}}}}|$ using the Broyden–Fletcher–Goldfarb–Shanno algorithm. The initial values for the minimization are given by $\bar{d}=1.64\,{{{\rm{nm}}}}$ (taken from the molecular dynamics result in Supplementary Section IV), $\sigma =\bar{d}/4$ (also Supplementary Table 2) and (*E*_C_, *E*_J_, *Ω*, *G*) from the standard transmon model. For the Köln data, where 288 data points have to be described by the same model parameters **x** (Fig. 4a) and only the Josephson energy is varied, we use cubic interpolation as a function of ${f}_{01}^{{{\,{\rm{model}}}}}$ and include only a few central points for the available frequencies in the solution of the IEP (the residuals are given in Supplementary Fig. 17). All model parameters are provided in the repository^87^ accompanying this manuscript.

### Scanning the Josephson energies

To obtain the range of suitable Josephson energies {*E*_J*m*_} (shown in Fig. 3c) that are consistent with a measured spectrum, we use an exhaustive scanning procedure. A spectroscopy dataset of *N*_*f*_ measured transition frequencies *f*_0*j*_, *j* = 1, …, *N*_*f*_ and two resonator frequencies ${f}_{{{{\rm{res}}}},0}$ and ${f}_{{{{\rm{res}}}},1}$ uniquely determines—via the HamPIEP—the values ${{{\bf{x}}}}=({E}_{{{{\rm{J1}}}}},\ldots ,{E}_{{{{\rm{J}}}}{N}_{f}},{{\varOmega }},G)$. We then scan the values of four additional ratios ${{{\bf{y}}}}=({E}_{{{{\rm{J}}}}{N}_{f}+1}/{E}_{{{{\rm{J}}}}1},\ldots ,{E}_{{{{\rm{J}}}}{N}_{f}+4}/{E}_{{{{\rm{J}}}}1})$ for each of these four *E*_J*m*_/*E*_J1_ over 16 geometrically spaced values between the point contact limit 3(−1)^*m*+1^/(4*m*^2^ − 1) and ${(-1)}^{m+1}\min \{1{0}^{-7},| {E}_{{{{\rm{J}}}}m+1}/{E}_{{{{\rm{J}}}}1}| \}$ (always skipping the first to ensure ∣*E*_J*m*_/*E*_J1_∣ > ∣*E*_J*m*+1_/*E*_J1_∣). Additionally, we include **y** = (0, 0, 0, 0) to see if truncation at ${E}_{{{{\rm{J}}}}{N}_{f}}$ is allowed. For each combination **y**, we solve the HamPIEP for the spectroscopy data to obtain the unique solution **x**. We call the ratios ${{{\bf{e}}}}=(1,{E}_{{{{\rm{J}}}}2}/{E}_{{{{\rm{J}}}}1},\ldots ,{E}_{{{{\rm{J}}}}{N}_{f}+4}/{E}_{{{{\rm{J}}}}1})$ a trajectory that can reproduce the spectrum. However, the trajectory **e** may not be physical, since (1) some of the leading ratios *E*_J*m*_/*E*_J_ for *m* ≤ *N*_*f*_ might be beyond the quantum point-contact limit, (2) the Josephson energies might not be strictly decreasing in absolute value for increasing order *m*, or (3) the signs might not be alternating. Note that this can also happen when the Josephson harmonics model in equation (4) is truncated at too-low orders (Supplementary Section IC3). For all *E*_J*m*_, the maximum and minimum possible ratios ∣*E*_J*m*_/*E*_J_∣ define the vertical bars in Fig. 3c.

## Online content

Any methods, additional references, Nature Portfolio reporting summaries, source data, extended data, supplementary information, acknowledgements, peer review information; details of author contributions and competing interests; and statements of data and code availability are available at 10.1038/s41567-024-02400-8.

### Supplementary information

Supplementary Information Supplementary Sections I–IV and Figs. 1–27.

## Data Availability

The spectroscopy data and the model parameters that support the findings of this study are available in the Jülich DATA repository at 10.26165/JUELICH-DATA/LGRHUH.
