# Supplementary material for: Observation of Josephson harmonics in tunnel junctions
Source: Nat Phys. 2024 Feb 14;20(5):815–21. doi: 10.1038/s41567-024-02400-8 (PMC11116114; doi:10.1038/s41567-024-02400-8)
Supplement: Supplementary file 1 — Supplementary Sections I–IV and Figs. 1–27. [file 41567_2024_2400_MOESM1_ESM.pdf]

nature physics

Article https://doi.org/10.1038/s41567-024-02400-8

Observation of Josephson harmonics in tunnel junctions

In the format provided by the authors and unedited

Supplementary Information

for

"Observation of Josephson Harmonics in Tunnel Junctions"

CONTENTS

I. Theory 2
A. Theoretical background 2
B. Transparency distributions 5
1. Single channel 5
2. Small transparencies 6
3. Universal distributions from the literature 6
4. Mesoscopic model of barrier inhomogeneity 8
C. Josephson harmonics 9
1. Effective parameterizations 10
2. Additional evidence 13
3. Exhaustive scan 13
4. Charge dispersion 13
5. Hamiltonian 16
6. Perturbative expansion 16
7. Engineering E_{Jm} coefficients 18
D. Alternative corrections 19
1. Series inductance 19
2. Additional hidden modes 22
3. Multi-qubit coupling 23
4. Asymmetry in the superconducting gaps 25

II. Numerical methods 26
A. Inverse eigenvalue problem 26
1. LiPIEP 26
2. HamPIEP 26
B. Choosing appropriate weights 27
C. Identification of dressed states 29
D. Residuals for the Köln data 30

III. Samples description 32
A. KIT 32
B. ENS 34
C. Köln 37
D. IBM 38

IV. Molecular dynamics simulation of junction growth 40
A. System construction 40
B. Molecular dynamics results 40
C. Additional STEM images of JJ barriers 42

I. THEORY

We review the theory of superconducting tunnel junctions, starting from the general current-phase relation as a sum over conduction channels with certain transparencies. Then we derive a closed-form expression for the higher-order Josephson energies and study several transparency distributions. We also introduce a mesoscopic model of tunneling through an inhomogeneous (i.e. non-uniform) barrier, which can describe the non-negligible contributions from higher harmonics to the Josephson effect in regular SIS junctions. Based on these results, we give a motivation for some phenomenological models for the relative size of $E_{\mathrm{J}m}/E_{\mathrm{J}}$ and show results for these alternative models as well as for other transmon data extracted from the literature. Additionally, we perform an exhaustive scan over the higher-order Josephson contributions, yielding the full range of suitable $E_{\mathrm{J}m}/E_{\mathrm{J}}$ for each sample, independent of the particular models discussed. Finally, we study potential alternative corrections, both on a circuit level—such as a stray inductance, additional hidden modes, or the coupling to other qubits—and on the junction level—such as an asymmetry in the superconducting electrodes. We explain why we have come to the conclusion that these corrections cannot account for the mismatch between the datasets and the standard transmon model.

A. Theoretical background

The general formula for the current-phase relation in Josephson tunnel junctions can be written as a sum over conduction channels [S1],

$$I_S(\varphi) = \frac{e\Delta}{2\hbar} \sum_{n=1}^{N} \frac{T_n \sin\varphi}{\sqrt{1 - T_n \sin^2(\varphi/2)}}\,, \tag{S1}$$

where Δ is the superconducting energy gap, N is the number of conduction channels, $T_n \in [0,1]$ are their transparencies (the transmission amplitudes t_n through the channels determine both their transparencies, $T_n = |t_n|^2$, as well as the reflection coefficients for Andreev processes, see e.g. Section 4.4 in [S2] and Fig. 4.7 there), φ is the phase difference across the Josephson junction, e is the electron charge, and $\hbar$ is the reduced Planck quantum. Equation (S1) is Beenakker's multichannel generalization of a formula found by Haberkorn et al. [S3] (see also Ref. [S4] and [S5, Eq. (17)]). We neglect here temperature-dependent corrections as they are exponentially small in $T/T_c \ll 1$.

For tunnel junctions, it is generally assumed that $T_n \ll 1$ for all n [S6, S7], so keeping only the lowest order contribution would give

$$I_S(\varphi) \simeq I_{\mathrm{c}} \sin\varphi\,, \quad I_{\mathrm{c}} = \frac{e\Delta}{2\hbar}\sum_n T_n = \frac{\pi\Delta}{2e} G\,, \tag{S2}$$

where $G = R_N^{-1} = e^2/(\pi\hbar)\sum_n T_n$ is the normal-state conductance of the junction. The corresponding contribution H_{J} to the effective Hamiltonian is found using the relation

$$I(\varphi) = \frac{2e}{\hbar}\frac{dH_{\mathrm{J}}}{d\varphi}\,, \tag{S3}$$

and we recover the Josephson term of the standard transmon model,

$$H_{\mathrm{J}} \simeq -E_{\mathrm{J}} \cos\varphi\,, \quad E_{\mathrm{J}} = \frac{\Delta}{8} g\,, \tag{S4}$$

with $g = G/(e^2/h)$ the dimensionless conductance.

However, in general, Eq. (S1) contains higher harmonics, and the condition $T_n \ll 1$ is not always satisfied for all transmission channels n. To obtain an expression for higher harmonic corrections, we integrate Eq. (S1) and use Eq. (S3) to get the Josephson part of the Hamiltonian

$$H_{\mathrm{J}} = -\Delta \sum_{n=1}^{N} \sqrt{1 - T_n \sin^2\frac{\varphi}{2}}\,. \tag{S5}$$

Since H_{J} is symmetric and 2π periodic in φ, it can be written as a Fourier cosine series

$$H_{\mathrm{J}} = -\sum_{m=1}^{\infty} E_{\mathrm{J}m} \cos(m\varphi)\,. \tag{S6}$$

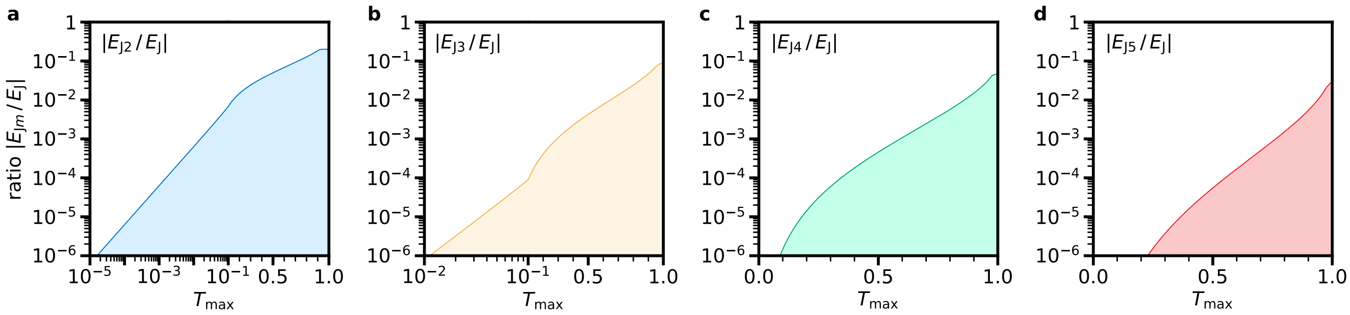

Figure S1. **Bound for the largest transparency $T_{\max}$ contributing to the conduction channels.** For a given ratio $|E_{\mathrm{J}m}/E_{\mathrm{J}}|$ for **a** $m=2$, **b** $m=3$, **c** $m=4$, **d** $m=5$, the line indicates a lower bound for the largest transparency, of which at least one conduction channel must contribute. The area indicates all values $|E_{\mathrm{J}m}/E_{\mathrm{J}}|$ that are possible given $T_{\max}$. Note the change between linear and logarithmic scale at $T_{\max}=0.1$ for the left two panels. An upper limit on the bound is given by the quantum point contact limit (see Section I B 1) which results in the flat part of the curve for $T_{\max}$ close to 1.

To derive an analytic expression for the $E_{\mathrm{J}m}$, we apply the relations

$$\sqrt{1+x}=\sum_{m=0}^{\infty}\binom{2m}{m}\frac{(-1)^{m+1}}{4^m(2m-1)}x^m\,, \qquad \sin^{2m}\frac{x}{2}=\frac{1}{2^{2m-1}}\sum_{j=0}^{m-1}(-1)^{m-j}\binom{2m}{j}\cos((m-j)x)\,, \tag{S7}$$

for $m\geq 1$ to Eq. (S5). We obtain

$$H_{\mathrm{J}}=-\sum_{m=1}^{\infty}\sum_{n=1}^{N}\left(4\Delta(-1)^{m+1}\sum_{k=0}^{\infty}\binom{2k+2m-2}{k+m-1}\binom{2k+2m}{k}\frac{1}{k+m}\left(\frac{T_n}{16}\right)^{k+m}\right)\cos(m\varphi) \tag{S8}$$

$$=-\sum_{m=1}^{\infty}\sum_{n=1}^{N}\left((-1)^{m+1}\frac{4\Delta}{m}\binom{2m-2}{m-1}{}_2F_1(m-1/2,m+1/2;2m+1;T_n)\left(\frac{T_n}{16}\right)^m\right)\cos(m\varphi)\,, \tag{S9}$$

where ${}_2F_1$ denotes the hypergeometric function which is defined by (see e.g. [S8, Section 16.2])

$${}_pF_q(a_1,...,a_p;b_1,...,b_q;x)=\sum_{k=0}^{\infty}\frac{(a_1)_k\cdots(a_p)_k}{(b_1)_k\cdots(b_q)_k}\frac{x^k}{k!}\,, \tag{S10}$$

with $(a)_k=a(a+1)(a+2)\cdots(a+k-1)$ denoting the Pochhammer symbol. Thus we find an expression for the higher-order contributions to the effective Josephson Hamiltonian,

$$E_{\mathrm{J}m}=(-1)^{m+1}\frac{4\Delta}{m}\binom{2m-2}{m-1}\sum_{n=1}^{N}{}_2F_1(m-1/2,m+1/2;2m+1;T_n)\left(\frac{T_n}{16}\right)^m\,. \tag{S11}$$

Note that, for small transparencies $T_n\ll 1$ where it is sufficient to consider only the leading-order term of ${}_2F_1$, we recover $E_{\mathrm{J}1}=E_{\mathrm{J}}=\Delta g/8$ and the sinusoidal current-phase relation (in this section, we identify $E_{\mathrm{J}1}\equiv E_{\mathrm{J}}$).

We can derive a bound for the largest possible ratio $|E_{\mathrm{J}m}/E_{\mathrm{J}}|$ when all conduction channels have a transparency $T_n\leq T_{\max}$, independent of the particular distribution of transparencies $\{T_n\}$. Using the monotonicity of ${}_2F_1$, we have in the numerator ${}_2F_1(\cdots;T_n)\leq {}_2F_1(\cdots;T_{\max})$ for all n, and in the denominator ${}_2F_1(\cdots;T_n)\geq 1$ for all n. For the remaining quotient of sums over n, we use $\sum_n T_n^m/\sum_n T_n=T_{\max}^{m-1}[\sum_n(T_n/T_{\max})^m/\sum_n(T_n/T_{\max})]\leq T_{\max}^{m-1}$. Thus we obtain

$$\left|\frac{E_{\mathrm{J}m}}{E_{\mathrm{J}}}\right|\leq\frac{1}{m}\binom{2m-2}{m-1}{}_2F_1(m-1/2,m+1/2;2m+1;T_{\max})\left(\frac{T_{\max}}{16}\right)^{m-1}\,. \tag{S12}$$

The bound is shown in Fig. S1 for the leading ratios. It yields, for instance, the following statement: Given some observation for $|E_{\mathrm{J}2}/E_{\mathrm{J}}|\approx 2.40\,\%$, there must at least be one conduction channel with a transparency $T_{\max}\geq 0.298$. We remark that the estimation of $T_{\max}$ in this way is based on the general current-phase relation hypothesis Eq. (S1). In the presence of harmonics from a stray inductance such as $L=0.5\,\mathrm{nH}$ (see Section I D 1 below), the bound would

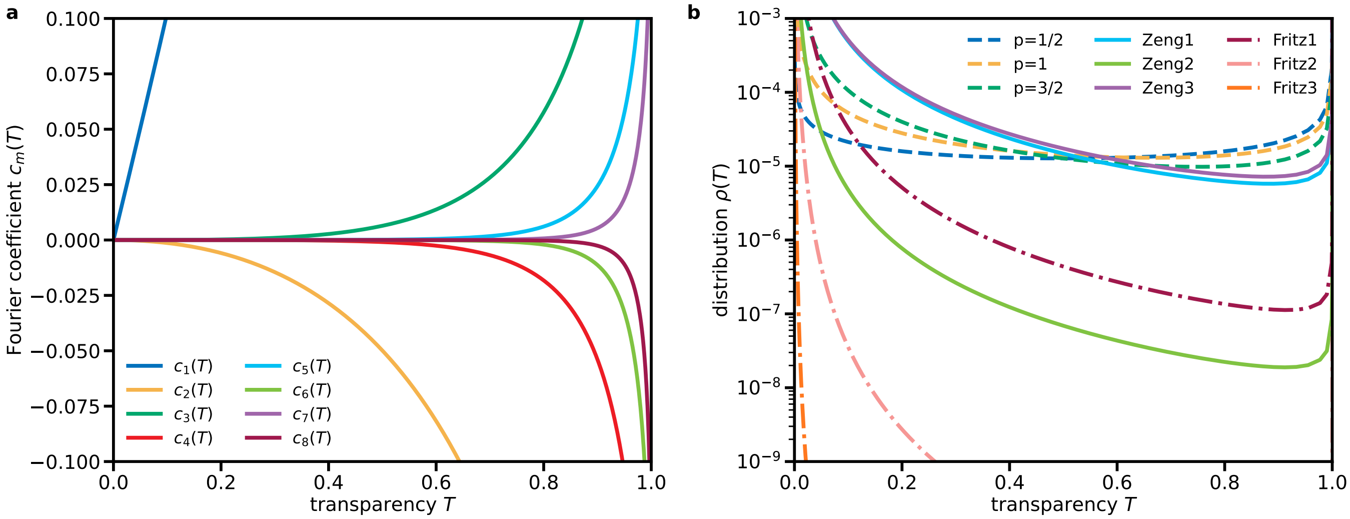

Figure S2. **Fourier coefficients $c_m(T)$ and several distributions $\rho(T)$ to describe the contribution of higher harmonics to the Josephson effect.** **a** Fourier coefficients $c_m(T)$ of the series expansion of the general current-phase relation given by Eq. (S14). **b** Distribution of transparencies $\rho(T)$ for the occasionally considered universal form Eq. (S31) (dashed lines), and the mesoscopic model in Eq. (S38) using average barrier thicknesses $\bar{d}$ and standard deviations σ extracted from Ref. [S10] (Zeng, solid lines) and Ref. [S11] (Fritz, dash-dotted lines); the corresponding junction parameters are also listed in Table S2. As some of the distributions given by Eq. (S31) are not normalizable, the prefactors have been chosen such that $\langle T\rangle = 10^{-5}$ to make them comparable (i.e., $n_{1/2} = 2/\pi \times 10^{-5}$, $n_1 = 1/2 \times 10^{-5}$, and $n_{3/2} = 1/\pi \times 10^{-5}$, cf. [S12]). Note that all distributions have two peaks, namely the bulk contribution for very small transparencies near $T = 0$ and a few very high transparencies near $T = 1$ responsible for non-negligible Josephson harmonics.

be $T_{\max} \geq 0.096$. We note that high-transparency conduction channels with energy as in Eq. (S5) have been observed experimentally, for example in break junctions (see [S9], in which the authors identify a channel with $T = 0.99217$).

In general, the current-phase relation as a function of the Josephson energies $E_{\mathrm{J}m}$ reads

$$I_S(\varphi) = \frac{2e}{\hbar} \sum_{m=1}^{\infty} m E_{\mathrm{J}m} \sin(m\varphi)\,. \tag{S13}$$

The analysis presented in Fig. 3c of the main text shows that the corrections to the sinusoidal current-phase relation are on the level of several percent for all experiments under consideration. For given $E_{\mathrm{J}m}$, the critical current I_{c} can be obtained by finding the maximum of Eq. (S13) as a function of φ (we show three limiting cases of $I_S(\varphi)/I_{\mathrm{c}}$ for each sample in the insets of Fig. S7 below; values for I_{c} are given in Table S3).

We can use the result for $E_{\mathrm{J}m}$ in Eq. (S11) to obtain a closed-form expression for the Fourier coefficients $c_m(T_n)$ of the Josephson supercurrent discussed in the main text,

$$I_S(\varphi) = \frac{e\Delta}{2\hbar} \sum_{m=1}^{\infty} \sum_{n=1}^{N} c_m(T_n) \sin(m\varphi)\,, \tag{S14}$$

yielding

$$c_m(T) = \binom{2m-2}{m-1} \frac{(-1)^{m+1} T^m}{16^{m-1}}\, {}_2F_1(m - 1/2, m + 1/2; 2m+1; T)\,, \tag{S15}$$

with $E_{\mathrm{J}m} = \frac{\Delta}{4}\frac{1}{m}\sum_{n=1}^{N} c_m(T_n)$. The first eight Fourier coefficients are shown in Fig. S2a. For the first three of them, we give here their series expansion for small transparencies,

$$c_1(T) = T + \frac{T^2}{4} + \frac{15T^3}{128} + \mathcal{O}\left(T^4\right)\,, \tag{S16a}$$

$$c_2(T) = -\left[\frac{T^2}{8} + \frac{3T^3}{32} + \frac{35T^4}{512} + \mathcal{O}\left(T^5\right)\right]\,, \tag{S16b}$$

$$c_3(T) = \frac{3T^3}{128} + \frac{15T^4}{512} + \frac{945T^5}{2^{15}} + \mathcal{O}\left(T^6\right)\,. \tag{S16c}$$

We note that successive coefficients are always alternating in sign, and for the leading terms we have

$$\frac{c_{m+1}(T)}{c_m(T)} \sim -\frac{m-1/2}{4m} T\,, \tag{S17}$$

so the contribution of higher harmonics becomes more relevant for larger transparencies. Furthermore, the prefactor in this ratio is of order unity and converges to $-1/4$ for large m. The properties of $c_m(T)$ propagate to the Josephson energies, for which we have

$$|E_{\mathrm{J}m}| = \frac{\Delta}{4}\frac{1}{m}\sum_{n=1}^{N} \underbrace{(-1)^{m+1}c_m(T_n)}_{\text{positive, } < (-1)^m c_{m-1}(T_n)} < |E_{\mathrm{J}(m-1)}|\,. \tag{S18}$$

Thus we expect that the Josephson energies $E_{\mathrm{J}m}$ for a physical model alternate in sign and decrease in magnitude for increasing order m.

B. Transparency distributions

For a large number of channels N, summing over the channels corresponds to averaging over a distribution of transparencies $\rho(T)$,

$$E_{\mathrm{J}m} = \frac{\Delta}{4}\frac{1}{m}\sum_{n=1}^{N} c_m(T_n) = \frac{\Delta}{4}\frac{N}{m}\int_0^1 dT\,\rho(T)c_m(T)\,. \tag{S19}$$

The ratio $E_{\mathrm{J}m}/E_{\mathrm{J}}$ can then be expressed as

$$\frac{E_{\mathrm{J}m}}{E_{\mathrm{J}}} = \frac{1}{m}\frac{\int_0^1 dT\rho(T)c_m(T)}{\int_0^1 dT\rho(T)c_1(T)}\,. \tag{S20}$$

If the transparency distribution $\rho(T)$ is not narrowly peaked around a small average value or contains significant weight for higher transparencies, it is typically not justified to neglect higher harmonics. In this section, we look at several example distributions for which this is the case.

1. *Single channel*

A very elementary case is a single conduction channel with arbitrary transparency T_1. In this case, the formal distribution would be $\rho(T) = \delta(T - T_1)$, but the solution can directly be obtained from Eq. (S11) for $N = 1$,

$$E_{\mathrm{J}m} = (-1)^{m+1}\frac{4\Delta}{m}\binom{2m-2}{m-1}\,{}_2F_1(m-{}^1\!/\!_2, m+{}^1\!/\!_2; 2m+1; T_1)\frac{T_1^m}{16^m}\,. \tag{S21}$$

For the ratio of Josephson energies, we find accordingly

$$\frac{E_{\mathrm{J}m}}{E_{\mathrm{J}}} = \frac{(-1)^{m+1}}{m}\binom{2m-2}{m-1}\frac{T_1^{m-1}}{16^{m-1}}\frac{{}_2F_1(m-{}^1\!/\!_2, m+{}^1\!/\!_2; 2m+1; T_1)}{{}_2F_1({}^1\!/\!_2, {}^3\!/\!_2; 3; T_1)}\,. \tag{S22}$$

We consider two important limits for $|E_{\mathrm{J}m}/E_{\mathrm{J}}|$, namely the fully open quantum point contact with $T_1 = 1$ as an upper limit, and a homogeneous barrier of very small transparency $T_1 \ll 1$ as a lower limit.

For the point contact, setting $T_1 = 1$ in Eq. (S22) yields

$$\frac{E_{\mathrm{J}m}}{E_{\mathrm{J}}} = (-1)^{m+1}\frac{3}{4m^2-1}\,. \tag{S23}$$

This means that asymptotically, the ratios $|E_{\mathrm{J}m}/E_{\mathrm{J}}|$ should not scale weaker than $1/m^2$. Furthermore, in the case of a point contact, Eq. (S21) yields an upper limit for the first Josephson energy: For $m = 1$, we have $E_{\mathrm{J}} \approx 0.4244\Delta$. Assuming an energy gap of $\Delta/h = 50\,\mathrm{GHz}$ (see [S13]), we obtain $E_{\mathrm{J}}/h \approx 21.22\,\mathrm{GHz}$, which is of the same order as the values of E_{J} given in Table S3 below. We remark that the point contact $T_1 = 1$ also corresponds to the KO-2

model by Kulik and Omelyanchuk [S14], which is the clean, fully ballistic counterpart [S5] of the KO-1 model [S15] discussed below.

For the lower limit with $T_1 \ll 1$, taking the leading-order term in Eq. (S22) yields

$$\frac{E_{\mathrm{J}m}}{E_{\mathrm{J}}} \approx \frac{(-1)^{m+1}}{m} \binom{2m-2}{m-1} \frac{T_1^{m-1}}{16^{m-1}} . \tag{S24}$$

The binomial coefficient is bounded by

$$2^{m-1} \leq \binom{2m-2}{m-1} \leq 4^{m-1} , \tag{S25}$$

and Stirling's approximation yields for large m

$$\binom{2m-2}{m-1} \sim \frac{4^{m-1}}{\sqrt{(m-1)\pi}} \approx \frac{4^{m-1}}{\sqrt{m\pi}} . \tag{S26}$$

Applying this approximation to the expression in Eq. (S24) yields

$$\frac{E_{\mathrm{J}m}}{E_{\mathrm{J}}} \approx (-1)^{m+1} \frac{(T_1/4)^{m-1}}{\sqrt{\pi} m^{3/2}} . \tag{S27}$$

2. *Small transparencies*

Another simple but probably only pedagogical case is a uniform distribution of transparencies below some cutoff value T_0. We model this case by $\rho(T) = \nu\, \Theta(T_0 - T)$, where Θ denotes the step function and ν is the normalization. We then find

$$E_{\mathrm{J}m} = (-1)^{m+1} \frac{\Delta}{4} \frac{N}{m} \frac{\nu}{m+1} \binom{2m-2}{m-1} \frac{T_0^{m+1}}{16^{m-1}} {}_3F_2(m - 1/2, m + 1/2, m+1; 2m+1, m+2; T_0) , \tag{S28}$$

and for the ratio

$$\frac{E_{\mathrm{J}m}}{E_{\mathrm{J}}} = (-1)^{m+1} \frac{1}{m} \frac{1}{m+1} \binom{2m-2}{m-1} \frac{T_0^{m}}{16^{m-2}} \frac{{}_3F_2(m - 1/2, m + 1/2, m+1; 2m+1, m+2; T_0)}{{}_3F_2(1/2, 3/2, 2; 3, 3; T_0)} . \tag{S29}$$

If the cutoff value is very small, i.e., all transparencies $T \leq T_0 \ll 1$ are very low and it is sufficient to consider only the lowest-order term, we find

$$\frac{E_{\mathrm{J}m}}{E_{\mathrm{J}}} = \frac{(-1)^{m+1}}{m(m+1)} \binom{2m-2}{m-1} \frac{T_0^{m}}{16^{m-2}} . \tag{S30}$$

Note that this expression is similar to the case of a homogeneous barrier in Eq. (S24), but the exponent of T_0 is larger by one.

3. *Universal distributions from the literature*

There are regimes in which the transparency distribution $\rho(T)$ is expected to take a universal form, independent of the details of the junction (see e.g. [S5, S12, S16, S17] and references therein):

$$\rho(T) = \frac{n_p}{T^p \sqrt{1-T}} , \tag{S31}$$

where n_p is a normalization factor, and $p = 1/2, 1, 3/2$ enumerates three distinct cases which we discuss in the following (the corresponding distributions are shown in Fig. S2b). As we show in this work, typical qubit tunnel junctions are not in any of the universal regimes, so their microscopic and mesoscopic properties matter.

The case $p = 1/2$ represents a chaotic dot connected via identical ballistic point contacts to two superconducting leads [S18]. The case $p = 1$, also known as the KO-1 model by Kulik and Omelyanchuk [S15], represents a diffusive quasi-1D wire of length L longer than the Fermi wavelength, $L \gg \lambda_F$, but still in the short-junction regime $L \ll \sqrt{\xi_0 l}$,

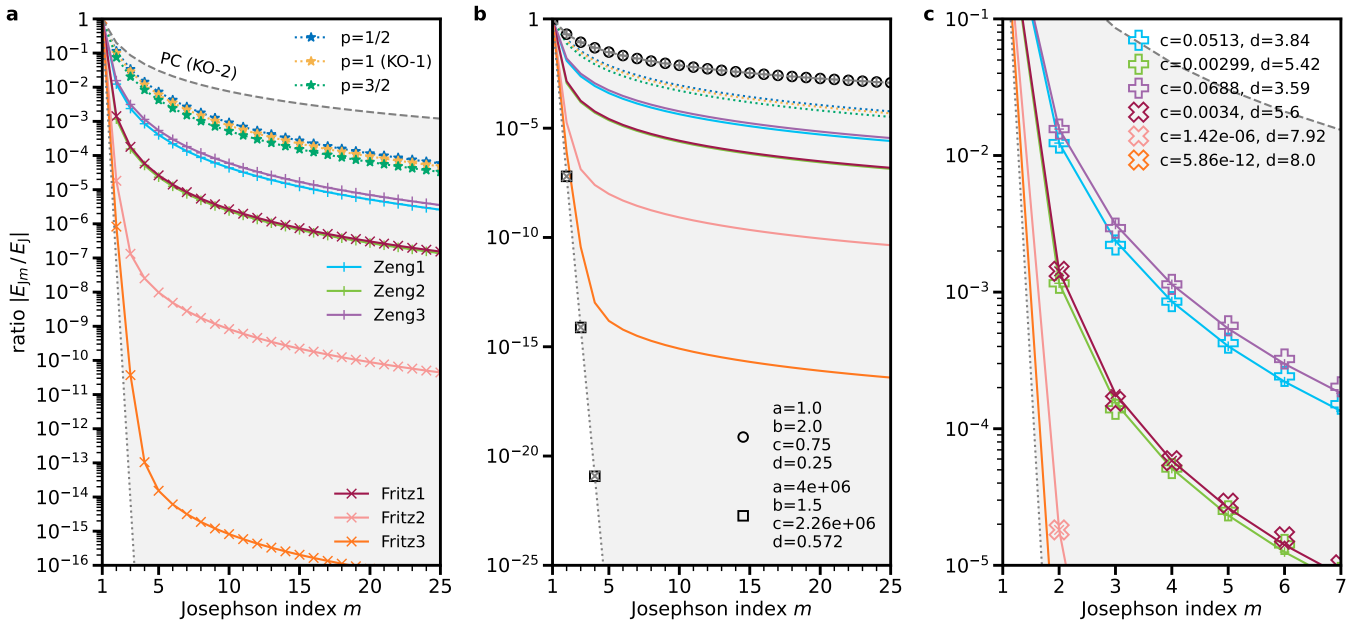

Figure S3. **Comparison of the scaling of the Josephson energy ratio $|E_{\mathrm{J}m}/E_{\mathrm{J}}|$ for various models. a** We show the ratios in Eq. (S33) for the universal distribution Eq. (S31), as well as the ratios computed for the mesoscopic $(\bar{d},\sigma)$ model using parameters for the average barrier thickness $\bar{d}$ and the standard deviation σ extracted from the literature [S10, S11] (see Table S2). **b** The (a,b,c,d) model in Eq. (S41) can reproduce the upper and lower limits, namely the point contact (black circles, see Eq. (S42), where the reference result from Eq. (S23) is shown with gray plusses) and the homogeneous barrier of small transparency (black squares, see Eq. (S43), where the reference result from Eq. (S22) for $T_1 = 10^{-6}$ is shown with gray crosses). As a reference, results from the mesoscopic model given by Eq. (S38) for the samples listed in Table S2 are shown with solid lines and results from the universal distribution are shown with dotted lines (see legend of panel a). **c** The (a,b,c,d) model in Eq. (S41) can approximately reproduce the ratios predicted by the mesoscopic $(\bar{d},\sigma)$ model. Since there is no visible exponential decay for the $(\bar{d},\sigma)$ model (see panel a), $a=1$ is always fixed and $b=3$ is determined from an asymptotic fit, leaving only c and d as free parameters. The resulting values are given in the legend. Colors correspond to the same cases as in panel a. Hollow symbols lie on top of the plusses and crosses if the approximation in terms of the (a,b,c,d) model is good.

where ξ_0 is the superconducting coherence length and l the electron mean free path with $l \ll \xi_0$; the wire transverse size is $\ll L$ [S5]. It has been implemented with aluminum nanobridges [S19–S21] and has been proposed to describe transmons based on superconductor-constriction-superconductor (ScS) junctions [S22]. The case $p=3/2$ represents a disordered interface ($L \ll \lambda_F$) [S12] and it could be relevant to semiconductor quantum well junctions [S23].

Evaluating Eq. (S19) with the distribution Eq. (S31) yields the higher-order Josephson energies

$$E_{\mathrm{J}m} = \frac{\Delta}{4}\frac{N}{m}n_p\frac{(-1)^{m+1}}{16^{m-1}}\binom{2m-2}{m-1}\sqrt{\pi}\frac{\Gamma(m-p+1)}{\Gamma(m-p+3/2)}{}_3F_2(m-{}^1\!/\!_2, m+{}^1\!/\!_2, m-p+1; 2m+1, m-p+{}^3\!/\!_2; 1) \quad \text{(S32)}$$

$$= \frac{\Delta}{4}\frac{N}{m}n_p \begin{cases} \dfrac{(-1)^{m+1}}{16^{m-1}}\dbinom{2m-2}{m-1}\dbinom{2m}{m}\dfrac{\pi}{4^m}{}_3F_2(m-{}^1\!/\!_2, m+{}^1\!/\!_2, m+{}^1\!/\!_2; 2m+1, m+1; 1) & (p=\tfrac{1}{2}) \\ (-1)^{m+1}\dfrac{8}{4m^2-1} & (p=1) \\ \dfrac{(-1)^{m+1}}{16^{m-1}}\dbinom{2m-2}{m-1}\dbinom{2m-2}{m-1}\dfrac{\pi}{4^{m-1}}{}_3F_2(m-{}^1\!/\!_2, m+{}^1\!/\!_2, m-{}^1\!/\!_2; 2m+1, m; 1) & (p=\tfrac{3}{2}) \end{cases} . \quad \text{(S33)}$$

The absolute ratios $|E_{\mathrm{J}m}/E_{\mathrm{J}}|$ up to $m=25$ are shown in Fig. S3a. Interestingly, in all cases the contribution of the second Josephson energy is approximately 10 % (we have $E_{\mathrm{J}2}/E_{\mathrm{J}} \approx -11\,\%, -10\,\%, -7.5\,\%$ for $p=1/2, 1, 3/2$). The increase in absolute value of the ratio with decreasing p is due to the relatively lower weight given to low transparencies in the respective transparency distributions (see upper limits in Fig. S2b).

Table S1. Parameters for AlO_x barriers taken from the indicated references. m_i/m is the effective electron-mass ratio, ϕ is the average height of the potential barrier, and a and d_0 are parameters for the tunnel-transmission probability in Eq. (S35).

Reference	m_i/m	ϕ (eV)	a^2	d_0 (nm)
[S28, S29]	0.44	2.05	2.75	0.206
[S24]	1	0.84	2.99	0.213

The closed-form Josephson harmonics expansion for $p = 1$ in Eq. (S33) also provides an easy way to construct the KO-1 Hamiltonian in the charge basis (in which the $\cos(m\varphi)$ term describes the mth subdiagonal),

$$H_{\text{J}}^{\text{KO-1}} = \sum_{m=1}^{\infty} E_{\text{J}1}(-1)^m \frac{3}{m(4m^2-1)} \cos(m\varphi) \,. \tag{S34}$$

This form agrees with the Josephson part of the ScS Hamiltonian $E_{\text{J}}'(2\ln(\cos^2(\varphi/2)) + 4\sin(\varphi/2)\tanh^{-1}(\sin(\varphi/2)))$ given in [S22] (after the substitution $E_{\text{J}}' = 3E_{\text{J}1}/4$) where it was approximately diagonalized using finite differences in φ space.

4. *Mesoscopic model of barrier inhomogeneity*

In this section, we derive a transparency distribution $\rho(T)$ from a model of an inhomogeneous tunnel barrier. To this end, we describe the charge transport through the insulating barrier of a JJ in terms of tunneling processes through a potential barrier with a non-uniform thickness distribution $\rho(d)$. We expect the result to be applicable to "regular" barriers, in the sense that the thickness d of the barrier between the leads can be described by a Gaussian distribution with average thickness $\bar{d}$ and standard deviation σ (cf. Fig. 3c of the main text and the STEM images and the molecular dynamics simulations in Section IV).

Tunneling through AlO_x barriers has previously been studied using a trapezoidal barrier model [S24, S25]. Here we use the simpler rectangular barrier model with height given by the average height of the trapezoidal barrier. This is a good approximation up to corrections quadratic in the ratio of the difference over the average height ϕ (as measured from the Fermi energy E_F). For a rectangular barrier of thickness d, the textbook result for the transmission probability T is [S26]

$$T = \frac{1}{1 + a^2 \sinh^2(d/d_0)} \,, \tag{S35}$$

with

$$a^2 = \frac{1}{4}\left(\sqrt{\frac{m_i\phi}{mE_F}} - \sqrt{\frac{mE_F}{m_i\phi}}\right)^2 , \quad d_0 = \hbar/\sqrt{2m_i\phi} \,, \tag{S36}$$

and where we have assumed tunneling electrons to have the Fermi energy. For aluminum, within the free electron model, m is the electron mass and $E_F = 11.67\,\text{eV}$. The parameter m_i denotes the effective mass in the band of the insulator closest to the energy of the tunneling electrons; m_i enters Eq. (S36) as a prefactor for ϕ, so only the product of the two quantities is relevant. From the literature, see Table S1, we estimate $a^2 = 2.87$ and $d_0 = 0.21\,\text{nm}$ (we caution the reader that the parameters can depend on how the oxide is interfaced with the aluminum layer [S27]).

Since measurements of the barrier thickness d have been well described by a Gaussian distribution [S10], we consider the distribution

$$\rho(d) = \frac{2\Theta(d)}{1 + \text{Erf}(\bar{d}/\sqrt{2}\sigma)} \frac{1}{\sqrt{2\pi}\sigma} e^{-\left(d-\bar{d}\right)^2/\left(2\sigma^2\right)} \,, \tag{S37}$$

where Θ is the step function and Erf is the error function. The prefactor of the Gaussian ensures normalization and that $d \geq 0$. Inverting Eq. (S35) to get the thickness d as a function of the transparency T, one can then find the probability density for the transparency,

$$\rho(T) = \frac{2}{1 + \text{Erf}(\tilde{d}/\sqrt{2}\tilde{\sigma})} \frac{1}{2T\sqrt{1-T}\sqrt{1-T+a^2T}} \frac{1}{\sqrt{2\pi}\tilde{\sigma}} e^{-(f(T)+\alpha)^2/(2\tilde{\sigma}^2)} \,, \tag{S38}$$

where $\tilde{d} = \bar{d}/d_0$, $\tilde{\sigma} = \sigma/d_0$, and

$$f(T) = \log \frac{\sqrt{T}}{\sqrt{1-T} + \sqrt{1-T+a^2T}}\,, \quad \alpha = \tilde{d} + \log a\,. \tag{S39}$$

For given $(\tilde{d}, \tilde{\sigma})$, the Josephson harmonics $E_{\mathrm{J}m}$ can be obtained by evaluating Eq. (S20) with the distribution $\rho(T)$ of Eq. (S38). Examples for the absolute ratios $|E_{\mathrm{J}m}/E_{\mathrm{J}}|$ for the samples listed in Table S2 are shown in Fig. S3a. Results for the KIT, ENS, and Köln experiments are shown in Fig. S4 and Table S3 below. Note that in this model a non-negligible ratio $\sigma/\bar{d}$ indicates that regions of smaller thickness and thus high-transparency conduction channels are likely, which is not completely unexpected given the inhomogeneity of AlO_x barriers (see the STEM images in Section IV).

For $a \gg 1$ in Eq. (S35), at leading order the distribution in Eq. (S38) reduces for $T \gg 1/(1+a^2)$ to that in Eq. (S31) with $p = 3/2$. However, it takes log-normal form for $T \ll 1/(1+a^2)$, indicating that the channels with low transparencies are responsible for a deviation from universality; for Al/AlO_x/Al, a^2 is not very large, so one should not expect to observe any universal behavior.

We can now investigate when a junction can be considered a "good" tunnel junction, meaning not only $\langle T \rangle \ll 1$ but also $\langle T^2 \rangle / \langle T \rangle \ll 1$. The latter is a necessary condition to be able to neglect higher harmonics, although one should also check what happens for higher moments; the second condition is not satisfied for the universal distributions. For the transparency distribution $\rho(T)$ in Eq. (S38), we have approximately

$$\langle T^n \rangle \simeq \frac{1}{1 - \mathrm{Erfc}(\bar{d}/\sqrt{2}\sigma)/2} \left(\frac{4}{a^2}\right)^n e^{\frac{2n\sigma}{d_0}\left(\frac{n\sigma}{d_0} - \frac{\bar{d}}{\sigma}\right)} \left[1 - \frac{1}{2}\mathrm{Erfc}\left(\frac{\bar{d}}{\sqrt{2}\sigma} - \frac{\sqrt{2}n\sigma}{d_0}\right)\right]\,, \tag{S40}$$

where $\mathrm{Erfc} = 1 - \mathrm{Erf}$ is the complementary error function. For a sharp barrier, $\sigma \ll \sqrt{\bar{d}d_0}$, this reduces to $\langle T^n \rangle \simeq (4/a^2)^n e^{-2n\bar{d}/d_0}$ (assuming n not too large, so that the Erfc factors can be negligible), and a junction with $\bar{d} \gg d_0$ would indeed behave as a "good" tunnel junction. For AlO_x barriers, since $4/a^2$ is of order unity, the condition $\langle T \rangle \ll 1$ reduces to $\bar{d}/d_0 \gg \max\{1, 2(\sigma/d_0)^2\}$, while $\langle T^2 \rangle / \langle T \rangle \ll 1$ is satisfied if $\bar{d}/d_0 \gg \max\{1, 4(\sigma/d_0)^2\}$; therefore, assuming $\sigma/d_0 \gtrsim 1$, we also need $\bar{d}/d_0 \gg 4(\sigma/d_0)^2$ (note that since these ratios are in the argument of the exponential, the large inequality sign "$\gg$" can mean a factor of 2 or 3, not orders of magnitude). Based on direct observations on three samples [S10], the latter condition is not met, which implies that $E_{\mathrm{J}2}/E_{\mathrm{J}}$ can be non-negligible, see Table S2. It should be noted that barriers of similar thickness and smaller standard deviation can be fabricated if proper care is taken [S11]; those junctions have $\tilde{d} > 4\tilde{\sigma}^2$, see Table S2. They also have $\langle T \rangle \ll 1$ and $\langle T^2 \rangle / \langle T \rangle \ll 1$, but $\langle T^2 \rangle / \langle T \rangle > \langle T \rangle$, meaning that significant fluctuations in transparency are nonetheless present.

As an additional check of our approach, we note that the distribution of transparencies could explain the observed magnitude of the subgap current in SIS and SIN junctions, see e.g. [S30] and references therein. For a narrow distribution of transparencies, the ratio G_{sg}/G_N between subgap conductance and normal-state conductance should be of the order of the average transparency, since $G_{sg} \propto T^2$ and $G_N \propto T$. For the data in Fig. 2 of [S30], we can convert the ratio G_N/A into average transparencies by multiplying it by $\lambda_F^2/G_Q \approx 1.6 \times 10^{-9}\,\Omega\,\mathrm{mm}^2$; this means that for the Al-AlO_x-Al junctions, $\langle T \rangle$ varies roughly between 7×10^{-7} and 10^{-4}. Over that range, the ratio G_{sg}/G_N goes from about 3×10^{-5} to 5×10^{-3}. At the lower end of this range, the pair of numbers is similar to that of sample Fritz3 in Table S2. Assuming for simplicity $G_{sg}/G_N = \langle T^2 \rangle / \langle T \rangle$, we can match the observation by taking $\bar{d}/d_0 = 8.19$ and $\sigma/d_0 = 0.97$. Then decreasing $\bar{d}$ while keeping constant σ as to get $\langle T \rangle \sim 10^{-4}$, we get $\langle T^2 \rangle / \langle T \rangle \sim 4 \times 10^{-3}$, in fair agreement with the upper end of the observed range (keeping the standard deviation σ constant is consistent with the finding of Ref. [S11] that it is correlated with variations in thickness of the bottom aluminum layer, whose fabrication was presumably not changed from junction to junction in Ref. [S30]).

Our mesoscopic model can describe the harmonics in the Josephson junctions of KIT, ENS and Köln very well (see Fig. 3b in the main text). For the IBM junctions, the Josephson harmonics are much larger and stay relevant until high order (cf. Fig. S5 below). They are close to the point contact limit, which asymptotically scales as $1/m^2$. The mesoscopic model, however, shows a stronger decay (see Fig. S3a) that cannot reproduce the large contributions required up to $E_{\mathrm{J}6}$ and thus cannot describe the observed spectra very well. A physical reason for this may lie in the fact that the assumption of a Gaussian thickness distribution with only two parameters is too simple to model the corresponding barriers. We note that for the samples for which the model works well, the fit mostly constrains the ratio $\bar{d}/\sigma$ rather than their absolute values.

C. Josephson harmonics

In this section, we test several models motivated by the discussion of transparency distributions in the previous section. Additionally, we apply these models to previously published transmon spectra that can be found in the

Table S2. Values for the average thickness $\bar{d}$ and the standard deviation σ of AlO_x barriers in tunnel junctions found in the literature. Shown are the first three samples reported in Ref. [S10] (Zeng) and Ref. [S11] (Fritz), respectively. Columns 4–7 contain the corresponding unitless quantities, $\tilde{d} = \bar{d}/d_0$ and $\tilde{\sigma} = \sigma/d_0$, in terms of $d_0 = 0.21\,\text{nm}$ [S27]. The quantities in columns 8–10 contain the moments $\langle T \rangle$ and $\langle T^2 \rangle / \langle T \rangle$ as well as the ratio E_{J2}/E_J, which have been computed using Eq. (S38).

Sample	$\bar{d}$ (nm)	σ (nm)	$\tilde{d}$	$\tilde{\sigma}$	$\tilde{d}/\tilde{\sigma}$	$4\tilde{\sigma}^2$	$\langle T \rangle$	$\langle T^2 \rangle / \langle T \rangle$	E_{J2}/E_J
Zeng1 [S10]	1.66	0.35	7.90	1.67	4.74	11.11	4.6×10^{-5}	0.098	−0.012
Zeng2 [S10]	1.88	0.32	8.95	1.52	5.87	9.29	2.4×10^{-6}	0.011	−0.0012
Zeng3 [S10]	1.73	0.37	8.24	1.76	4.68	12.42	4.3×10^{-5}	0.120	−0.016
Fritz1 [S11]	1.62	0.29	7.71	1.38	5.59	7.63	1.3×10^{-5}	0.014	−0.0014
Fritz2 [S11]	1.65	0.23	7.86	1.10	7.17	4.80	2.3×10^{-6}	2.8×10^{-4}	-1.8×10^{-5}
Fritz3 [S11]	1.73	0.19	8.24	0.90	9.11	3.27	5.0×10^{-7}	1.3×10^{-5}	-8.3×10^{-7}

literature. Subsequently, we report the scan of the full range of Josephson harmonics for all samples considered in the main text, to distill the maximum information about the Josephson harmonics from the available experimental data. Finally we discuss properties of the Josephson harmonics model such as the charge dispersion, the Hamiltonian, the Josephson potential and current-phase relations, a perturbative expansion, and how engineering Josephson harmonics can both reduce the charge dispersion and increase the anharmonicity.

1. *Effective parameterizations*

The results for E_{Jm} for the transparency distributions $\rho(T)$ considered in the previous section suggest that a suitable parameterization of E_{Jm}/E_J should be (i) alternating in sign, (ii) decreasing in absolute magnitude, and (iii) should allow for both exponential and power-law decay. A suitable parameterization for $m \geq 2$ that also includes the limits given by Eqs. (S23) and (S27) is

$$\frac{E_{Jm}}{E_J} = (-1)^{m+1} \frac{c}{a^m (m^b - d)} , \tag{S41}$$

where (a, b, c, d) are the model parameters to be determined. The point contact given by Eq. (S23) corresponds to

$$a = 1 , \quad b = 2 , \quad c = \frac{3}{4} , \quad d = \frac{1}{4} , \tag{S42}$$

and the lower bound of a homogeneous barrier with small transparency given by Eq. (S27) corresponds to

$$a = \frac{4}{T_1} , \quad b = \frac{3}{2} , \quad c = \frac{4}{\sqrt{\pi} T_1} , \quad d = 2\sqrt{2} - \frac{4}{\sqrt{\pi}} , \tag{S43}$$

where we used the remaining freedom in d to match E_{J2}/E_J. Both are shown in Fig. S3b which confirms that the (a, b, c, d) model can cover the limiting cases. Furthermore, as Fig. S3c shows, the (a, b, c, d) model can approximately reproduce the mesoscopic $(\bar{d}, \sigma)$ model introduced in Section I B 4. Note that, although a and c are related to the inverse transparency in Eq. (S43), this is not necessarily the case when the model is fitted to given spectroscopy data.

In addition to the (a, b, c, d) model in Eq. (S41), we consider the simpler (a, b) model with $c = 1$ and $d = 0$ fixed and given by

$$\frac{E_{Jm}}{E_J} = (-1)^{m+1} \frac{1}{a^m m^b} . \tag{S44}$$

Evidently, this choice captures the most prominent features, namely the power-law scaling of the free quantum point contact as well as an asymptotic exponential scaling towards zero as present in the lower limit of a homogeneous barrier with small transparency. In terms of fitting, we note that a data set might be describable by various combinations of a and b that differ by orders of magnitude. The reason is that, while the model is inspired by the asymptotic scaling as a function of m, for an experiment only the first E_{Jm} coefficients may be relevant, and e.g. the same E_{J2} can be obtained by various combinations of a and b.

We emphasize in this context that, although one might want to ascribe some meaning to the model parameters (e.g. a and b), they are internal, unobservable variables (in the sense of the inverse problem, cf. [S31, S32]). There can

Table S3. Model parameters of all models presented in Fig. S4. The ratios E_{Jm}/E_J and the parameters (a, b, c, d) of the models discussed in Section I C 1 are unitless, $\bar{d}$ and σ of the mesoscopic model discussed in Section I B 4 are given in nanometers, I_c is given in nanoamperes, and all other parameters are given in gigahertz. Gray entries denote derived model properties that are computed from the model parameters. Note that for the KIT results, the bare resonator frequencies Ω agree very well with the high-power cavity frequency given in Figs. S19a and S21a below, see also [S33]). In the second-last row, we show results for the Köln sample (note that E_J is not fixed but tuned according to the magnetic field bias). In the last row, we show representative results for qubit 0 of the IBM Hanoi device (additional data is available in the repository [S34]). Note that for the (a, b) model for IBM Q0, although the spectrum is reproduced by definition, the value of E_C comes out much larger than the reference value of 300 MHz. The last line of the last row contains the parameters of an engineered model with reduced charge dispersion and increased anharmonicity (see Fig. S8 below). All parameters have been obtained by solving the HamPIEP (see Section II A 2).

Sample	Model	E_C/h	E_J/h	E_{J2}/E_J	E_{J3}/E_J	E_{J4}/E_J	I_c	a	b	c	d	$\bar{d}$	σ	Ω/h	G/h
KIT	standard	0.197	24.852	-	-	-	50.0	-	-	-	-	-	-	7.454	0.078
	E_{J2}	0.242	21.801	−0.019	-	-	44.0	-	-	-	-	-	-	7.454	0.086
	E_{J4}	0.242	21.997	−0.026	0.004	−0.001	43.9	-	-	-	-	-	-	7.454	0.086
	a, b	0.266	20.983	−0.042	0.006	−0.002	42.0	1.00	4.58	-	-	-	-	7.454	0.090
	$\bar{d}, \sigma$	0.293	20.186	−0.067	0.017	−0.007	40.1	-	-	-	-	1.06	0.45	7.454	−0.095
KIT-CD2	standard	0.206	22.704	-	-	-	45.7	-	-	-	-	-	-	7.454	0.086
	E_{J2}	0.242	20.530	−0.016	-	-	41.4	-	-	-	-	-	-	7.454	0.093
	E_{J4}	0.242	20.383	−0.010	−0.003	0.001	41.5	-	-	-	-	-	-	7.454	0.093
	a, b	0.280	19.337	−0.048	0.009	−0.002	38.6	1.52	3.16	-	-	-	-	7.454	0.100
	$\bar{d}, \sigma$	0.284	19.361	−0.057	0.014	−0.005	38.4	-	-	-	-	1.62	0.50	7.454	0.101
KIT-CD3	standard	0.223	13.803	-	-	-	27.8	-	-	-	-	-	-	7.454	0.080
	E_{J2}	0.242	13.164	−0.009	-	-	26.5	-	-	-	-	-	-	7.454	0.083
	E_{J4}	0.242	13.225	−0.013	0.002	−0.001	26.5	-	-	-	-	-	-	7.454	0.083
	a, b	0.247	13.075	−0.016	0.002	−0.000	26.2	4.07	1.96	-	-	-	-	7.454	0.084
	$\bar{d}, \sigma$	0.254	12.977	−0.024	0.005	−0.002	25.9	-	-	-	-	1.62	0.38	7.454	0.085
ENS	standard	0.167	23.191	-	-	-	46.7	-	-	-	-	-	-	7.739	0.179
	E_{J2}	0.181	22.053	−0.008	-	-	44.4	-	-	-	-	-	-	7.739	0.187
	E_{J4}	0.186	21.811	−0.014	0.001	−0.000	43.8	-	-	-	-	-	-	7.739	0.189
	a, b	0.186	21.835	−0.015	0.001	−0.000	43.9	2.21	3.81	-	-	-	-	7.739	0.189
	a, b, c, d	0.185	21.876	−0.013	0.001	−0.000	44.0	1.50	5.49	1.28	0.58	-	-	7.739	0.189
	$\bar{d}, \sigma$	0.195	21.505	−0.028	0.006	−0.002	42.9	-	-	-	-	1.63	0.39	7.739	0.194
MIT	standard	0.243	14.073	-	-	-	28.3	-	-	-	-	-	-	10.975	0.145
	E_{J2}	0.248	13.901	−0.003	-	-	28.0	-	-	-	-	-	-	10.975	0.146
	a, b	0.250	13.876	−0.004	0.000	−0.000	27.9	4.09	4.07	-	-	-	-	10.975	0.147
KIT2	standard	0.176	17.444	-	-	-	35.1	-	-	-	-	-	-	8.561	0.194
	a, b	0.176	17.443	−0.000	0.000	−0.000	35.1	8.27	12.47	-	-	-	-	8.561	0.194
TUM	standard	0.167	44.908	-	-	-	90.4	-	-	-	-	-	-	5.631	0.127
	E_{J2}	0.253	33.884	−0.031	-	-	68.7	-	-	-	-	-	-	5.631	0.156
	E_{J4}	0.337	29.023	−0.078	0.011	−0.001	59.5	-	-	-	-	-	-	5.631	0.180
	a, b	0.252	35.539	−0.057	0.011	−0.003	71.1	1.00	4.14	-	-	-	-	5.631	0.156
	a, b, c, d	0.360	28.262	−0.098	0.022	−0.007	57.5	1.00	4.03	1.94	−3.48	-	-	5.631	0.186
	$\bar{d}, \sigma$	0.261	34.675	−0.062	0.016	−0.006	68.8	-	-	-	-	1.61	0.54	5.628	0.152
Köln	standard	0.285	7.666–16.785	-	-	-	33.8	-	-	-	-	-	-	7.545	0.077
	E_{J4}	0.330	7.117–15.577	−0.023	0.004	−0.001	31.1	-	-	-	-	-	-	7.545	0.083
	$\bar{d}, \sigma$	0.331	7.144–15.627	−0.027	0.006	−0.002	31.1	-	-	-	-	1.93	0.43	7.545	0.083
IBM Q0	standard	0.302	11.925	-	-	-	24.0	-	-	-	-	-	-	7.160	0.133
	E_{J4}	0.300	14.672	−0.141	0.083	−0.027	34.7	-	-	-	-	-	-	7.160	0.133
	a, b	0.531	9.217	−0.118	0.039	−0.014	18.7	2.39	0.56	-	-	-	-	7.160	0.176
	engineered	0.300	15.049	−0.168	0.084	−0.008	37.0	-	-	-	-	-	-	7.159	0.136

be many different parameterizations that describe the observed spectra similarly well. Fundamentally, the distribution of transparencies for the conduction channels of a general Josephson junction can be very complicated. Therefore, it might not be possible to capture this complex many-body configuration in terms of only a few parameters, or in other words, reality does not necessarily fit into a simple mathematical model.

A selection of alternative models that we have considered for various experiments is shown in Fig. S4. In addition to the (a, b) model given by Eq. (S44), we show results for the (a, b, c, d) model given by Eq. (S41) for the experiments at KIT and ENS (since in these cases we have sufficient experimental data to determine all model parameters), as

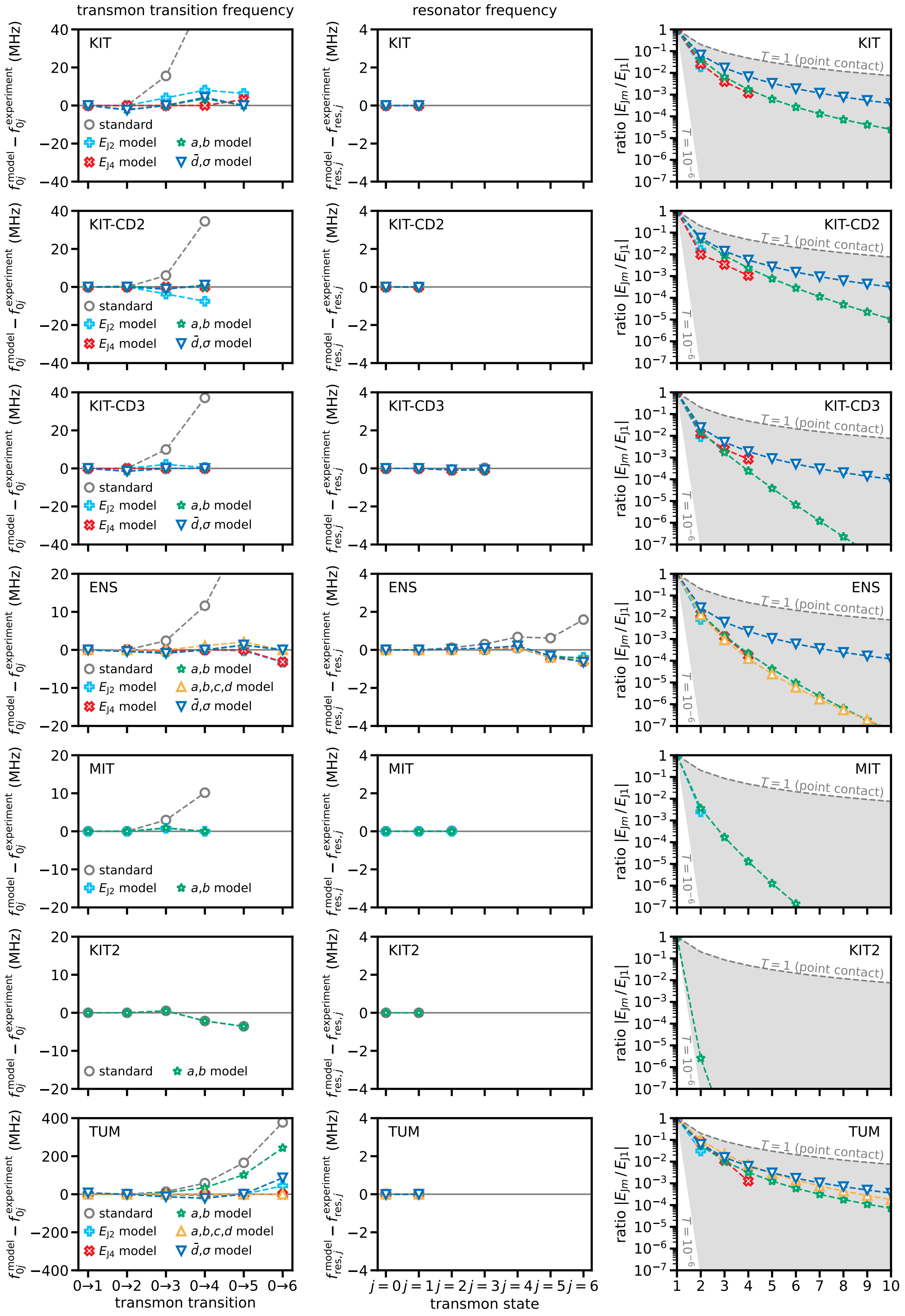

Figure S4. **Comparison of alternative models and further evidence.** Left column: Differences between the transmon transition frequencies $f_{0j}^{\rm model}$ predicted by the fitted models and $f_{0j}^{\rm experiment}$ for the transitions $0 \to j$. Middle column: Differences between the resonator frequencies $f_{{\rm res},j}^{\rm model}$ predicted by the fitted models and $f_{{\rm res},j}^{\rm experiment}$, conditional on the transmon being in state j. Right column: Absolute values of the Josephson energy ratios $|E_{{\rm J}m}/E_{\rm J}|$ obtained from the models. Each row shows a different experiment. Rows 1 through 4 show additional models (see text) for the KIT and ENS experiments discussed in the main text. The other rows show additional experiments with data extracted from the literature: Peterer et. al. [S35] (MIT), Schneider et. al. [S36, S37] (KIT2), Xie et. al. [S38, S39] (TUM). All models are identified by the same markers and colors across all panels (see legends). All model parameters are given in Table S3.

well as for the mesoscopic $(\bar{d}, \sigma)$ model described in Section I B 4. The values of all model parameters are given in Table S3. Note that all parameterized models have physical ratios $E_{\mathrm{J}m}/E_{\mathrm{J}}$ (i.e., alternating, decaying, and within the two limits). This is not automatically the case for the truncated $E_{\mathrm{J}2}$ and $E_{\mathrm{J}4}$ models for which the ratios are fitted independently (e.g. the KIT-CD2 case in Table S3).

As these results show, none of the models should be considered as the ultimate answer for the effects caused by the higher harmonics. It is an important task for future research to increase the accuracy in measuring fine-structure effects on the experimental side, and to find and study other alternative models and transparency distributions on the theoretical side, which could shed further light on understanding the complex structure of the Josephson effect in tunnel junctions.

2. *Additional evidence*

We find further support for our Josephson harmonics hypothesis by analyzing experiments with published spectroscopy results available in the literature, namely the experiments by Peterer et. al. [S35] (MIT), Schneider et. al. [S36] (KIT2; additional information is available in [S37]), and Xie et. al. [S38] (the spectroscopy is shown in Fig. 2.20 of [S39]). The results are shown in Fig. S4. In this figure, we also show evidence from the differences between predicted and measured resonator frequencies $f_{\mathrm{res},j}$ when the transmon is in state j (computed from the dispersive shifts for $j \geq 1$), as an addition to the evidence from the transmon transition frequencies shown in the main text (see Fig. 3). We see that a model including the higher-order Josephson harmonics can describe the data better than the standard transmon model. The only exception is KIT2, which is either an example for a highly uniform tunnel junction with very low transparencies or the data reported may not be accurate enough to reveal percent-level corrections in the Josephson harmonics.

3. *Exhaustive scan*

In this section, we give some additional details about the scanning procedure described in the Methods section. Furthermore, we show in Fig. S5 the detailed scanning results for all experiments considered in the main text, including the remaining transmons of the IBM Hanoi chip (cf. Fig. 3c in the main text). Additionally, we show three specific trajectories corresponding to the minimum, maximum, and geometric mean of $|E_{\mathrm{J}2}/E_{\mathrm{J}}|$.

Interestingly, when constraining $E_{\mathrm{J}5}$ and $E_{\mathrm{J}6}$ of some transmons to zero (see. e.g. IBM Q2), fitting only $E_{\mathrm{J}2}$ to $E_{\mathrm{J}4}$ yields unphysical results above the point-contact limit. These transmons have a very narrow range of allowed values for $E_{\mathrm{J}m}$, even for high orders such as $m = 6$. Furthermore, we see that when the bars for $|E_{\mathrm{J}N_f+1}/E_{\mathrm{J}}|$ do not extend down towards zero, truncation at $E_{\mathrm{J}N_f}$ is not possible to describe the spectrum. This is the case for the first two cooldowns of the KIT system (note however that it is indeed possible for KIT-CD3).

We note that by design, this procedure does not include non-perfect agreement with the experimental data. Taking into account experimental imprecision etc. would be a complementary study and it is to be expected that the allowed ranges for the $E_{\mathrm{J}m}/E_{\mathrm{J}}$ become slightly larger. Note, however, that the ranges can also be reduced by including additional information such as charge-dispersion measurements, as we discuss in the following section.

4. *Charge dispersion*

For both the standard transmon model and all trajectories of the harmonics model, we evaluate the charge dispersion

$$\delta f_{0j} = |f_{0j}(n_{\mathrm{g}} = 0) - f_{0j}(n_{\mathrm{g}} = 0.5)| \,, \tag{S45}$$

for the transmon transitions $0 \to j$. The results are shown in Fig. S6 for all transmons considered in the main text.

While the standard model can underestimate the charge dispersion (sometimes dramatically, see Fig. 4a of the main text), it is also possible that the charge dispersions are overestimated (see the green bars in Fig. S6). The latter can happen in the presence of high-transparency conduction channels. This agrees with the findings of [S40], where the charge dispersion was observed to decrease with increasing junction transparency, which happens because the height of the potential increases with transparency. However, we emphasize that when constraining both standard and harmonics models to describe the same measured frequencies, whether the charge dispersion actually decreases or increases (compared to the standard model) is a combination of where the transmon levels lie in the potential well and how the potential is affected by the Josephson harmonics (see the discussion after Eq. (S48)).

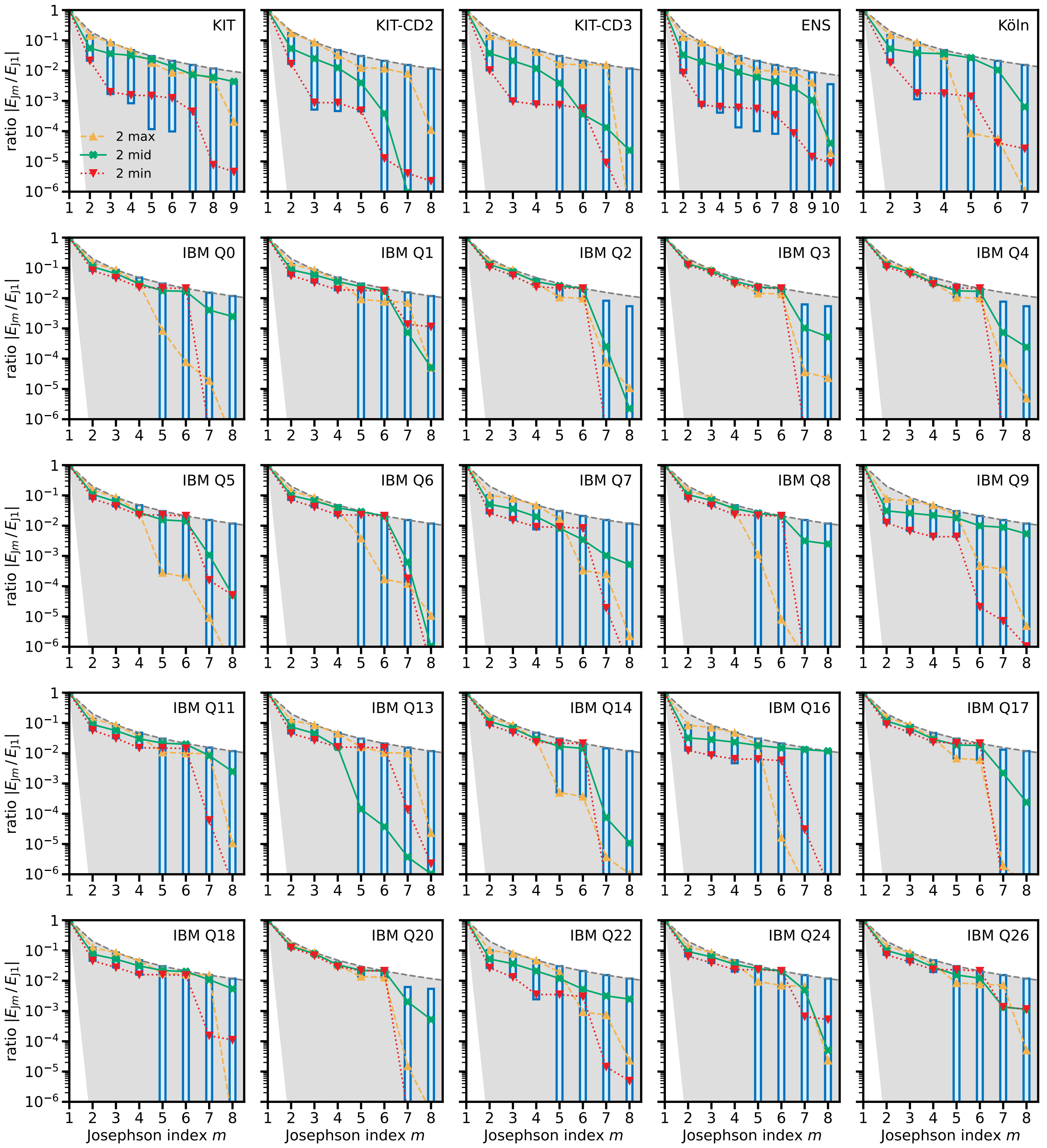

Figure S5. **Ranges of E_{Jm} coefficients that can reproduce the observed spectra.** The blue bars show the ranges of possible $|E_{Jm}/E_J|$, obtained by scanning four higher-order ratios $\mathbf{y}$ and for each $\mathbf{y}$ solving the HamPIEP (cf. Section II A 2) for the leading N_f ratios (see Methods). The lines represent three particular trajectories $\mathbf{e} = (1, E_{J2}/E_{J1}, \dots, E_{JN_f+4}/E_{J1})$ corresponding to the largest $|E_{J2}/E_J|$ (yellow upward-pointing triangles, "max"), the smallest $|E_{J2}/E_J|$ (red downward-pointing triangles, "min"), and the geometric mean of both (green crosses, "mid"). The magnitude of the corresponding $\sin(m\varphi)$ contribution to the current-phase relation $I_S(\varphi)$ is given by $m|E_{Jm}/E_J|$ (see Eq. (S13)). Each panel shows the result for the transmon indicated by the label in the top right corner. The ranges for the Köln transmon (top right panel) correspond to the same dataset considered in Fig. 3 of the main text (explicitly excluding further information about the charge dispersion, which would additionally reduce the extent of the bars, see Section I C 4).

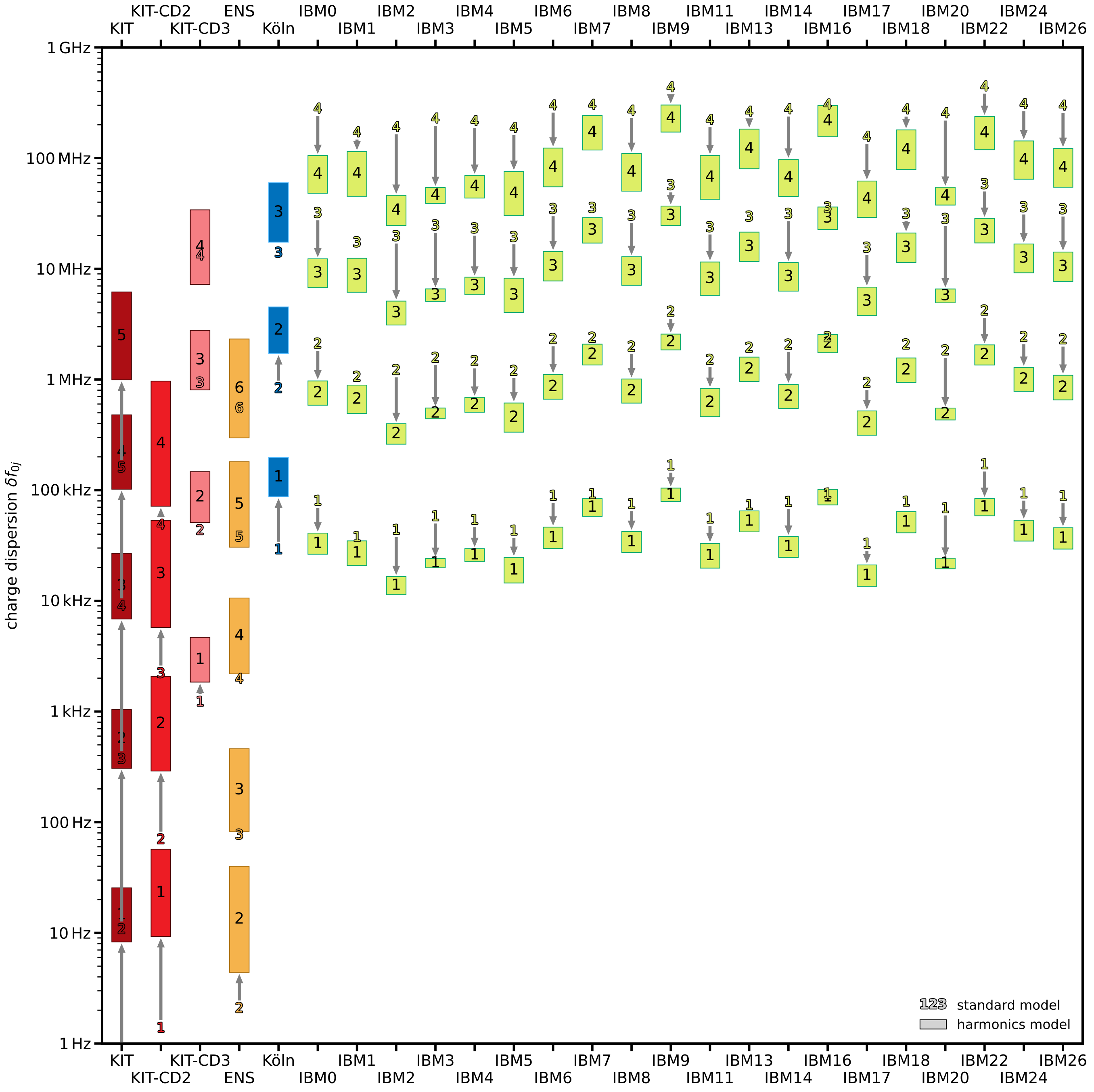

Figure S6. **Josephson harmonics can significantly increase or reduce the charge dispersion.** For each transmon considered in the main text, we use colored numbers as markers to represent the charge dispersions δf_{0j} predicted by the standard transmon model and filled bars with the corresponding level indices j to represent the ranges extracted from all trajectories of the harmonics model. Gray arrows are shown if there are significant corrections from the standard model prediction. The ranges for the Köln transmon correspond to the same dataset considered in Fig. 3 of the main text.

If additional measurements $\delta f_{0j}^{\text{experiment}}$ of the charge dispersion are available, this information can also be used to reduce the uncertainty of suitable Josephson harmonics models. One would then filter out, from all suitable trajectories $\mathbf{e} = (1, E_{J2}/E_{J1}, \ldots, E_{JN_f+4}/E_{J1})$, only those trajectories that agree with the measured charge dispersions. For the ENS sample, for instance, one can extract from Fig. 6b(6) of [S33] that the charge dispersion of level $j = 6$ is on the order of a few MHz. This is incompatible with the standard model (see the yellow "6" in Fig. S6) and would restrict

the parameter range in the harmonics model. The charge dispersion is thus a sensitive probe for deviations from the standard model (cf. also Fig. 4 in the main text for the Köln experiment).

We note that both the standard model and the harmonics model give an exponential *scaling* of the charge dispersion with E_J/E_C [S41]. For this reason, it is always possible to use the two parameters (E_C, E_J) of the standard model to fit either (i) f_{01} and f_{02} or (ii) f_{01} and δf_{01}. The latter case has been successfully demonstrated for qubit 2 in [S42]. For the Köln sample, one could analogously increase E_C (and decrease E_J) such that the gray dashed lines in Fig. 4a rise towards the measured data. However, in this case, the predictions for the higher level transitions $f_{02}, f_{03}, \ldots$ would be even further away from the measured frequencies.

5. *Hamiltonian*

In this section, we state the transmon-resonator Hamiltonian including the higher-order Josephson harmonics and discuss some properties of this Hamiltonian. The Hamiltonian of the transmon-resonator system is given by

$$H = 4E_C(n - n_g)^2 - \sum_{m\geq 1} E_{Jm} \cos(m\varphi) + \Omega a^\dagger a + Gn(a + a^\dagger)\,, \tag{S46}$$

where, in the charge basis $\{|n\rangle\}$, the operator $n = \sum_n n\,|n\rangle\langle n|$ is diagonal and the operator $\cos(m\varphi) = \sum_n 1/2\,(|n\rangle\langle n+m| + |n+m\rangle\langle n|)$ has constant entries on the mth subdiagonal (which represent correlated m-Cooper pair tunneling), $a^\dagger a$ is the number operator of the harmonic oscillator with $a = \sum_k \sqrt{k+1}\,|k\rangle\langle k+1|$ the bosonic annihilation operator given in the harmonic oscillator's eigenbasis. E_C denotes the transmon's charging energy, E_{Jm} the mth-harmonic Josephson energy, Ω is the resonator frequency and G denotes the transmon-resonator coupling strength.

The energy levels of H corresponding to the dressed transmon states $|\overline{0j}\rangle$ for $j = 0, 1, 2, \ldots$ together with the φ-dependent Josephson potentials

$$V(\varphi) = -\sum_{m\geq 1} E_{Jm} \cos(m\varphi) \tag{S47}$$

and the current-phase relations $I_S(\varphi)$ are shown in Fig. S7 for all samples. Note that the energy levels of all harmonics models are equal to all measured transitions and only differ for even higher levels. Depending on the height of the Josephson potentials, they can be either above or below the standard model.

We can compute the height of the potential (a.k.a. the depth of the potential well) as

$$V(\pi) - V(0) = 2(E_{J1} + E_{J3} + E_{J5} + \cdots)\,. \tag{S48}$$

Here we see that, compared to the height of the standard model $2E_J^{\text{std}}$, a large contribution of odd higher harmonics (which are all positive) can increase the height of the potential significantly.

This helps to understand why in Fig. 3a, the IBM transmons are the only devices for which most standard model predictions are lower than the measured transitions: Since the IBM transmons have the largest contributions from odd higher harmonics (cf. Fig. S5), the Josephson potentials are much deeper than expected from the standard model. Therefore, the energy levels of $j = 4$ (and often also $j = 3$) lie much higher than the standard model suggests (see Fig. S7.) Furthermore, the increase of the height of the IBM potentials due to strong odd harmonics in all trajectories explains the decrease of the charge dispersions, while for all other devices the dominating E_{J2} term (cf. Fig. S5) effectively flattens the potentials such that the charge dispersion increases (cf. Fig. S6).

6. *Perturbative expansion*

To obtain the leading-order Josephson harmonics corrections to the qubit frequency $\omega = 2\pi f_{01}$ and the anharmonicity $\alpha = 2\pi(f_{12} - f_{01})$, we expand the $\cos(m\varphi)$ terms in φ. We emphasize that this step, which is frequently done to obtain an anharmonic "Duffing" oscillator approximation of the transmon (see e.g. [S41, S46–S48]), is not suitable for an accurate study of higher transmon states (and neither the charge dispersion). We also note that this approximation is not applicable to all of the expressions for E_{Jm} obtained for certain transparency distributions in Section I B, as the double series

$$\sum_{m=1}^{\infty} \sum_{k=0}^{\infty} (-1)^k E_{Jm} \frac{(m\varphi)^{2k}}{(2k)!} \tag{S49}$$

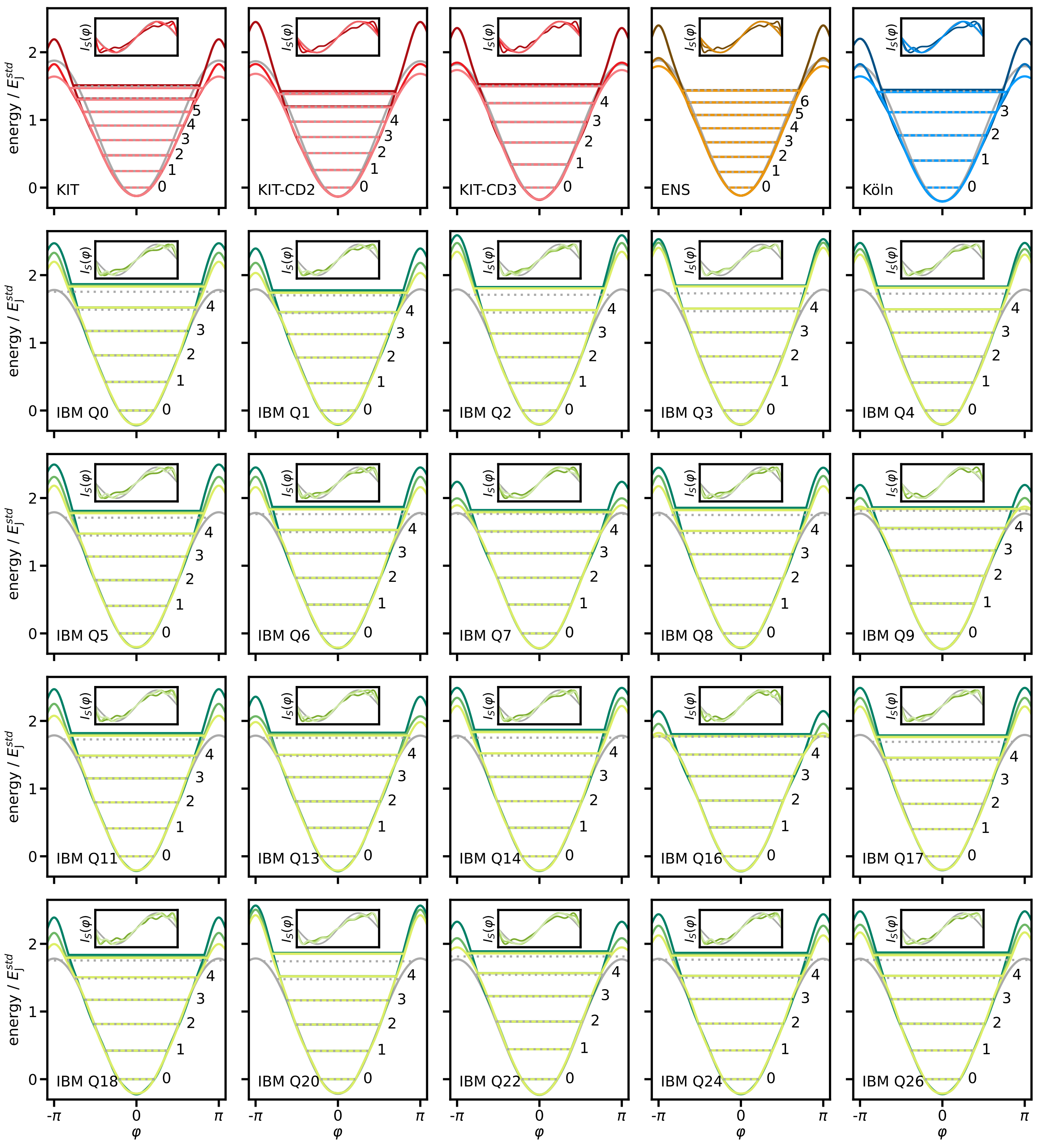

Figure S7. **Energy spectra and phenomenological current-phase relations for each sample considered in the main text.** The standard model is shown in gray. Three harmonics models given by the three trajectories “max”, “mid”, and “min” in Fig. S5 are shown in color (darker colors represent larger $|E_{J2}/E_J|$). The energy levels of the dressed transmon states $|\overline{0j}\rangle$ for $j = 0, 1, 2, \ldots$ are computed by diagonalizing the corresponding transmon-resonator Hamiltonian in Eq. (S46). Each potential $V(\varphi)$ is shifted such that the energy corresponding to $j = 0$ is at zero. Labels are shown for all measured transition frequencies. The insets show the respective current-phase relations $I_S(\varphi)$, in units of the critical current I_c obtained by maximizing over Eq. (S13) (cf. Table S3). We note that the more extreme deviations from the sinusoidal current-phase relations (darker lines) might be easily detectable in DC measurements [S43–S45]. The results for the Köln transmon correspond to the same dataset considered in Fig. 3 of the main text.

may not converge absolutely (as in the case of e.g. the point contact Eq. (S23)) and rearranging the terms as well as neglecting higher orders may lead to inconsistent results. The second-order expression of the Josephson contribution $H_\mathrm{J} = -\sum_m E_{\mathrm{J}m}\cos(m\varphi)$ to H is given by

$$H_\mathrm{J} \approx \left(\sum_{m=1}^{\infty} m^2 E_{\mathrm{J}m}\right)\frac{\varphi^2}{2}\,. \tag{S50}$$

Equation (S50) implies that the second-order correction to the standard model corresponds to the substitution rule

$$E_\mathrm{J} \to E_\mathrm{J}\left(1 + \sum_{m=2}^{\infty} m^2 \frac{E_{\mathrm{J}m}}{E_\mathrm{J}}\right)\,. \tag{S51}$$

Considering terms up to fourth order in φ (see [S46]), we find the analogous correction to the often-used approximation $\alpha \approx -E_\mathrm{C}$ (using units with $\hbar = 1$),

$$\alpha \approx -E_\mathrm{C}\frac{1+\sum_{m\geq 2} m^4 E_{\mathrm{J}m}/E_\mathrm{J}}{1+\sum_{m\geq 2} m^2 E_{\mathrm{J}m}/E_\mathrm{J}}\,. \tag{S52}$$

Similarly, we obtain for the qubit frequency,

$$\omega \approx \sqrt{8E_\mathrm{C}E_\mathrm{J}\left(1+\sum_{m=2}^{\infty} m^2\frac{E_{\mathrm{J}m}}{E_\mathrm{J}}\right)} + \alpha\,. \tag{S53}$$

Considering the expansion up to $E_{\mathrm{J}2}$ only yields

$$\omega \approx \sqrt{8E_\mathrm{C}E_\mathrm{J}\left(1+4\frac{E_{\mathrm{J}2}}{E_\mathrm{J}}\right)} + \alpha\,, \tag{S54}$$

$$\alpha \approx -E_\mathrm{C}\frac{1+16E_{\mathrm{J}2}/E_\mathrm{J}}{1+4E_{\mathrm{J}2}/E_\mathrm{J}}\,, \tag{S55}$$

for which we can make the following observation: Since $E_{\mathrm{J}2} < 0 < E_\mathrm{J}$ (see Eq. (S18)), it follows that $1+16E_{\mathrm{J}2}/E_\mathrm{J} < 1+4E_{\mathrm{J}2}/E_\mathrm{J} < 1$ and thus the fraction in Eq. (S55) is smaller than 1. This implies that, for the same measured values of α and ω, the value of E_C resulting from Eq. (S55) has to be larger than the one obtained from the analogous standard model relation $\alpha \approx -E_\mathrm{C}$. Similarly, the value of E_J resulting from Eq. (S54) has to be smaller than the one obtained from the analogous standard model relation $\omega \approx \sqrt{8E_\mathrm{C}E_\mathrm{J}} - \alpha$. Hence, it is reasonable to expect that the ratio $E_\mathrm{J}/E_\mathrm{C}$ is too large when using the standard model. This expectation is confirmed by several of the observed shifts of $E_\mathrm{J}/E_\mathrm{C}$ presented in Fig. 4c in the main text.

We caution the reader, however, that this argument relies on crude approximations and does not apply in general. For instance, if the ratios of Josephson energies do not decay fast enough and are relevant to high order, the value of $E_\mathrm{J}/E_\mathrm{C}$ computed from the standard model can actually be too small rather than too large (and the charge dispersion may be overestimated by the standard model). This is supported by the IBM data shown in Fig. 4c in the main text.

7. *Engineering $E_{\mathrm{J}m}$ coefficients*

As discussed in the main text (see Fig. 4b), the IBM transmons give evidence that one can use strong contribution from higher harmonics $E_{\mathrm{J}2}, E_{\mathrm{J}3}, E_{\mathrm{J}4}$ (cf. Fig. S5) to engineer devices with a reduced charge dispersion δf while keeping the anharmonicity $|\alpha| = 2\pi|f_{12} - f_{01}|$ constant. A natural question is now whether one can also increase the anharmonicity (e.g. to mitigate leakage [S49–S51]) by further engineering the $E_{\mathrm{J}m}$ contributions. We note that in practice it is not straightforward to tune the values of coefficients $(E_{\mathrm{J}2}, E_{\mathrm{J}3}, E_{\mathrm{J}4})$ to arbitrary values. To achieve this, one probably requires a combination of various strategies, such as shaping the channels' transparencies, adding inductive elements in series, flux bias, etc. (see also [S52]).

In Fig. S8a, we show that there exist sets of $E_{\mathrm{J}m}$ coefficients which give a reduced charge dispersion and increased anharmonicity (yellow arrow). The corresponding Josephson energy ratios are given in Fig. S8b. We see that the main reason for the increased anharmonicity is a reduction of $E_{\mathrm{J}4}$, while $E_{\mathrm{J}2}$ and $E_{\mathrm{J}3}$ stay roughly the same. This makes sense as $E_{\mathrm{J}3}$ must stay large to maintain the height of the potential for the reduced charge dispersion (see Eq. (S48)). The reduced $E_{\mathrm{J}4}$ then makes the potential slightly wider around level $|2\rangle$, which draws the level towards the bottom of the potential well and thus increases the anharmonicity (see Fig. S8c). We emphasize that whether it is possible or not to engineer such a set of $E_{\mathrm{J}m}$ coefficients in a real device is currently an open question.

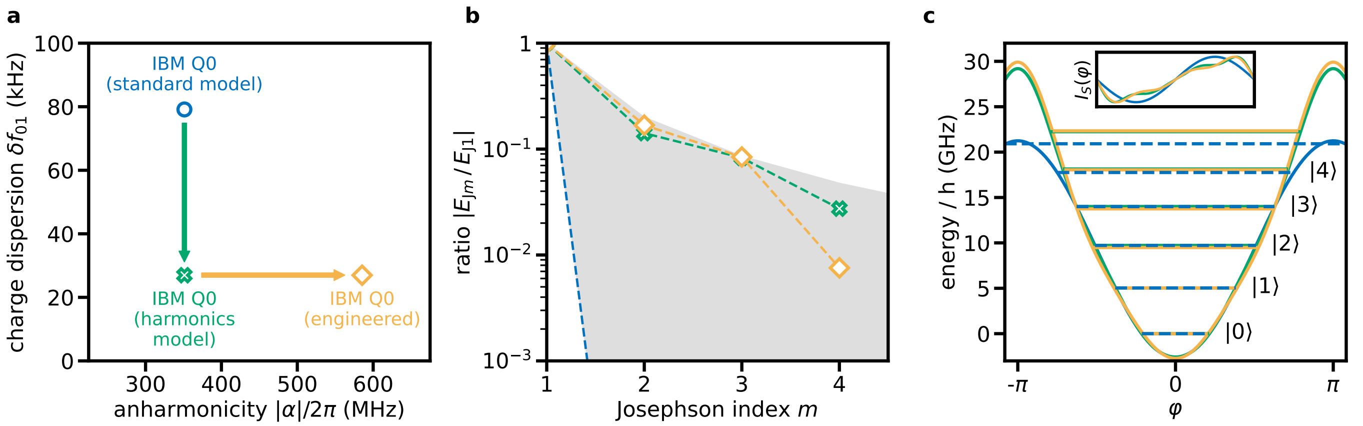

Figure S8. **Engineering $E_{\mathrm{J}m}$ coefficients can both reduce charge dispersion and increase anharmonicity.** **a** Map of the charge dispersion δf_{01} and the absolute anharmonicity $|\alpha|$ for the standard model for IBM Q0 (blue circle), the $E_{\mathrm{J}4}$ harmonics model (green cross), and an engineered $E_{\mathrm{J}4}$ model with increased anharmonicity (yellow diamond). The model parameters for the latter were chosen such that the qubit frequency f_{01}, the resonator frequency $f_{\mathrm{res},0}$, and the charge dispersion δf_{01} stay constant (they are listed in the last row of Table S3). **b** The corresponding Josephson energy ratios $|E_{\mathrm{J}m}/E_{\mathrm{J}1}|$, using the same coloring. **c** The corresponding potentials and current-phase relations (cf. Fig. S7), using the same coloring.

D. Alternative corrections

After observing that the standard transmon model cannot describe the measured transition frequencies, it might seem natural to consider alternative possible modifications to the model, such as an additional stray inductance present in the circuit, hidden electromagnetic modes, the coupling to other qubits as present on the IBM device, or an asymmetry in the superconducting electrodes. Although the theoretical discussion given above suggests that higher harmonics are the *expected* correction to describe the measurements, from a phenomenological perspective, such alternative modifications should not be ruled out a priori.

For this reason, we here discuss alternative corrections to the model, and why we have come to the conclusion that these alternatives do not provide the same universal quality to solve the disagreement between standard model and experiment for the considered samples. It would be an interesting direction of research to study how the inclusion of Josephson harmonics would influence advanced circuit quantization and measurement techniques [S48, S53–S60].

1. *Series inductance*

In this section, we show that a small linear stray inductance L in series with the JJ also induces higher harmonic corrections. In a transmon qubit, such a linear stray inductance can arise from the leads that connect the Josephson junction to the shunt capacitance. In an equivalent circuit diagram, this inductance would appear in series with the Josephson junction as shown in Fig. S9a.

To get a feeling for the size of L, we consider the KIT system as an example (cf. Table S3 and Section III A). From electromagnetic simulation (see Fig. S18e), we obtain a value for a linear stray inductance of $L \approx 0.380\,\mathrm{nH}$. In terms of energies, using the relation $E_\mathrm{L} = (\Phi_0/2\pi)^2/L$ with the magnetic flux quantum Φ_0, we have $E_\mathrm{L}/h \approx 430\,\mathrm{GHz}$, so $E_\mathrm{J}/E_\mathrm{L} \approx 0.058$. The characteristic energy ratio $E_\mathrm{J}/E_\mathrm{L}$ is also called the *screening parameter* in SQUID terminology [S61].

The circuit in Fig. S9a is described by the Lagrangian

$$\mathcal{L} = \frac{1}{2}C(\dot{\Phi}_\mathrm{J} + \dot{\Phi}_\mathrm{L})^2 - \frac{1}{2L}\Phi_\mathrm{L}^2 + E_\mathrm{J}\cos\varphi_\mathrm{J}\,, \tag{S56}$$

where Φ_J (Φ_L) is the flux across the junction (inductance). Furthermore, we define $\varphi_\mathrm{J} \equiv 2\pi\Phi_\mathrm{J}/\Phi_0$, and we implicitly assume this relation between all lower-case (φ) and upper-case (Φ) flux variables in what follows. We perform a change of variables $\varphi = \varphi_\mathrm{J} + \varphi_\mathrm{L}$ and $\varphi_\Delta = \varphi_\mathrm{J} - \varphi_\mathrm{L}$. Then, as $\dot{\varphi}_\Delta$ does not occur in the Lagrangian, we have $\mathrm{d}/\mathrm{d}t\,(\partial\mathcal{L}/\partial\dot{\varphi}_\Delta) = 0$ and thus $\partial\mathcal{L}/\partial\varphi_\Delta = 0$. This yields an equation that we can use to eliminate φ_Δ, which is justified if $E_\mathrm{L}/E_\mathrm{C} \gg 1$ and the stray capacitance in parallel to the JJ is much smaller than C (roughly 1 fF vs. 80 fF for the

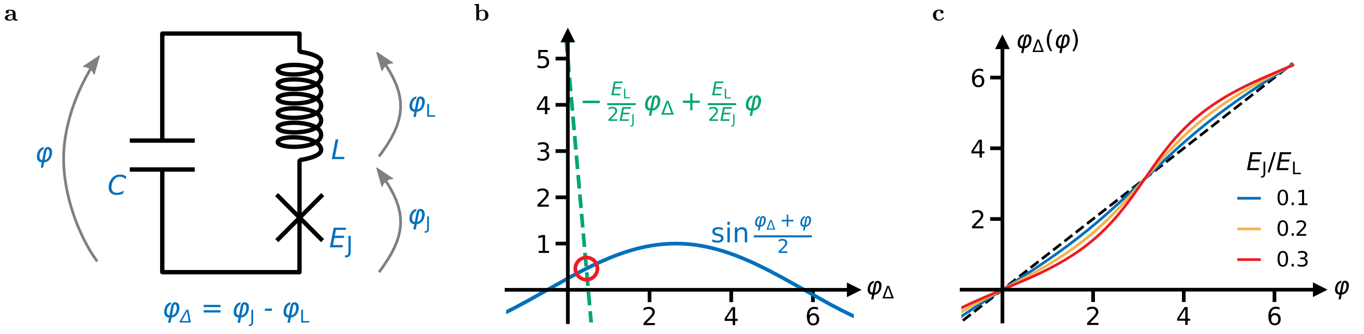

Figure S9. **Circuit quantization with an additional linear stray inductance in series.** **a** Lumped-element circuit diagram of a JJ with an additional linear stray inductance. The transmon is described in terms of the total capacitance C, the Josephson energy E_J characterizing the non-linear Josephson inductance, and an additional finite stray inductance L, in series with the JJ. **b** The solution of Eq. (S57) is determined as the intersection of a sine function (blue) and a straight line (green). Since the largest slope of the sine function is $\max|\partial/\partial\varphi_\Delta \sin((\varphi_\Delta+\varphi)/2)| = 1/2$, this intersection is unique if $E_\mathrm{L}/2E_\mathrm{J} > 1/2$, i.e., $E_\mathrm{J}/E_\mathrm{L} < 1$. The example solution (red circle) corresponds to $\varphi_\Delta(\varphi = 0.5) \approx 0.45$ for $E_\mathrm{J}/E_\mathrm{L} = 0.05$. **c** Characteristic form of the solution $\varphi_\Delta(\varphi)$ of Eq. (S57) for several values of $E_\mathrm{J}/E_\mathrm{L}$ (see legend). $\varphi_\Delta(\varphi)$ is an odd function with a 2π translation invariance that oscillates closely around φ (dashed diagonal).

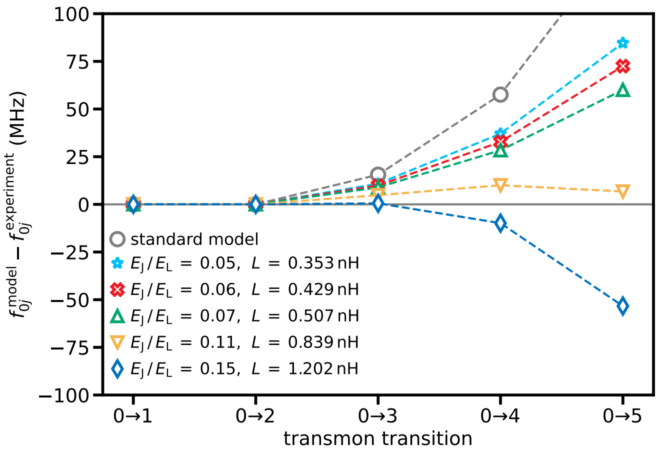

Figure S10. **Harmonics from additional series inductance alone cannot correct the standard transmon model.** Markers show the difference between the frequency f_{0j}^model predicted by the Hamiltonian Eq. (S61) (including the resonator H_res from Eq. (3) in the main text) and the measured transition frequency $f_{0j}^\mathrm{experiment}$ from $|0\rangle$ into a higher transmon state $|j\rangle$ for the KIT experiment. Different colors represent different values of $E_\mathrm{J}/E_\mathrm{L}$, corresponding to a different series inductance L (see legend). In particular, the result for the KIT inductance extracted from electromagnetic simulation, $L = 0.380\,\mathrm{nH}$ (cf. Fig. S18) corresponding to $E_\mathrm{J}/E_\mathrm{L} \approx 0.058$, would fall between the blue stars and the red crosses; including the contribution from kinetic inductance would increase L to about 0.5 nH (cf. Section III A), corresponding approximately to the upward pointing green triangles. Gray circles denote the standard transmon model, corresponding to the limit $L = 0$. For each case, the given $E_\mathrm{J}/E_\mathrm{L}$ only fixes the ratios $E_{\mathrm{J}m}/E_\mathrm{L}$ (cf. Table S4), and the four standard model parameters $(E_\mathrm{C}, E_\mathrm{J}, \Omega, G)$ are adjusted slightly such that the first two transmon transition frequencies and the first two resonator frequencies match the observed values, in order to make the cases easily comparable. Dashed lines are guides to the eye.

KIT sample) such that the Born-Oppenheimer approximation is applicable (see [S56, S62] for more information). The equation reads

$$\sin\frac{\varphi_\Delta+\varphi}{2} = -\frac{E_\mathrm{L}}{2E_\mathrm{J}}\varphi_\Delta + \frac{E_\mathrm{L}}{2E_\mathrm{J}}\varphi\,, \tag{S57}$$

and is related to Kepler's transcendental equation [S62–S64]. For $0 < E_\mathrm{J} < E_\mathrm{L}$, Eq. (S57) has a unique solution for φ_Δ, defined by the intersection of a sine function and a straight line (see Fig. S9b). Although we cannot solve for φ_Δ analytically, Eq. (S57) uniquely determines φ_Δ as a function of φ, and we write $\varphi_\Delta(\varphi)$ to denote this solution. It is shown in Fig. S9c for several values of $E_\mathrm{J}/E_\mathrm{L}$.

The function $\varphi_\Delta(\varphi)$ has two symmetry properties that can be extracted from Eq. (S57). The first is that it is odd, which can be shown by replacing $\varphi \mapsto -\varphi$ in Eq. (S57). Using the symmetry of the sine function, we obtain the same equation with $\varphi_\Delta \mapsto -\varphi_\Delta$, and since Eq. (S57) is the uniquely defining equation for $\varphi_\Delta(\varphi)$, we have $\varphi_\Delta(-\varphi) = -\varphi_\Delta(\varphi)$. The second symmetry property of $\varphi_\Delta(\varphi)$ is a 2π translation invariance, which can be shown in the same manner, yielding $\varphi_\Delta(\varphi+2\pi) = \varphi_\Delta(\varphi) + 2\pi$.

E_J/E_L	E_{J1}/E_J	E_{J2}/E_J	E_{J3}/E_J	E_{J4}/E_J
0.01	0.99999	−0.00250	0.00001	−0.00000
0.05	0.99969	−0.01249	0.00031	−0.00001
0.07	0.99939	−0.01747	0.00061	−0.00003
0.1	0.99875	−0.02492	0.00124	−0.00008
0.2	0.99501	−0.04934	0.00489	−0.00065
0.5	0.96907	−0.11490	0.02710	−0.00850
	0.96908	−0.11491	0.02686	−0.00833

Table S4. Values of E_{Jm}/E_J for a linear stray inductance in series with the JJ (cf. Fig. S9a), for different values of E_J/E_L (i.e. the screening parameter [S61]). Small values of E_J/E_L correspond to a small series inductance L. The ratios have been computed from Eq. (S62) using $K = 10000$ (which gives accurate results up to machine precision). Gray entries in a separate row correspond to the approximations in Eq. (S64) and are only given if the approximation is not equal to the numerically exact result within five decimal digits.

Given $\varphi_\Delta(\varphi)$, we perform the Legendre transformation of Eq. (S56) to obtain the Hamiltonian

$$H = 4E_C n^2 - E_J \cos\frac{\varphi + \varphi_\Delta(\varphi)}{2} + \frac{E_L}{2}\left(\frac{\varphi - \varphi_\Delta(\varphi)}{2}\right)^2 . \tag{S58}$$

Note that the series inductance does *not* break the 2π periodicity of the Hamiltonian, despite the occurrence of φ^2. This is in contrast to a shunt inductance, for which the flux φ and the charge n would need to be treated as non-compact operators with continuous spectrum $\mathbb{R}$, see [S65–S68]. For a series inductance, though, due to the two symmetry properties of $\varphi_\Delta(\varphi)$, the two φ-dependent terms of Eq. (S58) are even and 2π-periodic. Therefore, we can write the two φ-dependent terms of Eq. (S58) as Fourier cosine series,

$$\cos\frac{\varphi + \varphi_\Delta(\varphi)}{2} = \sum_m c_m \cos(m\varphi)\,, \tag{S59a}$$

$$\left(\frac{\varphi - \varphi_\Delta(\varphi)}{2}\right)^2 = \sum_m s_m \cos(m\varphi)\,. \tag{S59b}$$

The coefficients c_m and s_m can be obtained numerically by (i) solving Eq. (S57) for $\varphi_\Delta(\varphi_k)$, where $\varphi_k = \pi(k+1/2)/K$ with $k = 0, \ldots, K-1$ and $K \gg 1$ controls the accuracy, and (ii) using the discrete cosine transform (DCT). To see this, we note that any even 2π-periodic function $g(\varphi)$ can be written as a Fourier cosine series $g(\varphi) = \sum_m g_m \cos(m\varphi)$, where

$$g_m = \frac{2}{\pi}\int_0^\pi g(\varphi)\cos(m\varphi)\,\mathrm{d}\varphi \approx \frac{2}{\pi}\sum_{k=0}^{K-1} g(\varphi_k)\cos(m\varphi_k)\frac{\pi}{K} = \frac{1}{K}\mathrm{DCT}_m(g) \tag{S60}$$

for $m \geq 1$ and $\mathrm{DCT}_m(g) = 2\sum_k g(\varphi_k)\cos(m\varphi_k)$ denotes the type-II DCT, and $g_0 = 1/\pi\int_0^\pi g(\varphi)\,\mathrm{d}\varphi \approx \sum_{k=0}^{K-1} g(\varphi_k)$. Neglecting the constants c_0 and s_0, we obtain the Hamiltonian

$$H = 4E_C n^2 - \sum_{m\geq 1} E_{Jm}\cos(m\varphi)\,, \tag{S61}$$

with the higher harmonic contributions given by

$$E_{Jm} = E_J\left(c_m - \frac{E_L}{2E_J}s_m\right) . \tag{S62}$$

The ratios E_{Jm}/E_J are shown in Table S4 for several values of E_J/E_L. Note that here, we typically have $E_{J1} \neq E_J$, since E_J characterizes the JJ while E_{J1} includes the series inductance as a separate circuit element. We remark that the ratios alternate in sign and decay in magnitude with increasing order m, similar to the Josephson harmonics arising from the conduction channel transparencies (cf. Section I B).

It is possible to obtain closed-form approximations for the leading-order ratios E_{Jm}/E_J by expanding the small quantity in Eq. (S57), $(\varphi - \varphi_\Delta(\varphi))/2$, in powers of E_J/E_L. To this end, we rewrite Eq. (S57) as

$$\frac{\varphi - \varphi_\Delta(\varphi)}{2} = \frac{E_J}{E_L}\left(\sin\varphi\cos\frac{\varphi - \varphi_\Delta(\varphi)}{2} - \cos\varphi\sin\frac{\varphi - \varphi_\Delta(\varphi)}{2}\right) . \tag{S63}$$

After substituting $(\varphi - \varphi_\Delta(\varphi))/2 = \sum_{k\geq 1} a_k (E_\mathrm{J}/E_\mathrm{L})^k$ and expanding the sine and cosine functions, we iteratively obtain $a_1, \ldots, a_5$ by comparing coefficients. Performing the same expansion in the Hamiltonian Eq. (S58) and inserting the expression for $a_1, \ldots, a_5$ yields the fifth-order approximations

$$\frac{E_\mathrm{J1}}{E_\mathrm{J}} \simeq 1 - \frac{1}{8}\left(\frac{E_\mathrm{J}}{E_\mathrm{L}}\right)^2 + \frac{1}{192}\left(\frac{E_\mathrm{J}}{E_\mathrm{L}}\right)^4 , \tag{S64a}$$

$$\frac{E_\mathrm{J2}}{E_\mathrm{J}} \simeq -\frac{1}{4}\left(\frac{E_\mathrm{J}}{E_\mathrm{L}}\right) + \frac{1}{12}\left(\frac{E_\mathrm{J}}{E_\mathrm{L}}\right)^3 - \frac{1}{96}\left(\frac{E_\mathrm{J}}{E_\mathrm{L}}\right)^5 , \tag{S64b}$$

$$\frac{E_\mathrm{J3}}{E_\mathrm{J}} \simeq \frac{1}{8}\left(\frac{E_\mathrm{J}}{E_\mathrm{L}}\right)^2 - \frac{9}{128}\left(\frac{E_\mathrm{J}}{E_\mathrm{L}}\right)^4 , \tag{S64c}$$

$$\frac{E_\mathrm{J4}}{E_\mathrm{J}} \simeq -\frac{1}{12}\left(\frac{E_\mathrm{J}}{E_\mathrm{L}}\right)^3 + \frac{1}{15}\left(\frac{E_\mathrm{J}}{E_\mathrm{L}}\right)^5 . \tag{S64d}$$

As Table S4 shows, these expressions are a good approximation to the numerically exact result for the leading-order harmonics if the series inductance and thus $E_\mathrm{J}/E_\mathrm{L}$ is not too large.

Finally, to see whether the higher harmonics from the linear stray inductance can explain the deviations between computed and measured spectra (see Fig. 2a in the main text), we perform the same test when using H in Eq. (S62) as a model. The result is shown in Fig. S10 for the KIT system. However, the correction corresponding to $L = 0.380\,\mathrm{nH}$ ($L = 0.5\,\mathrm{nH}$) as extracted from electromagnetic simulation, between the blue stars and the red crosses (including kinetic inductance effects, green triangles) shows a similar deviation from the experiment as the standard transmon model. Also, when the inductance L is increased so much that the next transmon transition $0 \rightarrow 3$ matches the experiment (blue diamonds), the deviation for higher transmon transitions systematically bends into the other direction. Thus we conclude that, although the linear stray inductance does result in an appreciable correction (and it is probably part of the ranges shown in Fig. 3c in the main text and Fig. S5), it is not the main solution to revise the standard transmon model. We note that it would be interesting to work out the consequences of a series inductance on circuits with multiple JJs.

2. *Additional hidden modes*

The Hamiltonian in Eq. (S46) includes only a single-mode resonator contribution, namely the readout resonator with bare frequency Ω and coupling strength G. However, the electromagnetic environment of the device may contain additional modes. A valid hypothesis for a correction to the Hamiltonian is therefore the existence of spurious, hidden electromagnetic modes (so-called "dark" modes) that couple weakly to the transmon.

For this reason, we consider the addition of a second hidden mode to the Hamiltonian,

$$H' = H + \Omega_\mathrm{dark} b^\dagger b + G_\mathrm{dark} n(b + b^\dagger) , \tag{S65}$$

where $b^\dagger$ (b) are the hidden mode's bosonic creation (annihilation) operators, Ω_dark is the frequency, G_dark is the coupling strength to the transmon, and H is given by Eq. (3). We consider a coupling $G_\mathrm{dark}/h = 0.009\,\mathrm{GHz}$ that is ten times weaker than the coupling G between the transmon and the resonator in the standard model (see Table S3).

We study the KIT system as an example. The effect of additional modes is shown in Fig. S11 as a function of Ω_dark. Around certain frequencies of Ω_dark, the transmon transition frequencies can deviate a lot from the predicted frequency when no hidden modes are considered. Thus we see that additional modes can indeed have a strong effect on the spectrum, despite the weak coupling strength. However, Fig. S11 also shows that there is no single frequency Ω_dark that can bring all model frequencies (solid lines) to the experimental values (dotted lines).

In Fig. S12, we show the difference between model and experiment in the same way as in Fig. 3 of the main text, for the particular dark mode frequencies indicated in Fig. S11. These results show that additional hidden modes do not correct for the systematic deviation of the higher states, i.e., the curvature in the differences between the standard model prediction and the experimental values is still there if hidden modes are included.

Finally, we remark that no evidence for spurious hidden modes has been found in a 2D coplanar waveguide system experiment [S69]. Also for the KIT sample, we have not seen any evidence for such dark modes from T_2 measurements. Therefore, we do not consider the addition of spurious modes as the solution to the mismatch between the standard model and the experimental data.

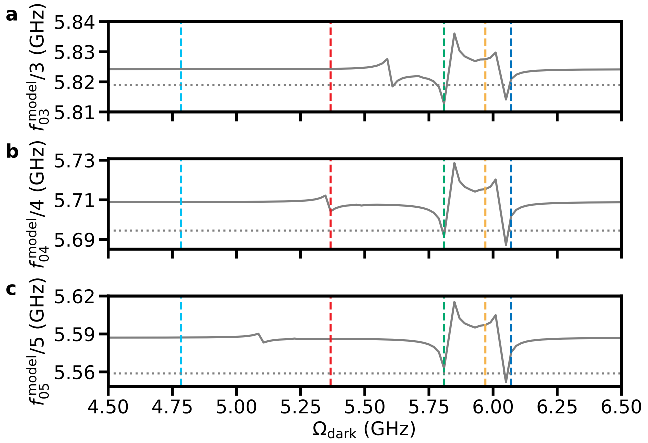

Figure S11. **Additional hidden modes coupling weakly to the qubit can affect the spectrum.** Transition frequencies f_{0j}^{model}/j as a function of the hidden mode frequency Ω_{dark} for **a** $j=3$, **b** $j=4$ and **c** $j=5$ (gray lines). The kinks in the gray lines indicate singularities (which are not fully resolved by the spacing of the scanned Ω_{dark} and thus connected by a straight line) when the dark mode's frequency Ω_{dark} is close to particular transition frequencies in the spectrum such as $f_{01} = 6.039\,\text{GHz}$. Dashed vertical lines correspond to the specific dark modes considered in Fig. S12 using the same colors. In each panel, a dotted horizontal line indicates the measured frequencies $f_{0j}^{\text{experiment}}/j$. In particular, there is no single Ω_{dark} for which each model frequency (solid line) matches the measured frequency (dotted line).

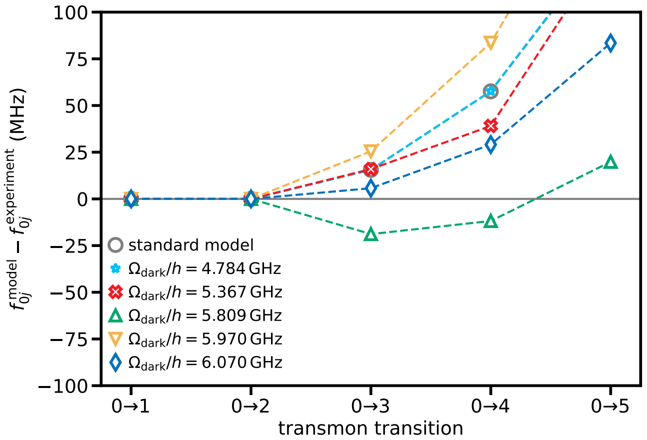

Figure S12. **Additional hidden modes cannot easily rescue the standard transmon model.** Markers show the difference between the frequency f_{0j}^{model} predicted by the Hamiltonian Eq. (S65) and the measured transition frequency $f_{0j}^{\text{experiment}}$ from $|0\rangle$ into a higher transmon state $|j\rangle$ for the KIT experiment. Gray circles denote the standard model from the main text without any dark modes. Different colors represent different values of Ω_{dark} (see Fig. S11 and legend). For each case, the four standard model parameters $(E_{\text{C}}, E_{\text{J}}, \Omega, G)$ are adjusted slightly such that the first two transmon transition frequencies and the first two resonator frequencies match the observed values, in order to make the cases easily comparable. Dashed lines are guides to the eye.

3. *Multi-qubit coupling*

Since the IBM Hanoi device is a multi-qubit device, an obvious question is whether the coupling between the qubits on the chip has an effect on the transmon transition frequencies, and whether this effect may provide an alternative path to rescue the standard transmon model. Here we show that for the IBM Hanoi device, this effect is much too small to significantly shift the transmon frequencies.

The transmons on the IBM Hanoi device are coupled by very short coplanar waveguide resonators (cf. [S70]). Due to the small size of the resonators, their frequencies are much larger than the transmon frequencies, so the coupling can be treated as capacitive transmon-transmon coupling (cf. [S71]). The coupled Hamiltonian takes the form

$$H = \sum_i \Big(4E_{\text{C}_i}(n_i - n_{gi})^2 - E_{\text{J}_i}\cos\varphi_i + \Omega_i a_i^\dagger a_i + G_i n_i (a_i + a_i^\dagger)\Big) + \sum_{\langle i,j\rangle} J n_i n_j\,, \tag{S66}$$

where the first term represents the standard model Hamiltonians of each transmon i including its readout resonator, and J is the capacitive coupling strength between all connected transmon pairs $\langle i,j\rangle$. The particular connectivity of the IBM Hanoi device is shown in Fig. S13a.

To examine whether the coupling can explain the deviations in the spectra shown in Fig. 3a of the main text, we diagonalize H for the joint system of up to three neighboring transmons and their respective readout resonators. In Fig. S13b–e, we show the absolute difference $|f_{0j}^{\text{coupled}} - f_{0j}|$ between the coupled and the uncoupled systems as a function of the transmon-transmon coupling strength J/h. Shown are only the qubits that could be measured up to level $|4\rangle$ and that are also coupled to a qubit that could be measured. We scan the coupling strength J/h from 0 to 50 MHz. Most experiments have $J/h \lesssim 5\,\text{MHz}$ although experiments beyond $J/h \approx 50\,\text{MHz}$ are possible (see [S71]). At this end of the scale, the coupling can have a significant impact on the spectrum, in the sense that deviations go up to 100 MHz (see Fig. S13b). However, the transmon qubits of the IBM Hanoi device are around $J/h \approx 2\,\text{MHz}$ by design (see the arrows in Fig. S13b–e). For values of J/h around 2 MHz, the effect of the coupling on the spectrum is very small and cannot correct for the observed deviations from the experimental data. For this reason, we conclude that also the coupling to the other qubits cannot explain the failure of the standard transmon model shown in Fig. 3a of the main text.

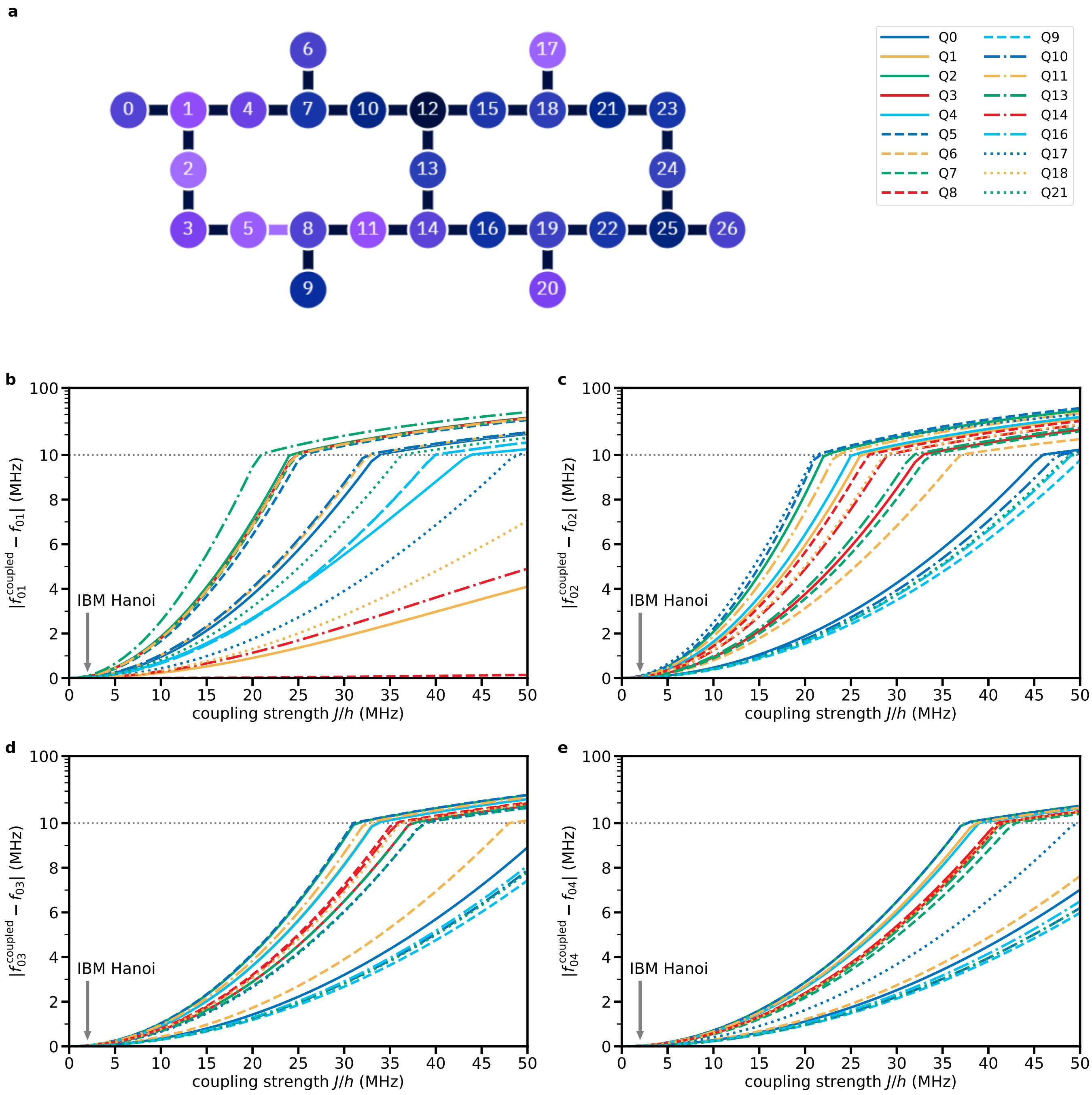

Figure S13. **Effects of the capacitive transmon-transmon coupling on the spectrum are negligible for the IBM multi-qubit device.** **a** The connectivity between the transmons on the IBM Hanoi device. The color of each qubit represents the qubit frequency from $f_{01} = 4.7190\,\mathrm{GHz}$ (dark blue, Q12) to $f_{01} = 5.2562\,\mathrm{GHz}$ (light purple, Q2). **b**–**e** Absolute difference of the frequencies $f_{0j}^{\mathrm{coupled}}$ obtained from the diagonalization of the capacitively coupled multi-transmon system (including up to 3 transmons and their respective readout resonators) and the frequencies f_{0j} obtained from the diagonalization of the single-transmon-resonator system for the **b** $0 \rightarrow 1$ transition **c** $0 \rightarrow 2$ transition **d** $0 \rightarrow 3$ transition and **e** $0 \rightarrow 4$ transition. The gray arrow indicates the design value of $2\,\mathrm{MHz}$ for the IBM Hanoi device. Although the transmon-transmon coupling can have a strong effect on the spectrum for large values of the coupling strength J (note the change from linear to log-scale indicated by the dotted gray line), for values around $2\,\mathrm{MHz}$, the effects on the spectrum are negligible with less than $1\,\mathrm{MHz}$ deviation.

4. Asymmetry in the superconducting gaps

In tunnel junctions with aluminum leads, the dependence of the superconducting gap on film thickness can cause a slight difference in the gaps of the two electrodes, $\Delta_1 \neq \Delta_2$, because in general the top layer has to be thicker than the bottom layer for fabrication reasons. This difference has recently been shown to play a role in the context of quasiparticle tunneling [S13, S72]. In this section, we estimate the influence of gap asymmetry on the ratio between second and first Josephson harmonics. We show that the effect is negligible at typical gap asymmetries of around 10 %, and even the presence of larger gap asymmetries would only slightly suppress the effect of higher harmonics.

We start from Zaitsev's treatment of asymmetric S_1cS_2 junctions [S73], in which the single-channel current-phase relation is expressed as

$$I_S(\varphi) \propto \sum_{\omega>0} \left\langle \frac{T(\alpha) f_1 f_2 \sin\varphi}{2 + T(\alpha)(g_1 g_2 + f_1 f_2 \cos\varphi - 1)} \right\rangle_\alpha , \tag{S67}$$

where the angular brackets average over an angle-dependent transparency $T(\alpha)$, the sum is over Matsubara frequencies $\omega > 0$, and $f_i = \Delta_i/\sqrt{\omega^2 + \Delta_i^2}$ and $g_i = \omega/\sqrt{\omega^2 + \Delta_i^2}$ depend on the two gaps. We consider the zero-temperature limit in which the sum over ω becomes an integral and assume the transparency to be independent of α; then for equal gaps $\Delta_i \equiv \Delta$ we recover Eq. (2) of the main text.

For generic values of the gaps, keeping the first two terms in the Fourier expansion of $I_S(\varphi)$ we find

$$I_S(\varphi) \propto \sum_{m=1}^{\infty} \tilde{c}_m(T) \sin(m\varphi) \propto \int_0^\infty d\omega \left[\frac{a - \sqrt{a^2 - b^2}}{b} \sin\varphi + \frac{b^2 - 2a^2 + 2a\sqrt{a^2 - b^2}}{b^2} \sin(2\varphi) + \ldots \right] \tag{S68}$$

with $a = 1 + \frac{T}{2}(g_1 g_2 - 1)$ and $b = \frac{T}{2} f_1 f_2$. Explicit expressions for the Fourier coefficients can be found in the low transparency limit $T \to 0$:

$$\tilde{c}_1 \simeq \frac{T}{4} \int_0^\infty d\omega\, f_1 f_2 = \frac{T}{2} \frac{\Delta_1 \Delta_2}{\Delta_1 + \Delta_2} K\left(\frac{|\Delta_1 - \Delta_2|}{\Delta_1 + \Delta_2} \right) , \qquad \tilde{c}_2 \simeq -\frac{T^2}{16} \int_0^\infty d\omega\, f_1^2 f_2^2 = -\frac{T^2}{16} \frac{\pi}{2} \frac{\Delta_1 \Delta_2}{\Delta_1 + \Delta_2} . \tag{S69}$$

where $K(k) = \int_0^{\pi/2} d\theta/\sqrt{1 - k^2 \sin^2\theta}$ is the complete elliptic integral of the first kind and we used Eq. (19.8.12) from [S8]. Note that $\tilde{c}_1$ correctly displays the known dependence of the tunnel junction critical current on the two gaps, see [S74, Eq. (3.2.5)]. Since $K(0) = \pi/2$, for small gap asymmetry, $\Delta_1 \approx \Delta_2$, we have $E_{J2}/E_{J1} = \tilde{c}_2/2\tilde{c}_1 \approx -T/16$, cf. Eqs. (S16a) and (S16b). In the opposite limit in which one of the two gaps goes to zero, the ratio would vanish; this shows that the gap asymmetry suppresses the impact of the higher harmonics. However, this suppression can in practice be neglected: for the realistic value $\Delta_1/\Delta_2 = 0.9$ we find $E_{J2}/E_{J1} \approx -T/16(1 - 0.0007)$, a value extremely close to that for the symmetric case. Even for $\Delta_1/\Delta_2 = 0.5$ the deviation from the symmetric value is small, $E_{J2}/E_{J1} \approx -T/16(1 - 0.029)$. Furthermore, numerical integration of the formulas for the Fourier coefficients in Eq. (S68) shows that the suppression is largest at small transparency. Hence we conclude that the effect of gap asymmetry is negligible.

II. NUMERICAL METHODS

In this section, we detail the numerical methods used to obtain the parameters of the Hamiltonian Eq. (S46) to describe the experimental data. This problem belongs to the class of *inverse eigenvalue problems* (IEPs) that have been covered in a large body of scientific literature [S31, S75–S77]. We formalize the particular IEP under consideration, i.e. the Hamiltonian parameterized IEP (HamPIEP), and outline the numerical methods that we have used to find suitable solutions. Finally, we discuss several details specific to solving the HamPIEP for transmon systems.

A. Inverse eigenvalue problem

The IEP is an instance of the famous *inverse problem* [S31], which is one of the most important problems that a scientist may face: Given some data observed in an experiment, find a model that describes the data. The model is usually characterized by a set of parameters that are themselves not observable.

In quantum physics, the model is typically a Hamiltonian, and the experimental data are frequencies measured in spectroscopy experiments. An early application of the IEP in that form was the study and the description of atomic or molecular spectra, which goes back to the year 1955 [S78–S80].

1. *LiPIEP*

One of the simplest types of IEPs is the linear parameterized IEP (LiPIEP). Here the task is to find a set of parameters $\mathbf{x} = (x_1, \ldots, x_n)$ such that an $n \times n$ matrix of the form

$$A(\mathbf{x}) = A_0 + x_1 A_1 + \cdots + x_n A_n \,, \tag{S70}$$

where $A_0, A_1, \cdots, A_n$ are fixed $n{\times}n$ matrices, has eigenvalues $\mathbf{f}(\mathbf{x}) = (\lambda_1(\mathbf{x}), \ldots, \lambda_n(\mathbf{x}))$ equal to a given set of numbers $\mathbf{f}^* = (\lambda_1^*, \ldots, \lambda_n^*)$. In theory, there exists a solution for almost all A_i that is unique (up to the $n!$ permutations of the eigenvalues) [S31]. In practice, this solution can be found by applying Newton's root-finding method to the function

$$\mathbf{f}(\mathbf{x}) - \mathbf{f}^* = \begin{pmatrix} \lambda_1(\mathbf{x}) - \lambda_1^* \\ \vdots \\ \lambda_n(\mathbf{x}) - \lambda_n^* \end{pmatrix} = 0 \,, \tag{S71}$$

and the fact that the derivative of an eigenvalue $\lambda_i(\mathbf{x})$ with respect to a parameter x_k is given by

$$J_{ik} = \left(\frac{\partial \mathbf{f}}{\partial \mathbf{x}}\right)_{ik} = \frac{\partial \lambda_i}{\partial x_k} = q_i^T A_k q_i \,, \tag{S72}$$

where q_i is the eigenvector corresponding to λ_i. This means that one can iteratively find a solution by (i) diagonalizing $A(\mathbf{x})$ for a given set of parameters $\mathbf{x}$ and (ii) computing the Jacobian J in Eq. (S72) to obtain an update $\Delta\mathbf{x}$ for the next iteration (see below).

2. *HamPIEP*

The goal of the IEP solved in this work is to find a parameterized Hamiltonian that describes certain measured transition frequencies. This problem, which we call the HamPIEP, extends the simple LiPIEP in several ways:

1. It requires the diagonalization of multiple Hamiltonians $H(\mathbf{x};\boldsymbol{\theta})$ for a fixed set of constants $\boldsymbol{\theta}$ (e.g. $\boldsymbol{\theta} = n_\mathrm{g}$ for $n_\mathrm{g} = 0$ and $n_\mathrm{g} = 1/2$).

2. The Hamiltonians $H(\mathbf{x};\boldsymbol{\theta})$ may depend non-linearly on the parameters $\mathbf{x}$ (e.g. the dependence on (a, b) in Eq. (S44) for $\mathbf{x} = (E_\mathrm{C}, E_\mathrm{J1}, \Omega, G, a, b)$)

3. The eigenvalues of $H(\mathbf{x};\boldsymbol{\theta})$ require a specific labeling procedure (e.g. the assignment of photon labels k and transmon labels j to the eigenvalues $E_{\overline{kj}}(n_\mathrm{g})$, see Methods)

4. We only want specific eigenvalue combinations $\mathbf{f}$ to match the measured data $\mathbf{f}^*$ (e.g. $f^\mathrm{model}_{\mathrm{res},j} = \sum_{n_\mathrm{g}} (E_{\overline{1j}}(n_\mathrm{g}) - E_{\overline{0j}}(n_\mathrm{g}))/4\pi$ is the average of differences between eigenvalues of two Hamiltonians, see Methods).

5. The number of parameters $\#\mathbf{x}$ and the number of eigenvalue combinations $\#\mathbf{f}$ may be much smaller than the size of the Hamiltonians (i.e. $\#\mathbf{x}, \#\mathbf{f} \ll \dim(H)$).

6. The number of parameters is smaller than or equal to the number of eigenvalue combinations (i.e. $\#\mathbf{x} \leq \#\mathbf{f}$).

To solve the HamPIEP, we need the Jacobian $J = \partial\mathbf{f}/\partial\mathbf{x}$. However, points 1–4 usually make it impossible to find a closed-form expression for J such as Eq. (S72) for the LiPIEP. Therefore, we obtain J by using automatic differentiation with TensorFlow [S81] (only for the $(\bar{d}, \sigma)$ model, which involves integrals over the distribution $\rho(T)$ in Eq. (S38), we pre-compute a dense grid of Eq. (S20) using Mathematica [S82]). This circumvents the need to approximate the gradients numerically using finite differences.

We first consider the case $\#\mathbf{x} = \#\mathbf{f}$, in which one can use a root-finding algorithm to find the solution to the HamPIEP. Here we use the globally convergent Newton root-finding method with line search and backtracking [S83] (Newton-LB). If $\#\mathbf{x} = \#\mathbf{f}$, the Jacobian $J = \partial\mathbf{f}/\partial\mathbf{x}$ is a square matrix. This matrix is usually invertible, so we can use LU-decomposition of J to compute the Newton step,

$$\Delta\mathbf{x} = -J^{-1}(\mathbf{f}(\mathbf{x}) - \mathbf{f}^*)\,, \tag{S73}$$

to iteratively update $\mathbf{x} \leftarrow \mathbf{x} + \Delta\mathbf{x}$. Note that the Newton step automatically points in a direction that decreases the squared sum of differences,

$$F(\mathbf{x}) = \frac{1}{2}(\mathbf{f}(\mathbf{x}) - \mathbf{f}^*)^2\,, \tag{S74}$$

because $\nabla F \cdot \Delta\mathbf{x} = -(\mathbf{f}(\mathbf{x}) - \mathbf{f}^*)^2 < 0$. The strategy to solve the HamPIEP is thus to follow the Newton step $\mathbf{x} \leftarrow \mathbf{x} + \Delta\mathbf{x}$ as long as F decreases, to obtain quadratic convergence near the minimum. If the Newton step does not significantly reduce F, we backtrack by performing a line search along $\Delta\mathbf{x}$ to find a step $\mu\Delta\mathbf{x}$ with $\mu \in (0, 1)$ that reduces F. For this, we evaluate the function $g(\mu) = F(\mathbf{x} + \mu\Delta\mathbf{x})$ that we successively model to cubic order in μ (see [S83, Section 9.7.1] for more information). This procedure ensures a globally convergent method [S84].

In the case where $\#\mathbf{x} < \#\mathbf{f}$ and an exact solution to the HamPIEP may not exist, we use the Broyden-Fletcher-Goldfarb-Shanno (BFGS) optimization algorithm [S85] to minimize the weighted sum of absolute differences

$$W(\mathbf{x}) = |\mathbf{w} \cdot (\mathbf{f}(\mathbf{x}) - \mathbf{f}^*)|\,, \tag{S75}$$

where $|\cdot|$ denotes the L1-norm and $\mathbf{w}$ represents the weights (see the following section for how the weights are chosen). In practice, we use the implementation of the BFGS algorithm from TensorFlow Probability [S86] running on the NVIDIA A100 GPUs of JUWELS Booster [S87]. If necessary, a suitable initial value for the BFGS algorithm is obtained from Newton-LB applied to the case in which only some elements of $\#\mathbf{f}$ are used to ensure $\#\mathbf{x} = \#\mathbf{f}$.

B. Choosing appropriate weights

For the additional physical models for Josephson harmonics considered in Fig. S4 and Table S3, the number of model parameters $\#\mathbf{x}$ is often smaller than the number of measured transition frequencies $\#\mathbf{f}$. To obtain the model parameters $\mathbf{x}$ by solving the HamPIEP, we then minimize the weighted sum of absolute differences Eq. (S75). Note that this does not apply to the results presented in the main text, where e.g. the HamPIEP for the $E_{\mathrm{J}2}$ model was solved unambiguously (see Methods). However, for the additional models, the explicit form of the objective function $W(\mathbf{x})$ in Eq. (S75) is given by

$$W(\mathbf{x}) = \sum_{j=1}^{N_{\mathrm{tr}}} w_{0j} |f_{0j}^{\mathrm{model}} - f_{0j}^{\mathrm{experiment}}| + \sum_{j=0}^{N_{\mathrm{res}}} w_{\mathrm{res},j} |f_{\mathrm{res},j}^{\mathrm{model}} - f_{\mathrm{res},j}^{\mathrm{experiment}}|\,, \tag{S76}$$

Table S5. Choices of weights and resulting standard model parameters for the KIT sample for four different cases. All model parameters are given in GHz and the weights w_{0j} and $w_{\mathrm{res},j}$ are unitless.

Case	E_C/h	E_J/h	Ω/h	G/h	w_{01}	w_{02}	w_{03}	w_{04}	w_{05}	$w_{\mathrm{res},0}$	$w_{\mathrm{res},1}$
(1)	0.207	23.720	7.454	0.078	1	1/2	1/3	1/4	1/5	1	1
(2)	0.197	24.852	7.454	0.078	1	1	1/30	1/40	1/50	1	1
(3)	0.211	23.319	7.454	0.077	1/50	1/50	1/50	1/50	1/50	1	1
(4)	0.227	22.496	7.364	0.289	1	1	1/50	1	1/50	1	0

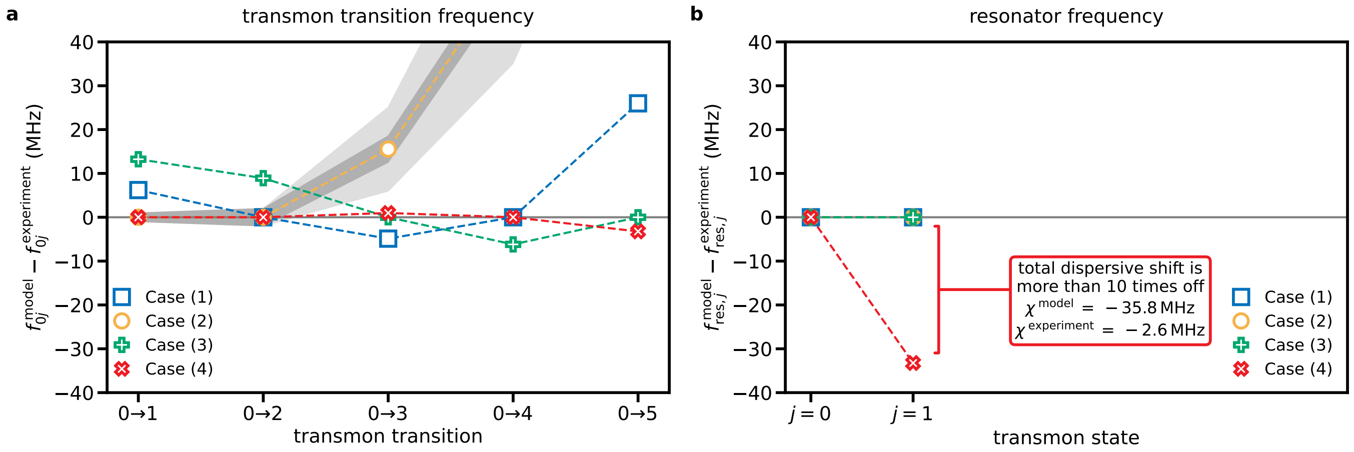

Figure S14. **Choosing appropriate weights to fit the measured transition frequencies.** **a** Results of fitting the parameters of the standard transmon model using different weights w_{0j} and $w_{\mathrm{res},j}$ (see Eq. (S76)). Markers show the difference between the model predictions f_{0j}^{model} and the measured frequencies $f_{0j}^{\mathrm{experiment}}$ for the cases (1)–(4). The corresponding weights are given in Table S5 together with the model parameters $(E_{\mathrm{C}}, E_{\mathrm{J}}, \Omega, G)$. The dark gray error band indicates the deviation for solving the HamPIEP unambiguously for the leading two transmon transition frequencies if one adds $\pm 1\,\mathrm{MHz}$ to both $f_{01}^{\mathrm{experiment}}$ and $f_{02}^{\mathrm{experiment}}/2$. The light gray error band indicates this deviation if one adds $\pm 1\,\mathrm{MHz}$ to $f_{01}^{\mathrm{experiment}}$ and $\mp 1\,\mathrm{MHz}$ to $f_{02}^{\mathrm{experiment}}/2$. **b** Difference between the predicted resonator frequencies $f_{\mathrm{res},j}^{\mathrm{model}}$ and the measured values $f_{\mathrm{res},j}^{\mathrm{experiment}}$, conditional on the transmon being in state j. The fitted models are the same as in panel a and indicated by the same colors. All models match the two resonator frequencies exactly, except in case (4) where the predicted total dispersive shift $\chi^{\mathrm{model}} = f_{\mathrm{res},1}^{\mathrm{model}} - f_{\mathrm{res},0}^{\mathrm{model}}$ differs from $\chi^{\mathrm{experiment}}$ by more than a factor of 10. Dashed lines are guides to the eye.

where w_{0j} are weights for the transmon frequencies for the transition $0 \to j$, and $w_{\mathrm{res},j}$ are weights for the resonator frequencies when the transmon is in state j. Note that fitting is a complicated procedure that always requires one to make some potentially subjective choices, i.e. choosing initial values and weights. In all cases where a certain model cannot describe the data, this will influence the final result. It is the purpose of this section to argue that the choices that we have made in this regard are justified.

When fitting the models to the measured spectra, we always put larger weights on the first two transmon frequencies and the first two resonator frequencies, to ensure that

$$f_{01}^{\mathrm{model}} \overset{!}{\approx} f_{01}^{\mathrm{experiment}} \quad \text{(dressed qubit frequency)}\,, \tag{S77a}$$

$$f_{02}^{\mathrm{model}} \overset{!}{\approx} f_{02}^{\mathrm{experiment}} \quad \text{(from dressed anharmonicity } \alpha)\,, \tag{S77b}$$

$$f_{\mathrm{res},0}^{\mathrm{model}} \overset{!}{\approx} f_{\mathrm{res},0}^{\mathrm{experiment}} \quad \text{(dressed resonator frequency)}\,, \tag{S77c}$$

$$f_{\mathrm{res},1}^{\mathrm{model}} \overset{!}{\approx} f_{\mathrm{res},1}^{\mathrm{experiment}} \quad \text{(from dispersive shift } \chi)\,, \tag{S77d}$$

where the symbol $\overset{!}{\approx}$ expresses that the fits shall try to match these values with priority. The argument for this is that the transition frequencies f_{0j}/j can be measured to roughly the same accuracy (say within $1\,\mathrm{MHz}$), so lower-index transitions like the qubit frequency f_{01} can be measured to better accuracy than the higher transition frequencies $f_{03}, f_{04}, \ldots$. Furthermore, the offset charge dispersion of higher energy levels j is much larger (cf. Fig. 4 in the main text), which additionally increases the measurement imprecision.

However, one may argue that the choice given by Eq. (S77) results in a "whiplash effect", in the sense that $\pm 1\,\mathrm{MHz}$ variations in f_{01} and $f_{02}/2$ might cause drastically different model predictions for the higher levels. In Fig. S14, we show that this is not the case (shaded gray areas). Hence, a model that is based on the standard transmon Hamiltonian Eq. (3) and that reproduces—within the experimental imprecision—the first two transition frequencies and the resonator frequencies can also not match the higher transmon transition frequencies.

Additionally, we test four different cases for choices of weights (markers) when fitting to the whole spectrum (see Table S5): Case (1) with weights $w_{0j} = 1/j$ is the most obvious choice from a theoretical point of view, since equal weights are put on f_{0j}/j which are all of similar magnitude and measurable to about $1\,\mathrm{MHz}$ accuracy. Indeed, it works very well if the model that is fitted can describe the data (see bottom panel of Fig. 3b in the main text). However, if the model being fitted cannot describe the data, such as the standard model (see the blue squares in Fig. S14),

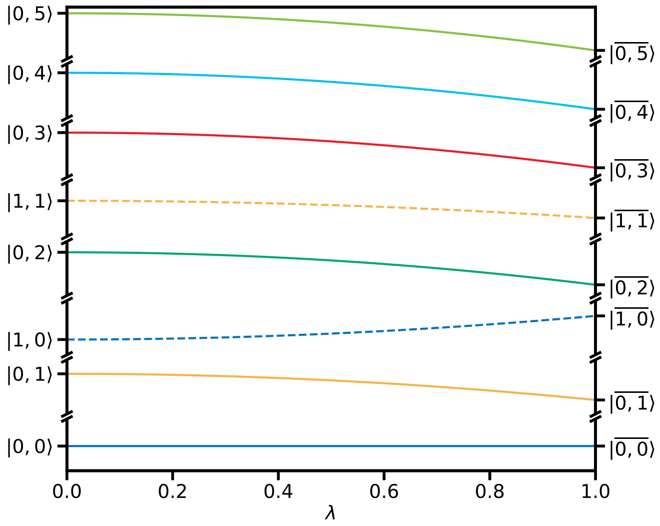

Figure S15. **Evolution of the energy spectrum from bare states $|k,j\rangle$ to dressed states $|\overline{k,j}\rangle$.** We show the evolution of the eigenenergies $E_{\bar{l}}(\lambda)$ of $H(\lambda)$ in Eq. (S78) as a function of the normalized coupling strength λ for the standard model of the KIT sample (cf. Table S3). Note that the vertical axes between the cuts do not use the same scale; the eigenenergies $E_{\bar{l}}(\lambda)$ differ by several GHz, whereas the variation between $E_{\bar{l}}(\lambda=0)$ and $E_{\bar{l}}(\lambda=1)$ is less than 25 MHz. In this part of the spectrum, there are no avoided crossings.

the fit tries to match all frequencies on average (with a hit at f_{02} and f_{05}) but the systematic error in the curvature stays. This causes the qubit frequency f_{01}, which is the most accurately measurable transmon property, to be off way beyond the experimental imprecision.

Case (2) resembles similar weights that still enforce the constraint Eq. (S77), even if the model being fitted cannot describe the data. This is shown by the yellow circles in Fig. S14.

Case (3) corresponds to equal weights on all transmon transition frequencies and larger weights on the resonator frequencies (which are measurable more accurately). However, here the fit similarly tries to match the transmon frequencies on average, at the cost of greatly overestimating the qubit frequency and the anharmonicity.

At first glance, the model corresponding to case (4) might look promising (red crosses in Fig. S14). However, the price for matching the transmon transition frequencies is a huge error in the resonator frequency $f_{\mathrm{res},1}$ when the transmon is in state $j=1$. This frequency is determined from the dispersive shift $\chi = f_{\mathrm{res},1} - f_{\mathrm{res},0}$, and is measurable to an accuracy of less than 1 MHz. The model prediction in this case, however, is off by more than 30 MHz (see Fig. S14b). Furthermore, the bare resonator frequency Ω and the coupling strength G (see Table S5) are very different for this model. Therefore, we have to reject this model as a good alternative to describe the experiment. It might be an interesting idea, though, to analyze whether alternative forms of the transmon-resonator coupling $Gn(a+a^\dagger)$ in the Hamiltonian Eq. (S46) could remedy this shortcoming.

C. Identification of dressed states

In both the standard and the harmonics model, the joint transmon-resonator Hamiltonian couples the *bare states* $|k,j\rangle$, given by the product of the transmon state $|j\rangle$ and the bare Fock state $|k\rangle$ of the resonator, through the transmon-resonator coupling $Gn(a+a^\dagger)$ (see Methods). The eigenstates of this Hamiltonian are the *dressed states* $|\overline{k,j}\rangle$, named because the hybridization of the two systems dresses each eigenenergy and eigenstate by a contribution from the other system. However, after numerical diagonalization, the eigenstates $|\bar{l}\rangle$ are typically ordered by increasing eigenenergies $E_{\bar{l}}$ for $\bar{l}=0,1,\ldots$, and it is not immediately obvious how to uniquely assign labels k and j to every eigenstate, i.e., how to identify $|\overline{k,j}\rangle \leftrightarrow |\bar{l}\rangle$, especially for large photon numbers k.

In this case, one has to make a potentially subjective choice. In the literature, one can find assignments based on iteratively constructing transmon *ladders* or *branches* using, for instance, the projection onto the bare transmon manifolds [S35] or the overlap with the state $a^\dagger\,|\overline{k-1,j}\rangle$ obtained from applying the bare raising operator of the resonator to the previously assigned state [S88, S89].

An alternative approach is to trace the evolution from bare to dressed states through the hybridization, by considering the Hamiltonian

$$H(\lambda) = \sum_j E_j\,|j\rangle\langle j| + \Omega a^\dagger a + \lambda G n(a+a^\dagger)\,, \tag{S78}$$

which interpolates between bare and dressed states using the parameter $0 \le \lambda \le 1$. For $\lambda = 0$, the assignment of labels (k,j) to the energies $E_{\bar{l}}(\lambda=0)$ is clear by definition of the bare states. Then for increasing λ, whenever two

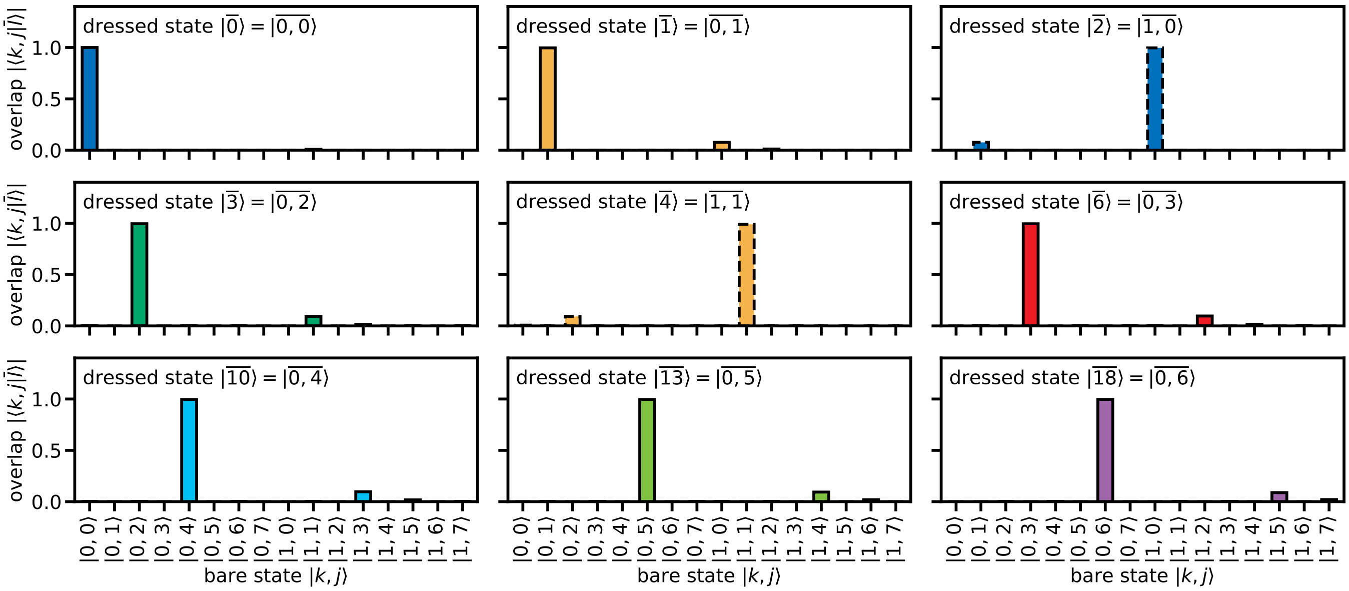

Figure S16. **Identification of dressed states within the low-energy subspace.** The overlap is given by the absolute values of the entries of the unitary change-of-basis matrix U resulting from the diagonalization. Specifically, each column $\bar{l}$ of U contains the coefficient $\langle k,j|\bar{l}\rangle$ in the row identified by k and j. Colors correspond to the respective eigenenergy curves shown in Fig. S15.

eigenenergies $E_{\bar{l}}(\lambda)$ and $E_{\bar{l}'}(\lambda)$ are about to cross, one needs to *swap* the labels (k,j) and (k',j'). After the crossing, the labels in the ordered list of eigenenergies have thus changed.

We remark that all such crossings are *avoided* crossings, since $H(\lambda)$ in Eq. (S78) is a one-parameter matrix flow, and by the Hund-von Neumann-Wigner theorem [S90, S91] (see also [S92]), the eigenvalue curves do not intersect. Still, the eigenstates hybridize (such that they form a superposition of the previous eigenstates), and by swapping the labels we can ensure that the eigenenergies leaving the crossing continue to carry the energy dependence that would cause them to be characterized as one or the other state in an experiment.

The relevant low-energy spectrum of $H(\lambda)$ in Eq. (S78) is shown in Fig. S15 as a function of λ for the KIT sample. Here the eigenenergies are still well separated and no avoided crossings occur. We can thus use the overlap of the dressed states with the bare states to assign the proper labels (k,j), as shown in Fig. S16. However, it is important to realize that this procedure does not always yield the correct assignment (as one could test by investigating higher parts of the spectra for $k>1$ or by further increasing λ).

D. Residuals for the Köln data

In contrast to the other transmon experiments considered in this work, the Köln data is special in the sense that it consists of 288 data points for the same frequency-tunable transmon sample, comprised of spectroscopy, Ramsey, and photon-number-splitting experiments. Furthermore, the data points correspond to many different qubit frequencies f_{01} tuned by an in-plane magnetic field $B_{||}$. We cover this dependence by varying only the Josephson energy E_J while keeping all other model parameters $\mathbf{x}^\mathrm{std} = (E_\mathrm{C}, \Omega, G)$ of the standard model and $\mathbf{x}^\mathrm{har} = (E_\mathrm{C}, E_\mathrm{J2}/E_\mathrm{J}, E_\mathrm{J3}/E_\mathrm{J}, E_\mathrm{J4}/E_\mathrm{J}, \Omega, G)$ of the harmonics model constant. Technically, this is done by diagonalizing the Hamiltonians for 100 linearly spaced values of E_J/h between 2 GHz and 60 GHz and using cubic interpolation on the resulting data to obtain $(f_{02}(f_{01}), f_{03}(f_{01}), \delta f_{01}(f_{01}), \delta f_{02}(f_{01}), \delta f_{03}(f_{01}), f_\mathrm{res}(f_{01}), \chi(f_{01}))$, where the dispersive shift is computed from $\chi(f_{01}) = f_{01,k=1}(f_{01}) - f_{01}$. The model parameters are obtained by fitting each of these frequencies to two measured frequencies that are closest to medians in the sets of all frequencies. We obtain $\mathbf{x}^\mathrm{std} = (h\times 0.2848\,\mathrm{GHz}, h\times 7.544\,98\,\mathrm{GHz}, h\times 0.0772\,\mathrm{GHz})$ and $\mathbf{x}^\mathrm{har} = (h\times 0.3299\,\mathrm{GHz}, -0.02298, 0.00382, -0.00128, h\times 7.545\,04\,\mathrm{GHz}, h\times 0.0832\,\mathrm{GHz})$ (see also Table S3). The residuals of this procedure are shown in Fig. S17. As expected from the results shown in the main text, the Josephson harmonics model can describe the full dataset much better than the standard model. There are a few out of the 288 data points that still show deviations in f_{02} and f_{03} (left column for $f_{01} < 5\,\mathrm{GHz}$). These data points correspond to very strong magnetic fields (the strongest $B_{||}$ is about 0.4 T). We assume that for these outliers, other higher-order, strong-field effects, not taken into account in the current

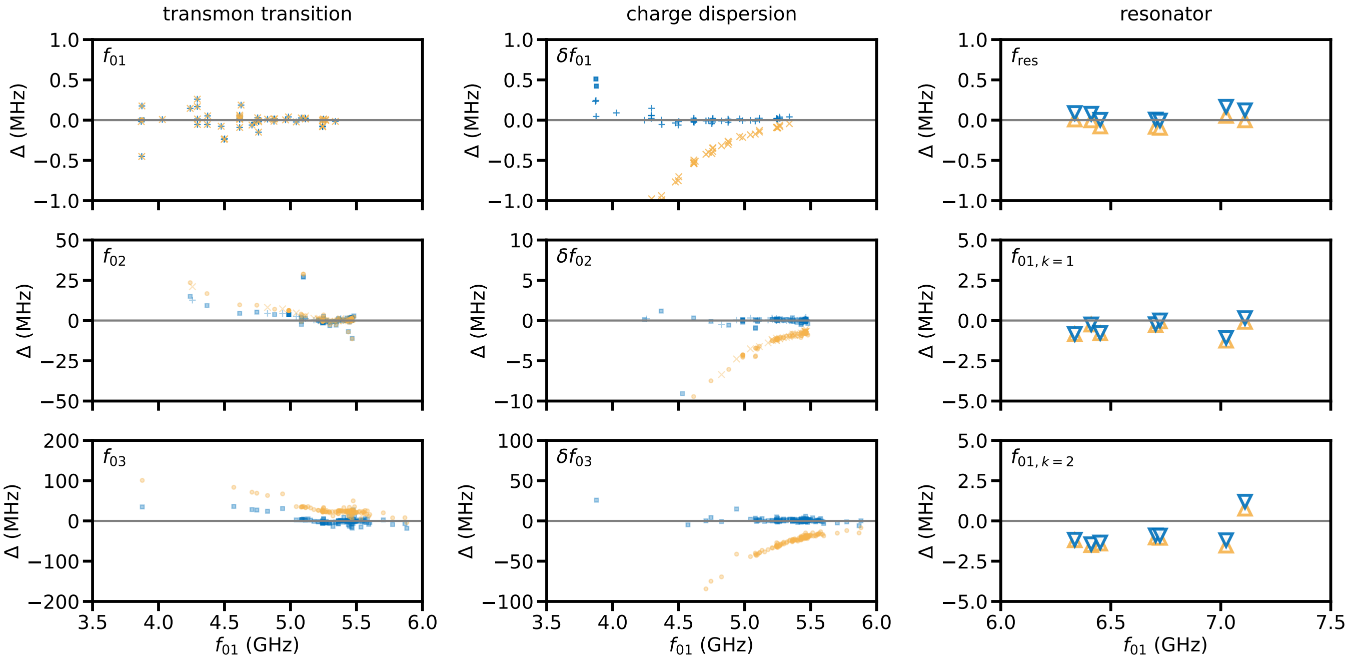

Figure S17. **Residuals for the Köln data.** All panels show the difference Δ between the frequencies obtained from the standard (yellow) or the harmonics (blue) model and the measured frequencies as a function of the transmon frequency f_{01}. The left column shows the differences in transmon transition frequencies f_{01} (top), f_{02} (middle), f_{03} (bottom). The top left panel basically represents the calibration of f_{01} depending on the in-plane magnetic field strength $B_{\|}$ (note the small scale on the vertical axis). The middle column shows the differences in the charge dispersions δf_{01} (top), δf_{02} (middle), δf_{03} (bottom). The right column represents resonator properties: the top right panel shows the difference in the (dressed) resonator frequencies, and the middle and bottom panels show the difference in the qubit frequencies $f_{01,k}$ when the resonator is not in the ground state but in the k-photon state with $k = 1$ (middle) or $k = 2$ (bottom). The frequency $f_{01,k=1}$ is shifted from f_{01} by the total dispersive shift χ (i.e. $f_{01,k=1} = f_{01} - \chi$), and $f_{01,k=2}$ is approximately shifted by 2χ from f_{01}. Data in the left and middle columns is obtained from spectroscopy (yellow squares and blue circles) and Ramsey (yellow plusses and blue crosses) experiments. Data in the right column is obtained from photon-number-splitting experiments (triangles).

models, start to play a role in this regime. However, the bulk of all data points is well described by the Josephson harmonics model.

III. SAMPLES DESCRIPTION

A. KIT

The KIT transmon qubit contains a single JJ shunted by an in-plane plate capacitor with rectangular pads, and is capacitively coupled to a lumped-element readout resonator (Fig. S18). We determine the value of the charging energy $E_\mathrm{C}/\mathrm{h} = (242 \pm 1)\,\mathrm{MHz}$ from finite-element method (FEM) simulations. The simulated geometry includes the 3D-waveguide sample holder hosting three qubit samples and their readout resonators, as shown in Fig. S18 a) together with the electric field distribution of the eigenmode associated with the central qubit. While the transmons have identical capacitor electrodes (see Fig. S18b) but different JJ overlap areas, the three readout resonators differ in the number of meanders (Fig. S18c) to ensure a frequency detuning of around 800 MHz. The samples are located on a single c-plane sapphire substrate ($t = 330\,\mu\mathrm{m}$). The dielectric permittivity of the sapphire substrate is anisotropic and defined via a tensor, where the value in parallel to the c-axis (perpendicular to the surface) is $\epsilon_{\mathrm{r},\parallel} = 11.5$ and the value perpendicular to the c-axis is $\epsilon_{\mathrm{r},\perp} = 9.3$. The shunt capacitance C_s, from which we calculate the charging energy $E_\mathrm{C} = e^2/(2C_\mathrm{s})$, and the linear stray inductance L_s arising from the leads are extracted from the simulated eigenfrequencies by varying the lumped-element inductance L_J (see Fig. S18d), and using the fit function

$$f = \frac{1}{2\pi\sqrt{(L_\mathrm{J} + L_\mathrm{s})C_\mathrm{s}}}\,. \tag{S79}$$

The obtained stray inductance is $L_\mathrm{s} \approx 380\,\mathrm{pH}$ for all samples (see Fig. S18e). In addition to the geometric contribution to the stray inductance extracted from the FEM simulations, we expect a small contribution arising from the kinetic inductance of the pure Al thin film. We can estimate this contribution from the normal-state resistivity of the Al film $\rho_\mathrm{n} = 4.3\,\mu\Omega\,\mathrm{cm}$ [S93], which we have measured for a sample fabricated in the same evaporator using the same recipe. For a film thickness $t = 70\,\mathrm{nm}$, we arrive at a kinetic sheet inductance $L_{\mathrm{k},\square} = 0.6\,\mathrm{pH}/\square$ from which we can estimate the contribution of the kinetic inductance $L_\mathrm{k} \approx 60 - 120\,\mathrm{pH}$ to the stray inductance in the sample. Hence, we expect a total stray inductance $L_\mathrm{s,tot} = L_\mathrm{s} + L_\mathrm{k} \approx 500\,\mathrm{pH}$.

Fabrication

The sample is fabricated on c-plane, double side polished sapphire substrate. A bi-layer resist stack of 700 nm MMA EL-13 and 300 nm PMMA A4 and a 10 nm gold conduction layer is used for writing with a 50 keV e-beam writer. The structures are developed in a beaker with an isopropanol-water mixture with volume ratio 3:1 at 6 °C. Before the metal deposition in a Plassys evaporation system, the substrate is cleaned with a Kaufmann ion source in an Ar/O2 descum process and the vacuum is improved using titanium gettering. The target film thicknesses of the first and second aluminum layer are 30 nm and 40 nm, respectively, and the evaporation angles are 0° and 20°, respectively. The $(100\,\mathrm{nm})^2$ sized JJ is fabricated in Dolan-style [S94] and the insulating aluminum oxide barrier is grown under static oxidation at an oxygen pressure of 10 mbar for 2.5 min.

Cooldown 1

The sample is measured in reflection in a 3D copper waveguide sample holder similar to Refs. [S95] and [S96]. The amplitude of the reflection coefficient is shown in Fig. S19 as a function of the signal frequency f_s and signal power P_s around the resonance frequency $f_\mathrm{r} = 7.4613\,\mathrm{GHz}$ of the readout resonator. With increasing signal power, the readout resonator shifts in frequency due to the non-linearity inherited from the linear coupling to the transmon, until it becomes independent of power when the transmon is in a highly excited state [S33]. From the difference in the resonator frequency we extract the Lamb shift $\Delta\omega_\mathrm{r} = -2\pi \times 7\,\mathrm{MHz}$, which is the difference between the bare and the dressed resonator frequency. In the simple transmon Hamiltonian and using a Schrieffer-Wolff transformation, the Lamb shift is identified with the partial dispersive shift $\tilde{\chi}_{12}/2$ [S41], where $\tilde{\chi}_{ij} = g_{ij}^2/(\omega_{ij} - \omega_\mathrm{r})$, from which we estimate the coupling rate $g_{12} = 2\pi \times 151\,\mathrm{MHz}$. This value is in agreement with our FEM simulations for $g_{01} = 2\pi \times 127\,\mathrm{MHz}$ when accounting for the numerical factor arising from the transition matrix element. For the fit to our extended transmon model including the higher harmonics in the Josephson effect, we do not rely on this indirect determination of the total dispersive shift χ_{01}, which under the Schrieffer-Wolff transformation is given by $\chi_{01} = 2\tilde{\chi}_{01} - \tilde{\chi}_{12}$. Instead we use the value $\chi_{01} = -2\pi \times 2.6\,\mathrm{MHz}$ measured in CD2 from the pointer states in the IQ-plane associated to the transmon ground and first excited state (see Fig. S20a). The frequencies of the individual transitions in the transmon spectrum are extracted from two-tone spectroscopy, as shown in Fig. S19b and c, and are listed in Tab. S6 together

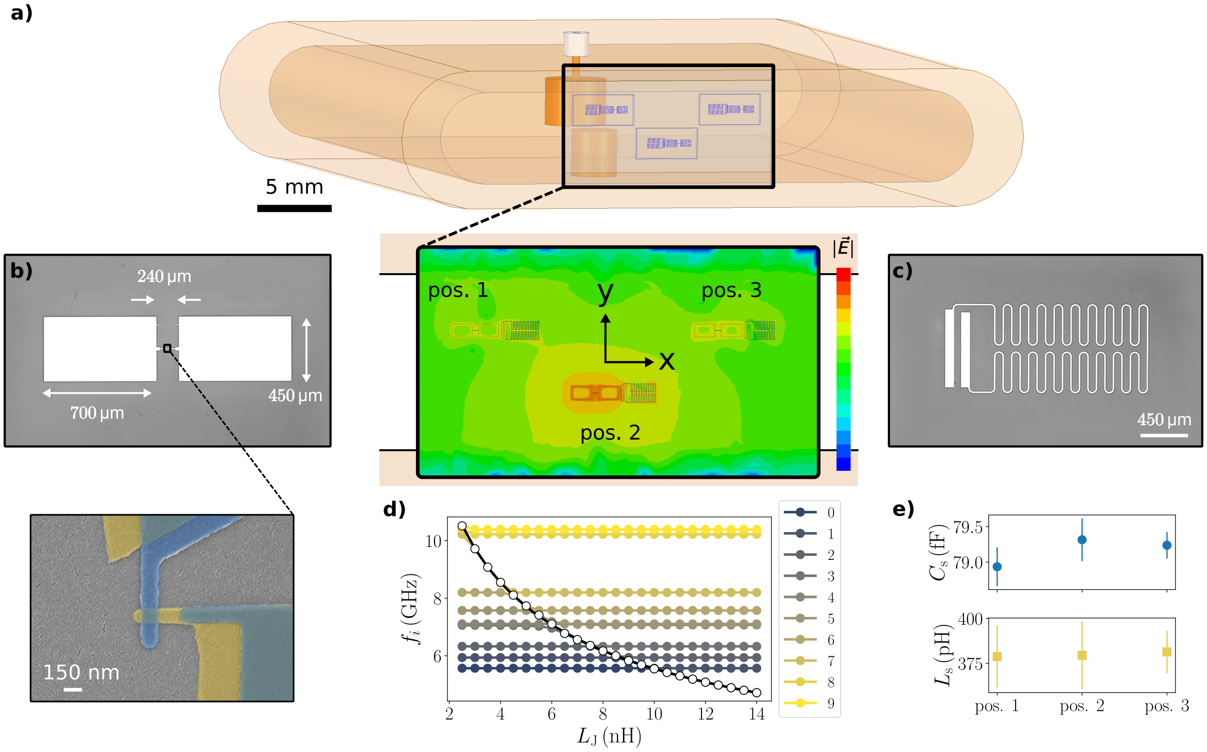

Figure S18. **Finite-element method (FEM) simulation of the KIT transmon pad geometry to obtain an estimate of the shunt capacitance and geometric stray inductance.** **a)** High-frequency structure simulator (HFSS) geometry including the copper waveguide sample holder (light orange), the input port with impedance matching (top dark orange cylinder), the tuning screw (bottom cylinder), and the sapphire substrate hosting three transmon samples. A zoom-in shows the three samples labeled according to their position on the substrate, together with the simulation result of the electric field magnitude for the transmon mode at position 2 in logarithmic scale, ranging from low (blue) to high (red) electric field strength. **b)** Optical microscope image of a transmon sample showing the aluminum thin film in white and the sapphire substrate in gray, indicating the dimensions of the electrodes. The zoom-in shows a scanning electron microscope (SEM) image of a JJ fabricated with the Dolan shadow-angle technique. The top and bottom electrodes are false-color coded in ocher and blue, respectively. **c)** Optical microscope image of the resonator geometry, with the in-plane capacitor on the left-hand side, and the meandered inductor on the right-hand side. **d)** FEM simulation results showing the frequencies of the first 10 eigenmodes above 5 GHz as a function of the lumped-element Josephson inductance L_J associated to the transmon at position 2. Error bars stemming from simulation inaccuracies on the data points are too small to be visible. The identified transmon frequencies are highlighted with white markers. The shunt capacitance $C_s \approx 80\,\mathrm{fF}$ ($E_C/h \approx 242\,\mathrm{MHz}$) and the geometric stray inductance $L_s \approx 380\,\mathrm{pH}$ ($E_L/h \approx 430\,\mathrm{GHz}$), which is an independent contribution in series with the Josepshon inductance (cf. Section I D 1), are extracted from a fit (black solid line) to the highlighted transmon frequencies using Eq. (S79). **e)** Simulation results for the shunt capacitance and stray inductance as a function of the sample position following the same procedure. The errorbars are given by the fit uncertainty.

with the readout frequencies estimated from the Lamb shift. The linewidth of the fundamental transition Fig. S19b is extracted from Lorentzian fits to the response in the reflection coefficient. As can be seen from the spectroscopic data in Fig. S19c, there are many additional features visible in the spectrum, especially at high drive powers, since we are probing the dressed energy spectrum of the transmon and the readout resonator. We argue that the frequencies associated with transitions in the transmon spectrum are independent of drive power, and only show power broadening. The transition frequencies of the multi-photon transitions indicated by the white arrows are extracted from individual measurements in the frequency vicinity of each transition, similar to Fig. S19b.

Cooldown 2

In comparison to cooldown 1, a Dimer-Josephson-Junction-Array-Amplifier (DJJAA) [S96] was added to the readout line to enable fast qubit readout and the measurement of quantum jump traces by monitoring the reflection coefficient in the vicinity of the readout resonator. At finite temperature, a histogram of such a trace reveals the pointer states

associated to different transmon states, from which we can extract the dispersive shift using their phase difference. For the calculation of the dispersive shift between the ground state and the j-th transmon state, we use

$$\chi_{0j} = -\tan\left(\frac{\varphi_0 - \varphi_j}{2}\right) \frac{1 + \left(\frac{2(\omega_\mathrm{r} - \omega_\mathrm{s})}{\kappa + \gamma}\right)^2}{2 + \frac{4(\omega_\mathrm{r} - \omega_\mathrm{s})}{\kappa+\gamma} \tan\left(\frac{\varphi_0 - \varphi_j}{2}\right)} (\kappa + \gamma)\,, \tag{S80}$$

where φ_0 and φ_j are the phase of the pointer state associated with the ground and j-th excited state, respectively, κ and γ are the external and internal decay rates of the readout resonator, ω_r is the readout resonator frequency, and ω_s is the frequency of the readout signal. From the pointer states shown in Fig. S20a, which are associated to the ground and first excited state, we extract a dispersive shift $\chi_{01} = -2\pi \times 2.6 \pm 0.1\,\mathrm{MHz}$. The internal and external decay rates of the readout resonator, $\gamma = 2\pi \times 190\,\mathrm{kHz}$ and $\kappa = 2\pi \times 3.90\,\mathrm{MHz}$, respectively, are extracted from circle fits to the complex reflection coefficient similar to Fig. S19a and Fig. S21a. The frequencies of transitions into higher excited states of the transmon spectrum are extracted from two-tone spectroscopy, exemplified for the fundamental transition in Fig. S20b, and on a larger scale in Fig. S20c.

Cooldown 3

At the end of cooldown 2, the sample was accidentally annealed during the warm-up of the measurement setup, which brought the fridge to approx. 100°C. While the readout resonator changed only insignificantly in frequency ($f_\mathrm{r} = 7.4564\,\mathrm{GHz}$), the fundamental transition of the transmon sample shifted down in frequency by more than 1 GHz. As a consequence of the increased detuning between transmon and readout, the dispersive shift decreased noticeably, resulting in a smaller Lamb shift $\Delta\omega_\mathrm{r} = -2\pi \times 2.51\,\mathrm{MHz}$ (see Fig. S21a). The extracted shift in the resonator frequency corresponds to a coupling rate $g_{12} = 2\pi \times 122\,\mathrm{MHz}$, when using the simple transmon Hamiltonian and the Schrieffer-Wolff transformation to map the transmon onto an effective two-level system. The decrease in the coupling rate compared to CD1 is not surprising, since the transition matrix elements from which the coupling rates are calculated depend on the ratio $E_\mathrm{J}/E_\mathrm{C}$ [S41], and even change for the simple transmon Hamiltonian. The decrease in the dispersive shifts enabled the discrimination of multiple pointer states in the histogram of quantum jump traces measured at the same readout frequency (see Fig. S21b), from which we extracted the multi-photon dispersive shifts χ_{0j} from the relative angle of the pointer states using Eq. (S80) (see Fig. S21c). The internal and external decay rates of the readout resonator are $\gamma = 2\pi \times 63\,\mathrm{kHz}$ and $\kappa = 2\pi \times 4.13\,\mathrm{MHz}$, respectively. We observe a weak power dependence of the extracted dispersive shifts, which is increasingly more pronounced for transitions into higher levels. We compare the measurement outcome to the prediction of the simple transmon Hamiltonian under the Schrieffer-Wolff transformation, which predicts these dispersive shifts from the partial dispersive shifts $\chi_{0j} = \tilde{\chi}_{01} + \tilde{\chi}_{j-1,j} - \tilde{\chi}_{j,j+1}$ [S41, S97]. In our calculation, we use $g_{01} = 2\pi \times 89\,\mathrm{MHz}$ for the fundamental transition, $E_\mathrm{J}/h = 13.82\,\mathrm{GHz}$ and $E_\mathrm{C}/h = 223\,\mathrm{MHz}$ (see Tab. S3). The numerical results are shown in Fig. S21c as horizontal solid black lines. While the agreement is good for the two lowest dispersive shifts, we observe increasing deviations for the higher levels $j \geq 2$.

The frequencies of transitions into higher excited states of the transmon spectrum are obtained from a combination of time-domain measurements and two-tone spectroscopy. The fundamental transition is extracted from a Ramsey-fringes measurement, which is particularly sensitive to the detuning with respect to a reference drive (see Fig. S22a). Moreover, we measure an average Ramsey coherence time of $T_2^* = (12.0 \pm 0.5)\,\mathrm{\mu s}$ (see Fig. S22b) and an energy relaxation time of $T_1 = (8.7 \pm 0.3)\,\mathrm{\mu s}$ (see Fig. S22c). The other transitions are obtained from two-tone spectroscopy (see Fig. S22d).

B. ENS

The ENS 3D transmon sample is the same as documented in refs. [S33, S98]. The transmon contains a single JJ shunted by an in-plane plate capacitor with rectangular pads, which are capacitively coupled to a copper cavity. The cavity is read out in transmission. It was fabricated in a single double-shadow evaporation step on a sapphire substrate. A MMA/PMMA resist stack was used. Before the metal deposition in a Plassys evaporation system, the sample was cleaned in-situ using an Ar/O_2 descum process. The two aluminum layers were grown with a target thickness of 35 nm and 100 nm at ±35° angles. Between the two evaporations, the oxide barrier is grown under static oxidation at a pressure of 20 mbar with a 4:1 Ar:O_2 mixture for 7 min. The JJ size is 260 nm x 200 nm. The measured qubit transition and readout resonator frequencies are listed in Tab. S6. The qubit has an average energy relaxation time of $T_1 = 15\,\mathrm{\mu s}$ and Ramsey coherence time of $T_2^* = 11\,\mathrm{\mu s}$.

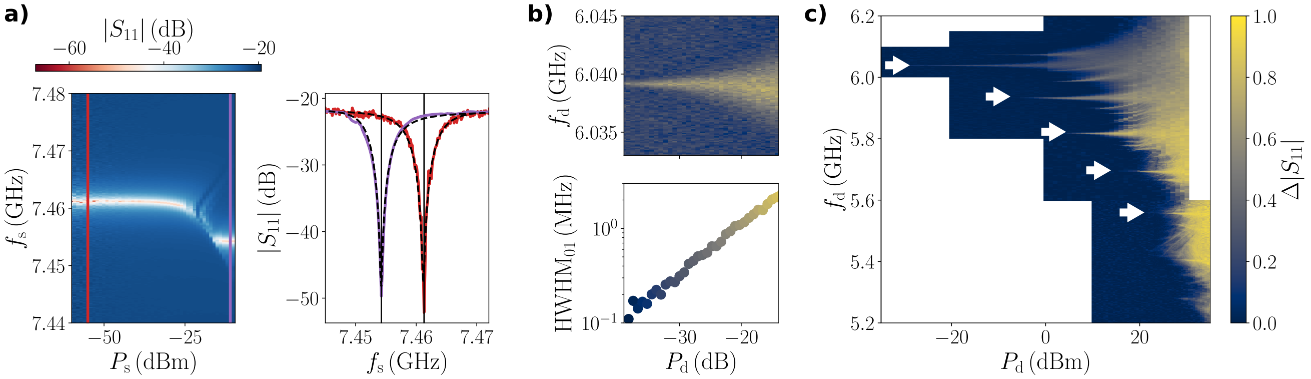

Figure S19. **Spectroscopic measurements of the KIT sample (Cooldown 1). a)** Amplitude of the reflection coefficient S_{11} in log-scale as a function of signal frequency f_s and signal power P_s at room temperature (left panel). At low readout power, the readout resonator is found at $f_r = 7.4613\,\text{GHz}$. With increasing power the resonator shifts in frequency due to the inherited non-linearity until it eventually reaches a constant frequency at $f_r = 7.4543\,\text{GHz}$ when the transmon is in a highly excited state. The right-hand panel shows two traces at low (red) and high power (violet), respectively, as indicated by the vertical lines in the 2D sweep. From the difference in transition frequency we extract the Lamb shift $\Delta\omega_r = -2\pi \times 7\,\text{MHz}$. The dashed black lines indicate the results of a circle fit to the complex reflection coefficient, from which we determine the internal and external decay rates. **b)** Two-tone spectroscopy of the fundamental transition frequency of the transmon qubit. Due to the dispersive interaction, the readout resonator shifts in frequency when the additional drive tone excites the transmon, resulting in a change in the reflected signal amplitude from low (dark) to high (yellow). With increasing drive power P_d, the transition broadens, as indicated in the bottom panel. **c)** Two-tone spectroscopy of the transmon spectrum measured by sweeping the drive frequency and drive power. The white arrows indicate the features identified as transitions in the transmon spectrum. Many other features are visible in the spectrum which are drive power dependent. The feature above the fundamental transition frequency around $f_{01} = 6.0392\,\text{GHz}$ is the transition frequency of another qubit, which is orders of magnitude more weakly coupled to the readout resonator.

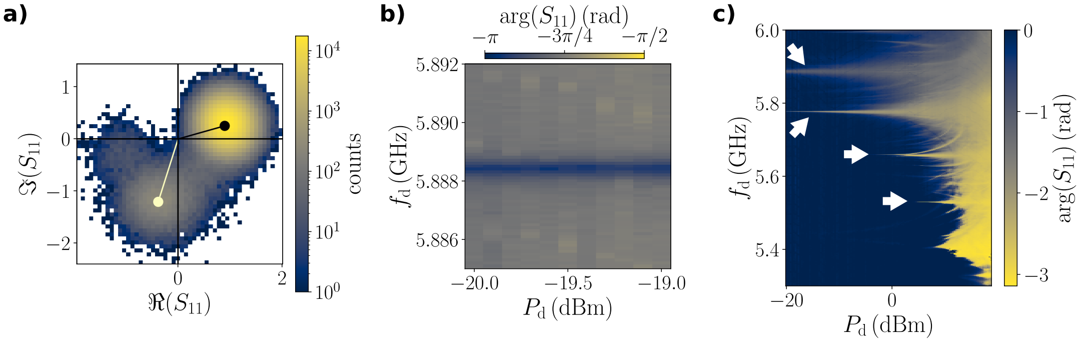

Figure S20. **Spectroscopic measurements of the KIT sample (Cooldown 2). a)** Reflection coefficient S_{11} of the readout resonator measured at the constant readout frequency $f_s = 7.4607\,\text{GHz}$ and shown in the complex plane. The two pointer states visible are associated to the ground and first excited state of the transmon. From their relative phase, we extract the dispersive shift $\chi_{01} = -2\pi \times 2.6 \pm 0.1\,\text{MHz}$. **b)** Two-tone spectroscopy around the fundamental transition $f_{01} = 5.8885\,\text{GHz}$. In comparison to the first cooldown, the frequency of the transmon changed by around 160 MHz. **c)** Transmon spectrum extracted from two-tone spectroscopy by changing the drive frequency f_d and drive power P_d. The features associated to transitions in the transmon are indicated by white arrows.

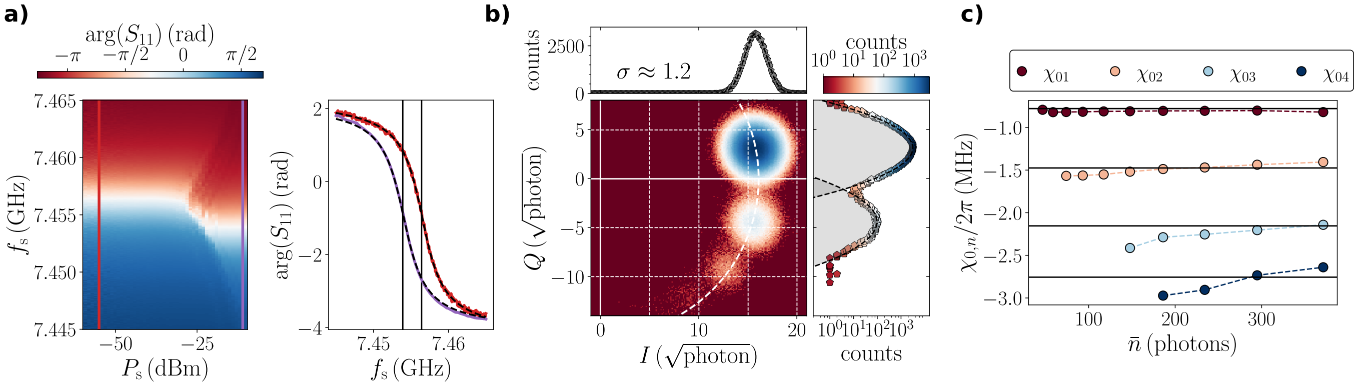

Figure S21. **Spectroscopic measurements of the KIT sample (Cooldown 3). a)** Power dependence of the reflection coefficient in the vicinity of the readout resonator, similar to Fig. S19a. At high signal power, the readout resonator effectively decouples from the transmon as it is highly excited, resulting in a frequency difference between low power ($f_\mathrm{r} = 7.4564\,\mathrm{GHz}$) and high power ($f_\mathrm{r} = 7.4539\,\mathrm{GHz}$), from which we extract the Lamb shift $\tilde{\chi}_{12}/2 = -2\pi \times 2.51\,\mathrm{MHz}$. The shift is significantly smaller compared to CD1, since the transmon-resonator detuning increased significantly between the two cooldowns. From circle fits to the complex reflection coefficient (right panel), we extract the internal and external coupling rates $\gamma = 2\pi \times 63\,\mathrm{kHz}$ and $\kappa = 2\pi \times 4.13\,\mathrm{MHz}$, respectively. **b)** Histogram of the reflection coefficient in log scale measured at the readout frequency $f_\mathrm{s} = 7.4560\,\mathrm{GHz}$. Due to the finite temperature of the transmon, multiple pointer states associated to different transmon states are visible in the complex plane spanned by the in-phase and quadrature components I and Q, respectively. As expected from a Boltzmann distribution, the occupation probability significantly decreases with the level index. The top panel shows a horizontal slice along the in-phase component I through the maximum of the ground state in linear scale, while the right-hand panel shows a slice along the quadrature component Q in log scale. The signal strength is calibrated from the measurement induced dephasing of the transmon, and expressed in units of measurement photons. From the standard deviation of the pointer states, we can calculate the measurement efficiency $\eta = 1/(2\sigma^2) = 0.35$ [S96]. **c)** Dispersive shift of the multi-photon transitions χ_{0j}, extracted from the angles between the pointer states shown in panel b) using Eq. (S80), measured as a function of the mean number of photons $\bar{n}$ in the readout resonator. For comparison reasons, the solid black lines indicate the prediction of the simple transmon model under the Schrieffer-Wolff transformation [S41, S97] using $g_{01} = 2\pi \times 89\,\mathrm{MHz}$, $E_\mathrm{J}/h = 13.82\,\mathrm{GHz}$ and $E_\mathrm{C}/h = 223\,\mathrm{MHz}$.

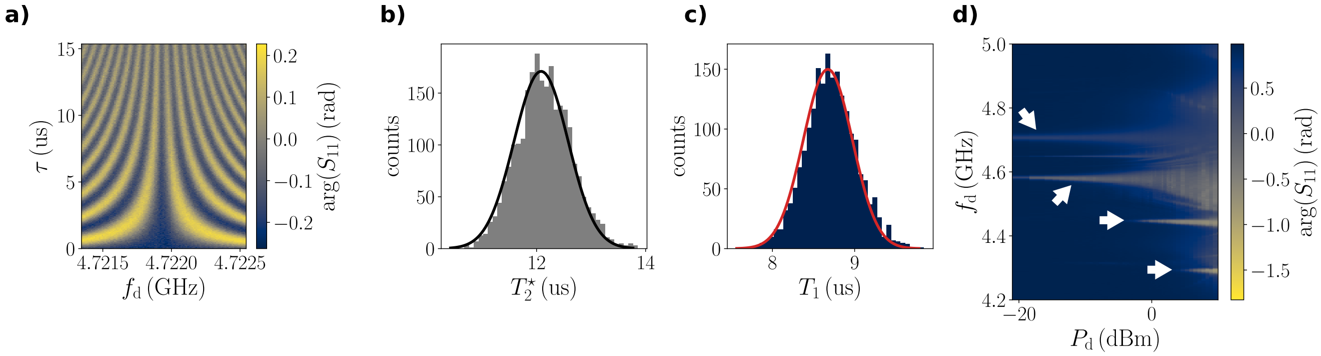

Figure S22. **Coherence and spectrum of the KIT sample (Cooldown 3). a)** Ramsey-fringes experiment with varying relative detuning between the microwave pulse to prepare the transmon in a superposition state and the fundamental transition $0 \rightarrow 1$. Since a finite detuning induces a deterministic time evolution during the idling time τ between the $\pi/2$-pulses in the Ramsey sequence, we can determine the fundamental transition frequency $f_{01} = 4.72193\,\mathrm{GHz}$ with high accuracy. **b)** By repeating the Ramsey fringes measurement, we extract the coherence time $T_2^\star = 12 \pm 0.5\,\mathrm{\mu s}$ from the statistics of the exponentially decaying envelope. **c)** The energy relaxation time of the transmon $T_1 = 8.7 \pm 0.3\,\mathrm{\mu s}$ is measured in a separate experiment. **d)** Transmon spectrum measured in a two-tone experiment by applying a drive to the transmon which induces (multi-photon) transitions into higher excited states, resulting in a shift of the resonator frequency. The white arrows indicate the transitions we can unambiguously identify from their pointer state distribution in the complex plane (cf. Fig. S21b).

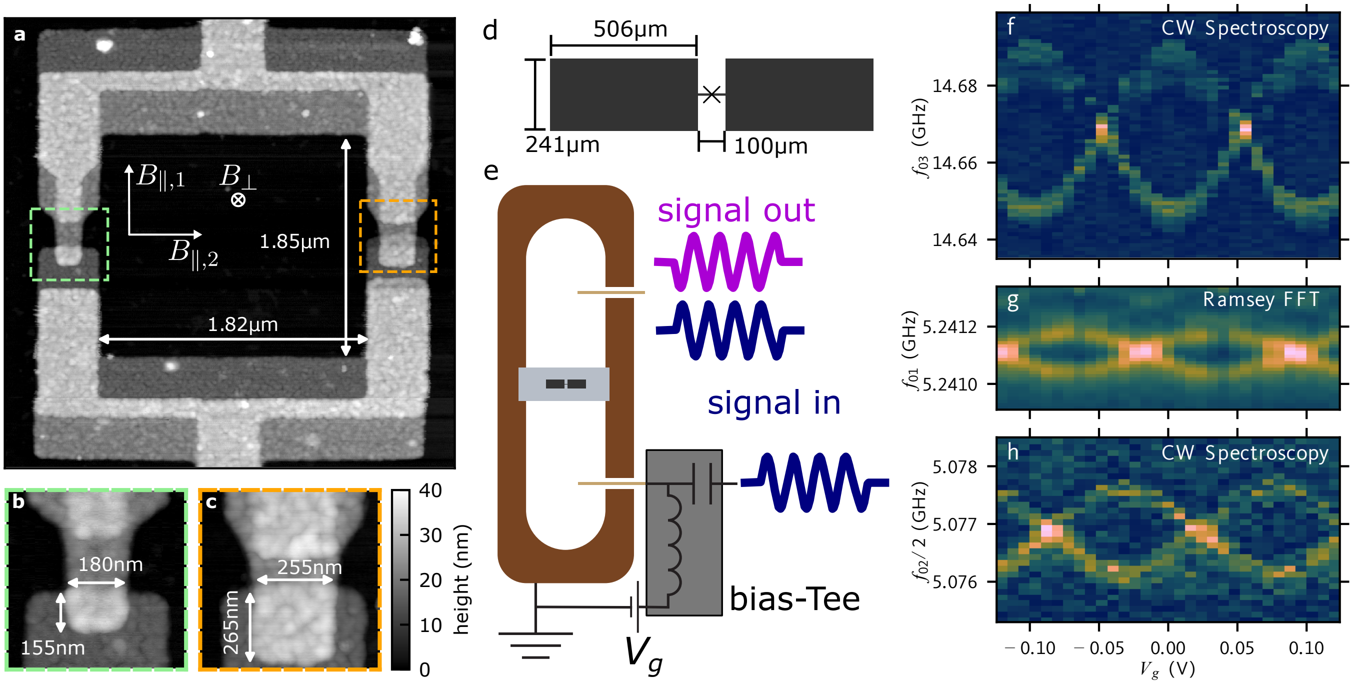

Figure S23. **Overview of the Köln device, setup and charge dispersion measurements. a** Atomic force microscope (AFM) image of the SQUID area with zoom-ins on the JJs (**b** and **c**). The magnetic field coordinate system is shown relative to the JJs and measurements of the relevant dimensions are given. **d** Transmon electrode dimensions. **e** Schematic of the transmon in a 3D cavity with two symmetrically coupled ports. A voltage V_g is applied to one of the cavity pins relative to ground in order to change the charge offset n_g of the floating transmon. **f** Two-tone spectroscopy of the f_{03} transition as a function of V_g showing the characteristic two parity branches and sinusoidal dependence. **g** Fourier transform of Ramsey datasets (excited-state population as a function of wait time) measured for the first transition f_{01} and at different V_g. **h** Two-tone spectroscopy of the $f_{02}/2$ transition. The datasets in **f**-**h** are all measured at $B_{\parallel,1} = 0.15\,\mathrm{T}$. The charge offset is subject to slow drift in time explaining the different offset in V_g for the three datasets.

C. Köln

The Köln setup and sample geometry is shown in Fig. S23a-e. The sample is a SQUID transmon with rectangular capacitor pads, which are capacitively coupled to a copper cavity. It was fabricated with the same capacitor geometry and in the same batch as the device documented in [S99]. The cavity is measured in reflection, but two-tone signals as well as time-domain pulses at the qubit frequency are applied to the other port. The energy relaxation time of the first excited state is on the order of $T_1 = 10\,\mu\mathrm{s}$, the Ramsey coherence time on the order of $T_2^* = 3\,\mu\mathrm{s}$ and T_2^{echo} is usually similar to T_1.

Fabrication

The sample is fabricated with a single electron-beam lithography step on a sapphire substrate. The Dolan bridge [S94] mask is made using an MMA-PMMA resist stack. After developement, a weak oxygen plasma is applied to remove resist residues. The JJs are fabricated using double-shadow evaporation (angles are ±20°) in a Plassys MEB 550S system without substrate cooling or heating. The target film thicknesses of the first and second aluminum layer are 10 nm and 18 nm, respectively. The aluminum oxide barriers are grown under static oxidation at an oxygen pressure of 1 mbar for 6 min. To measure the film thicknesses and JJ geometry, we took an atomic force microscope (AFM) image of the SQUID region and close-up pictures of the JJs (Fig. S23c-e). The AFM measurements for the first and second layer thickness yield 15 nm and 21 nm respectively (including the oxide layer), while the combined thickness gives 32 nm (this should include two oxide layers). The Dolan-style JJs in the SQUID have approximate areas $(160\,\mathrm{nm})^2$ and $(260\,\mathrm{nm})^2$ and the JJ-widths are compatible with the Fraunhofer dependence of E_J that we measure. The granularity of the thin-film aluminum is clearly visible in the AFM picture and the JJ area comprises many grains.

Tuning the E_J with an in-plane magnetic field

A three-axis vector magnet is used to measure the field dependence of the transmon (cf. Ref. [S99]). It changes the transmon E_J (and harmonics) due to a combination of a geometric Fraunhofer contribution and the suppression of the superconducting gap. The SQUID can be tuned with a small out-of-plane field $B_\perp$. Based on the SQUID oscillations, we align the magnetic field in the in-plane direction using the vector magnet.

The sample is fabricated with comparatively thin aluminum films and narrow leads to the JJ, in order to make it more magnetic field resilient. Moreover, we chose a relatively small SQUID area (cf. Fig. S23a) to be less sensitive to noise from the magnet. While we estimate a critical field above 0.86 T, detailed measurements for this sample were only possible up to 0.4 T, because for higher fields the SQUID starts to be unstable with flux jumps on a timescale of minutes.

Following the approach in Ref. [S99], we measure the field dependence of E_J. The E_Js of the individual junctions can be extracted from the SQUID dependence with out-of-plane field which is measured at different in-plane fields. We estimate the Fraunhofer effect to dominate the in-plane field dependence of E_J at low fields, thus the geometric asymmetry of the two JJs comprising the SQUID matters (cf. Fig. S23b-c). While E_J of the larger JJ changes from ≈ 19.4 GHz at zero field to ≈ 12.3 GHz at 0.4 T, the small JJ changes from 6.0 GHz to 4.9 GHz. At 0.4 T, the expected change in the superconducting gap is on the order of 10 %. The data presented here is predominantly measured at the bottom sweetspot of the asymmetric SQUID, because the top sweetspot is close to the cavity at low magnetic field.

The fact that the Köln device is a SQUID transmon affects the Josephson harmonics. At the bottom sweetspot of a SQUID, even harmonics would be enhanced, while odd harmonics would be suppressed. However, likely due to the fact that we only use data close to the bottom sweetspot at different in-plane field, our simplified model (cf. Section II D) that considers only an effective E_J and fixed ratios for the harmonics describes the data well.

Measuring the charge dispersion

Note that the charge dispersion grows for the higher levels and, if it cannot be explicitly resolved, it adds to the linewidth and can become a source of systematic errors in measurements. If the offset charge cannot be explicitly controlled, it will slowly drift, particularly between measurements of different transitions [S42], which could for example lead to systematic errors in estimating the anharmonicity. For the Köln sample, the offset voltage can be explicitly controlled and for many transitions both f_{ij} and δf_{ij} are measured (see Section III C). Then the frequency f_{ij} can be extracted as the mean frequency of the two parity branches reducing systematic errors and δf_{ij} is a separately measured quantity that can be used to better fit the Hamiltonian.

The gate-voltage V_g is applied to one of the cavity pins through a bias-tee to control the offset charge n_g, such that spectroscopy as a function of frequency and n_g can be measured [S42, S100]. This voltage is applied relative to the ground of the dilution refrigerator, which is connected to the 3D cavity. The voltage bias works because the pin is closer to one of the two floating transmon islands. The transitions f_{01}, $f_{02}/2$ and f_{03} were measured in two-tone continuous-wave spectroscopy as a function of V_g to extract f_{0j} and the corresponding charge dispersion δf_{0j} (example data shown in Fig. S23f,h). Both parity branches are visible in our data, as the spectroscopy measurements are slow compared to the parity switching timescale, which we measured to be on the order of 1 ms. The f_{0j} and δf_{0j} were extracted from the 2-D plots by peak finding and fitting the two branches as combined sin-functions to the data.

In addition to the spectroscopy data, Ramsey measurements on the 0-1, 1-2 and 0-2 transitions were performed as a function of V_g, showing the characteristic beating for the two parities. Fits to the Ramsey fringe data can be used to extract f_{ij} and δf_{ij}. Alternatively, a Fourier transform of the Ramsey data for f_{01} as a function of V_g closely resembles the spectroscopy data and is plotted in Fig. S23g. The frequency resolution of the Ramsey measurements should be limited only by T_2^* and there is no power broadening, Stark shifts and Photon-number broadening or splitting to consider. We confirm that continuous-wave spectroscopy and time-domain datasets are consistent with each other. For the magnetic field setting $B = 0.2$ T used in Fig. 3 in the main text, the measured qubit transition and readout resonator frequencies are listed in Tab. S6. The full spectroscopy and charge dispersion data at different in-plane magnetic field is documented in the repository [S34] accompanying this manuscript.

D. IBM

The IBM data was measured on the Hanoi, Falcon r5.11 processor for 20 out of the 27 qubits. The transition frequencies of the IBM transmons were obtained by multi-mode spectroscopy (at a single probe frequency) enabled by Qiskit Pulse [S101, S102] to measure f_{0j}/j for $j = 1, 2, 3, 4$. Since the $j = 4$ transition frequency was often near the bandwidth limit imposed by Qiskit (± 500 MHz from the $j = 1$ transition), additional sideband modulation was

Sample		f_{0j}/j (GHz)						$f_{\mathrm{res},j}$ (GHz)						
		1	2	3	4	5	6	0	1	2	3	4	5	6
KIT	CD1	6.0391	5.934	5.819	5.6945	5.5588	–	7.4613	7.4587	–	–	–	–	–
	CD2	5.8884	5.7777	5.6596	5.5305	–	–	7.4615	7.4589	–	–	–	–	–
	CD3	4.7219	4.5966	4.4590	4.3066	–	–	7.4564	7.45561	7.45495	7.45415	–	–	–
ENS		5.3548	5.2678	5.1763	5.0792	4.9758	4.8648	7.76131	7.75608	7.75135	7.747	7.74276	7.73902	7.73385
Köln	0 T	7.1095	6.977	–	–	–	–	7.5658	7.5838	–	–	–	–	–
	0.2 T	5.079	4.912	4.722	–	–	–	–	–	–	–	–	–	–
IBM	Q0	5.0354	4.8598	4.6698	4.535	–	–	7.167	7.1636	–	–	–	–	–
	Q13	4.9632	4.791	4.599	4.4251	–	–	7.222	7.219	–	–	–	–	–

Table S6. **Spectroscopy data for the measured samples.** The qubit frequencies are listed as multi-photon transitions f_{0j}/j between ground state and state j. The resonator frequencies $f_{\mathrm{res},j}$ are given for the transmon in state j. For Köln and IBM, we only show two selected field settings and qubits, respectively. The complete set of frequencies is available in the repository [S34] accompanying this manuscript.

applied at the pulse level to probe those frequencies. The measured qubit transition and readout resonator frequencies for qubits 0 and 13 are listed in Tab. S6. Spectroscopy data for all qubits is documented in the repository [S34] accompanying this manuscript.

IV. MOLECULAR DYNAMICS SIMULATION OF JUNCTION GROWTH

All molecular dynamics (MD) calculations are based on ReaxFF force fields [S103] developed by Hong and van Duin [S104], as implemented in GULP [S105]. ReaxFF allows for both the description of oxygen molecules dissociation on the surface as well as the formation of new bonds (i.e. Al-O). The visual representation of the structures is made with xcrysden [S106] and gdis [S107].

A. System construction

We consider two distinct cases for the geometric structure of the surfaces, namely Al(100) and Al(111). Both represent opposite extremes that we average over because the orientation of the Al crystals in the sample is not known. The spacing between layers in terms of the lattice constant a in Al(100) (Al(111)) is a ($a/\sqrt{3}$). A similar approach using Al(100) and Al(111) to investigate junction properties was recently proposed in [S108].

Al(111) and Al(100) are generated with a bulk parameter of $a = 4.027$ Å in both ideal (flat) configuration as well as with several types of defects (steps or islands) as schematically represented in Fig. S24. They were allowed to interact with gaseous oxygen atoms placed on top. The initial ratio between oxygen and aluminum is approximately 1:2, i.e. the initial oxygen represents a third of the total number of atoms in the system (12x12x20=2880 Al atoms and 1500 O atoms). For each model the system was propagated at 300 K using a Verlet-type algorithm for 3 ps, with a time step of 2 fs, as illustrated in Fig. S25 for model 5 (cf. Fig. S24). During propagation, the aluminum layer incorporates oxygen atoms (Fig. S25c). Since we are only interested in the resulting solid structure, the oxygen atoms that remain unbound are removed after the Verlet propagation, as shown in Fig. S25d.

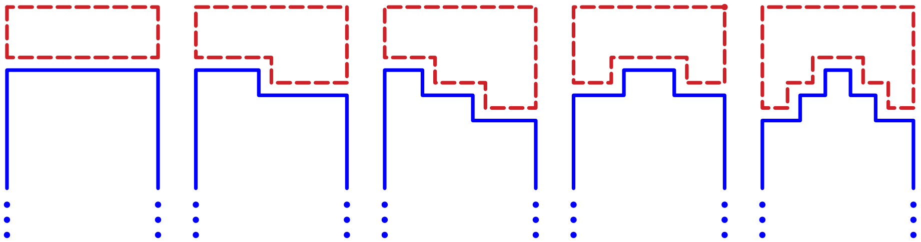

Figure S24. **Schematic representation of the five surface models considered.** We illustrate the starting conditions for the MD. The aluminum volume is schematized in blue and the oxygen volume in red. Model 1 describes an ideal smooth surface while Models 2 to 5 simulate closer to realistic geometries of rough aluminum surfaces, with step-like and respectively island-like defects.

The single layer configuration assumes periodic boundary conditions of the unit cell in the x and y directions; in this case z=infinite (2D type simulation), which means that there is no interaction between the last Al atoms from bulk and the first AlO_x layer. The multi-layer configuration used to construct Josephson junctions assumes periodic boundary conditions of the unit cell in all three directions: x, y, z. In this case z = 40 Å, which implies dynamic interaction between the last Al layer and the AlO_x layer in the neighboring unit cell. All models were further propagated for 4 ps in Verlet molecular dynamics at 300 K with a time step of 2 fs.

B. Molecular dynamics results

After performing the MD relaxation, we observe differences between Al(100) and Al(111). The data is tabulated in Table S7. On average, Al(100) accepts up to 2 % more oxygen, where the percentage refers to the total number of atoms. We also see that surfaces with defects generally accept more oxygen.

The thickness of the AlO_x layer was calculated for open surfaces and junctions in the case of all five surfaces models for both Al(100) and Al(111). The barrier thickness is defined as the difference between the coordinates of the first and the last atom that forms it. On average, the AlO_x layer formed on Al(111) is 2 Å thicker than the one formed on Al(100), as documented in table S8 and illustrated in Fig. S26. This is surprising considering that Al(111) accepts generally fewer O atoms than the Al in symmetry (100). If we compare the Al-O bond lengths, we obtain only a slightly larger length for Al(111), i.e. (1.96 ± 0.16) Å compared to (1.95 ± 0.17) Å for Al(100). Therefore the difference

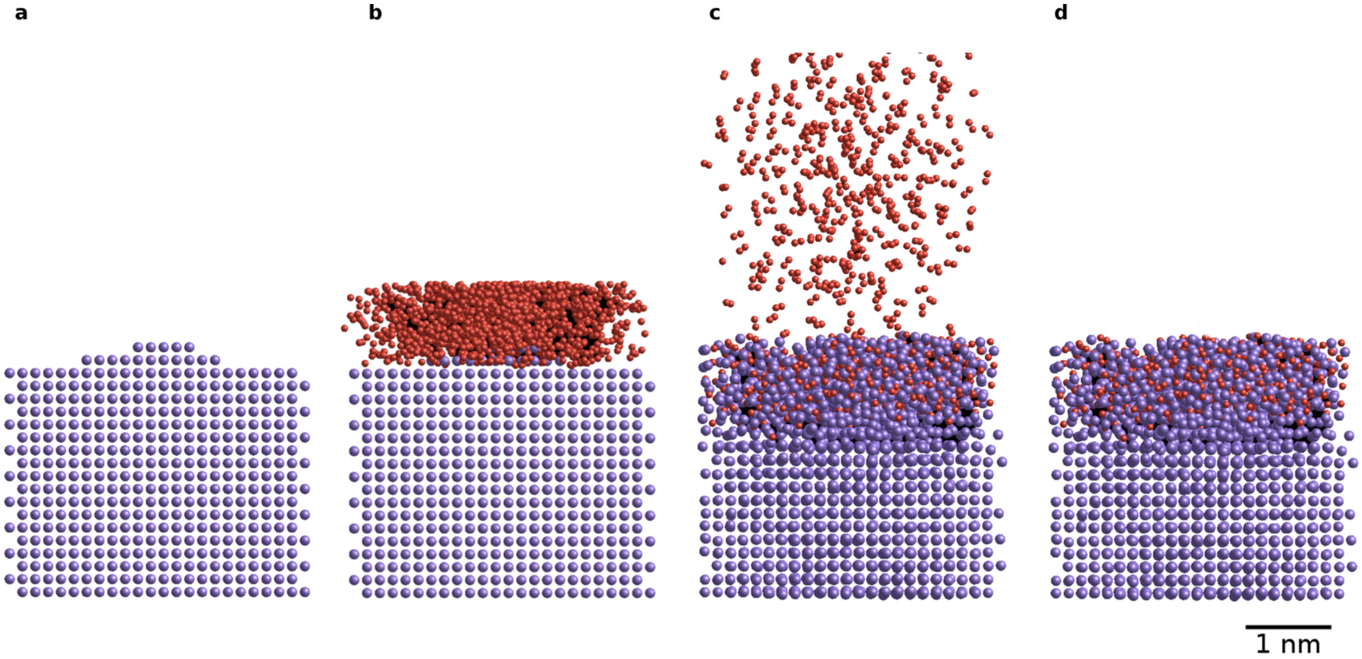

Figure S25. **The stages of creating the molecular dynamics models (aluminum in violet and oxygen in red).** The image shows an Al(100) model. **a** The initial construction of a Model 5 (double island) from Al atoms in a face-centered cubic crystal structure. **b** Oxygen atoms are randomly added to the model; the minimum distance between Al and O atoms is 1 Å and the maximum is 10 Å. **c** The system is propagated for 1500 steps of Verlet molecular dynamics at 300 K, with a time step length of 2 fs. **c** The remaining free oxygen is removed from the system, resulting in aluminum structures wrapped in a layer of AlO_x that can be further used in surface-like or junction-like configurations for the analysis of geometric parameters.

Model	Al(100)	Al(111)
Model 1	16.0	13.9
Model 2	14.3	15.0
Model 3	15.4	13.8
Model 4	15.7	14.7
Model 5	20.0	15.4
Average	16.3	14.6

Table S7. **Fraction of oxygen atoms incorporated in the oxide layer, with respect to the total number of atoms in the model**. Values are expressed in %.

in the length of the Al-O bonds formed on the two types of bulk does not explain the difference in the oxide thickness of up to 2 Å.

Model	Al(100) junction	Al(100) surface	Al(111) junction	Al(111) surface
Model 1	11.9	10.2	15.6	10.6
Model 2	13.3	8.4	16.4	10.1
Model 3	13.5	9.8	15.8	9.8
Model 4	13.8	10.1	16.9	9.9
Model 5	14.8	10.8	17.1	11.9
Average	14.5	9.9	16.4	10.5

Table S8. **AlO_x layer thickness for all models.** It is calculated as the difference between the position on the z axis of the first and last atom that forms the oxide layer. All values are expressed in Å.

However, the analysis of the structure and composition of the systems shows that different chemical bonds are formed between aluminum and oxygen in the AlO_x layer, depending on the surface orientation. In Al(111), even though it incorporates less oxygen compared to Al(100), a higher percentage of oxide and sub-oxide is formed, resulting in a thicker barrier. This fact, correlated with the bond lengths and the penetration depth of oxygen, indicates that the

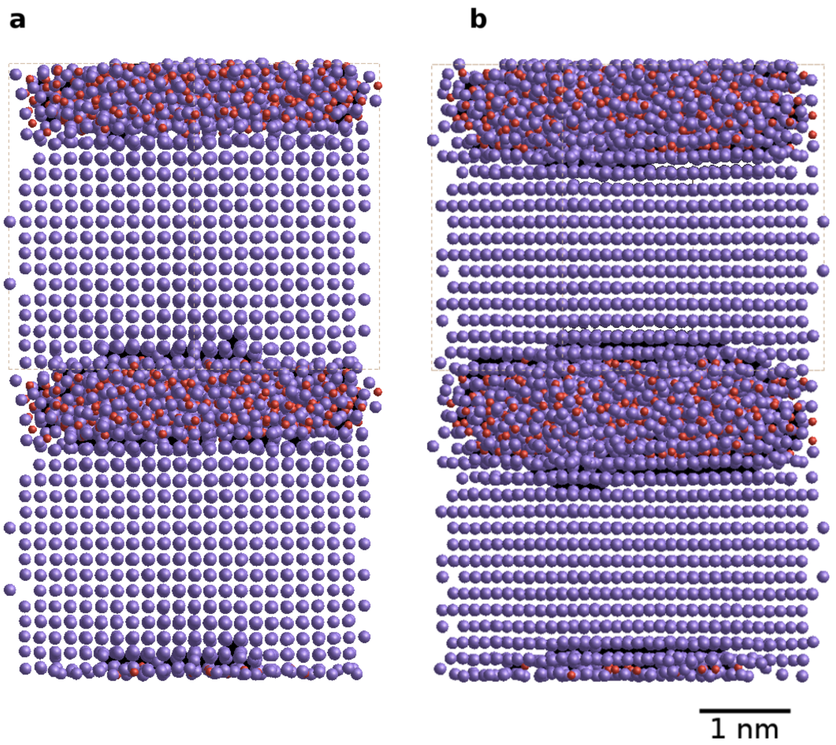

Figure S26. **Molecular model (aluminum in violet and oxygen in red) of the junction barrier thickness for different crystalline orientations.** Model 1—ideal smooth surface—in multi-layer (junction) for (a) Al(100) and (b) Al(111). The layer formed on the Al(111) surface is up to 2 Å thicker compared to the one formed on Al(100).

thickness of the AlO_x layer depends on the type and quality of the surface. The rougher the surface, the more oxygen it absorbs and consequently the larger the barrier thickness. Since the typical Josephson junction barrier is obtained by oxidizing a poly-crystalline film with grains much smaller than the junction and with random orientations, we expect the barrier to be inhomogeneous: conduction channels have different transparencies, corresponding to the crystalline orientation and oxide thickness at their respective positions.

C. Additional STEM images of JJ barriers

The Al-AlO_x-Al barriers resulting from MD models are in qualitative agreement with images obtained from STEM on JJ barriers fabricated by e-beam deposition of aluminum and thermal oxidation, as illustrated in Fig. S27.

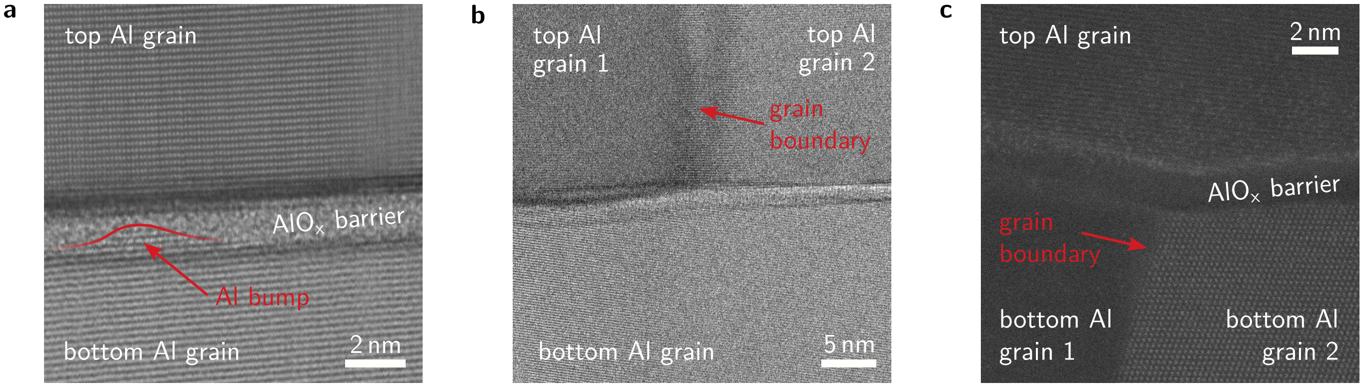

Figure S27. **Additional STEM images of Al-AlO$_x$-Al junctions.** **a** Bright field (BF) STEM image of a barrier grown under static oxidation at 1 mbar for 30 min. As the zone axes of both electrodes are pointing in different directions, the image is acquired with both axes misaligned, confirming the crystallinity of both the upper and lower electrode simultaneously. The rotational misalignment of the crystals explains the linear patterns compared to the dotted pattern in Fig. 2c in the main text. In this image, the Al grain of the bottom electrode is not homogeneously oxidized. This leads to crystalline Al reaching into the barrier region (cf. linear pattern indicated by the red arrow and line) – thus reducing the barrier thickness locally. **b** A zoomed-out BF-STEM image of a different part of the barrier in panel a showing a grain boundary in the top Al electrode, indicated by the red arrow. **c** High-angle annular dark field (HAADF) STEM image of a different region of the barrier shown in Fig. 2c in the main text. This image shows a grain boundary of the bottom Al electrode, indicated by the red arrow.

[S1] C. W. J. Beenakker, Three "universal" mesoscopic Josephson effects, in *Transport Phenomena in Mesoscopic Systems*, edited by H. Fukuyama and T. Ando (Springer Berlin Heidelberg, Berlin, Heidelberg, 1992) pp. 235–253.
[S2] Y. Asano, *Andreev Reflection in Superconducting Junctions* (Springer Singapore, 2021).
[S3] W. Haberkorn, H. Knauer, and J. Richter, A theoretical study of the current-phase relation in Josephson contacts, Phys. Status Solidi **47**, K161 (1978).
[S4] K. Böttcher and T. Kopp, Multichannel dc Josephson effect in ballistic point contacts, Phys. Rev. B **55**, 11670 (1997).
[S5] A. A. Golubov, M. Y. Kupriyanov, and E. Il'ichev, The current-phase relation in Josephson junctions, Rev. Mod. Phys. **76**, 411 (2004).
[S6] L. Glazman and G. Catelani, Bogoliubov quasiparticles in superconducting qubits, SciPost Phys. Lect. Notes , 031 (2021).
[S7] C. Kittel, *Introduction to Solid State Physics* (Wiley John + Sons, 2004) Table 1 in Chap. 6.
[S8] DLMF, *NIST Digital Library of Mathematical Functions*, http://dlmf.nist.gov/, Release 1.1.8 of 2022-12-15, F. W. J. Olver, A. B. Olde Daalhuis, D. W. Lozier, B. I. Schneider, R. F. Boisvert, C. W. Clark, B. R. Miller, B. V. Saunders, H. S. Cohl, and M. A. McClain, eds.
[S9] C. Janvier, L. Tosi, L. Bretheau, Ç. Ö. Girit, M. Stern, P. Bertet, P. Joyez, D. Vion, D. Esteve, M. F. Goffman, H. Pothier, and C. Urbina, Coherent manipulation of andreev states in superconducting atomic contacts, Science **349**, 1199 (2015).
[S10] L. J. Zeng, S. Nik, T. Greibe, P. Krantz, C. M. Wilson, P. Delsing, and E. Olsson, Direct observation of the thickness distribution of ultra thin AlOxbarriers in Al/AlOx/Al josephson junctions, J. Phys. D: Appl. Phys. **48**, 395308 (2015).
[S11] S. Fritz, L. Radtke, R. Schneider, M. Weides, and D. Gerthsen, Optimization of Al/AlOx/Al-layer systems for Josephson junctions from a microstructure point of view, J. Appl. Phys. **125**, 165301 (2019).
[S12] K. M. Schep and G. E. W. Bauer, Transport through dirty interfaces, Phys. Rev. B **56**, 15860 (1997).
[S13] G. Marchegiani, L. Amico, and G. Catelani, Quasiparticles in superconducting qubits with asymmetric junctions, PRX Quantum **3**, 040338 (2022).
[S14] I. O. Kulik and A. N. Omelyanchuk, Properties of superconducting microbridges in the pure limit, Sov. J. Low Temp. Phys. **3**, 459 (1977).
[S15] I. O. Kulik and A. N. Omelyanchuk, Contribution to the microscopic theory of the josephson effect in superconducting bridges, JETP Lett. **21**, 216 (1975).
[S16] K. K. Likharev and A. B. Zorin, Theory of the bloch-wave oscillations in small josephson junctions, J. Low Temp. Phys. **59**, 347 (1985).
[S17] A. A. Kirmani, M. Dzero, and A. Levchenko, Quasiclassical circuit theory of contiguous disordered multiband superconductors, Phys. Rev. Research **1**, 033208 (2019).
[S18] P. W. Brouwer and C. W. J. Beenakker, Anomalous temperature dependence of the supercurrent through a chaotic josephson junction, Chaos, Solitons & Fractals **8**, 1249 (1997).
[S19] R. Vijay, J. D. Sau, M. L. Cohen, and I. Siddiqi, Optimizing anharmonicity in nanoscale weak link josephson junction oscillators, Phys. Rev. Lett. **103**, 087003 (2009).
[S20] R. Vijay, E. M. Levenson-Falk, D. H. Slichter, and I. Siddiqi, Approaching ideal weak link behavior with three dimensional aluminum nanobridges, Appl. Phys. Lett. **96**, 223112 (2010).
[S21] E. M. Levenson-Falk, R. Vijay, and I. Siddiqi, Nonlinear microwave response of aluminum weak-link Josephson oscillators, Appl. Phys. Lett. **98**, 123115 (2011).
[S22] M. Liu and C. T. Black, Performance analysis of superconductor-constriction-superconductor transmon qubits, arXiv:2301.04276 [cond-mat.supr-con] (2023).
[S23] J. O'Connell Yuan, K. S. Wickramasinghe, W. M. Strickland, M. C. Dartiailh, K. Sardashti, M. Hatefipour, and J. Shabani, Epitaxial superconductor-semiconductor two-dimensional systems for superconducting quantum circuits, J. Vac. Sci. Technol. A **39**, 033407 (2021).
[S24] T. E. Hartman and J. S. Chivian, Electron tunneling through thin aluminum oxide films, Phys. Rev. **134**, A1094 (1964).
[S25] T. Aref, A. Averin, S. van Dijken, A. Ferring, M. Koberidze, V. F. Maisi, H. Q. Nguyend, R. M. Nieminen, J. P. Pekola, and L. D. Yao, Characterization of aluminum oxide tunnel barriers by combining transport measurements and transmission electron microscopy imaging, J. Appl. Phys. **116**, 073702 (2014).
[S26] L. D. Landau and E. M. Lifshitz, *Quantum Mechanics: Non-Relativistic Theory* (Elsevier, 1977).
[S27] M. Koberidze, A. V. Feshchenko, M. J. Puska, R. M. Nieminen, and J. P. Pekola, Effect of interface geometry on electron tunnelling in Al/Al_2O_3/Al junctions, J. Phys. D: Appl. Phys. **49**, 165303 (2016).
[S28] K. Gundlach and G. Heldmann, Investigation of Al2O3 film-thickness by tunnel emission and capacitance measurements, Solid State Commun. **5**, 867 (1967).
[S29] K. Gundlach and J. Hölzl, Logarithmic conductivity of Al-Al2O3-Al tunneling junctions produced by plasma- and by thermal oxidation, Surf. Sci. **27**, 125 (1971).
[S30] T. Greibe, M. P. V. Stenberg, C. M. Wilson, T. Bauch, V. S. Shumeiko, and P. Delsing, Are "pinholes" the cause of excess current in superconducting tunnel junctions? a study of Andreev current in highly resistive junctions, Phys. Rev. Lett. **106**, 097001 (2011).
[S31] M. Chu and G. Golub, *Inverse Eigenvalue Problems: Theory, Algorithms, and Applications* (Oxford University Press, 2005).

[S32] E. T. Jaynes and G. L. Bretthorst, *Probability Theory: The Logic of Science* (Cambridge University Press, 2003).
[S33] R. Lescanne, L. Verney, Q. Ficheux, M. H. Devoret, B. Huard, M. Mirrahimi, and Z. Leghtas, Escape of a driven quantum josephson circuit into unconfined states, Phys. Rev. Applied **11**, 014030 (2019).
[S34] D. Willsch and D. Rieger, Spectroscopy Data and Model Parameters for: Observation of Josephson Harmonics in Tunnel Junctions, DOI:10.26165/JUELICH-DATA/LGRHUH (2023).
[S35] M. J. Peterer, S. J. Bader, X. Jin, F. Yan, A. Kamal, T. J. Gudmundsen, P. J. Leek, T. P. Orlando, W. D. Oliver, and S. Gustavsson, Coherence and decay of higher energy levels of a superconducting transmon qubit, Phys. Rev. Lett. **114**, 010501 (2015).
[S36] A. Schneider, J. Braumüller, L. Guo, P. Stehle, H. Rotzinger, M. Marthaler, A. V. Ustinov, and M. Weides, Local sensing with the multilevel ac stark effect, Phys. Rev. A **97**, 062334 (2018).
[S37] A. Schneider, *Quantum Sensing Experiments with Superconducting Qubits*, Ph.D. thesis, Karlsruher Institut für Technologie (KIT) (2020).
[S38] E. Xie, F. Deppe, M. Renger, D. Repp, P. Eder, M. Fischer, J. Goetz, S. Pogorzalek, K. G. Fedorov, A. Marx, and R. Gross, Compact 3d quantum memory, Appl. Phys. Lett. **112**, 202601 (2018).
[S39] E. Xie, *Scalable 3D quantum memory*, Ph.D. thesis, Technische Universität München (2019).
[S40] A. Bargerbos, W. Uilhoorn, C.-K. Yang, P. Krogstrup, L. P. Kouwenhoven, G. de Lange, B. van Heck, and A. Kou, Observation of vanishing charge dispersion of a nearly open superconducting island, Phys. Rev. Lett. **124**, 246802 (2020).
[S41] J. Koch, T. M. Yu, J. Gambetta, A. A. Houck, D. I. Schuster, J. Majer, A. Blais, M. H. Devoret, S. M. Girvin, and R. J. Schoelkopf, Charge-insensitive qubit design derived from the Cooper pair box, Phys. Rev. A **76**, 042319 (2007).
[S42] J. A. Schreier, A. A. Houck, J. Koch, D. I. Schuster, B. R. Johnson, J. M. Chow, J. M. Gambetta, J. Majer, L. Frunzio, M. H. Devoret, S. M. Girvin, and R. J. Schoelkopf, Suppressing charge noise decoherence in superconducting charge qubits, Phys. Rev. B **77**, 180502 (2008).
[S43] M. L. Della Rocca, M. Chauvin, B. Huard, H. Pothier, D. Esteve, and C. Urbina, Measurement of the current-phase relation of superconducting atomic contacts, Phys. Rev. Lett. **99**, 127005 (2007).
[S44] M. L. Thompson, M. Castellanos-Beltran, P. F. Hopkins, P. D. Dresselhaus, and S. P. Benz, Effects of nonsinusoidal current phase relationships on single flux quantum circuits, IEEE Trans. Appl. Supercond. **33**, 1 (2023).
[S45] M. L. Thompson, *Study of the Current-Phase Relationship of Josephson Junctions with Insulating and Metallic Barriers*, Ph.D. thesis, University of Colorado (2023).
[S46] J. M. Gambetta, Control of superconducting qubits, in *Quantum Information Processing: Lecture Notes of the 44th IFF Spring School, Schriften des Forschungszentrums Jülich, Reihe Schlüsseltechnologien / Key Technologies*, Vol. 52, edited by D. P. DiVincenzo (Forschungszentrum Jülich, Germany, 2013).
[S47] N. Didier, E. A. Sete, M. P. da Silva, and C. Rigetti, Analytical modeling of parametrically modulated transmon qubits, Phys. Rev. A **97**, 022330 (2018).
[S48] A. Blais, A. L. Grimsmo, S. M. Girvin, and A. Wallraff, Circuit quantum electrodynamics, Rev. Mod. Phys. **93**, 025005 (2021).
[S49] F. Motzoi, J. M. Gambetta, P. Rebentrost, and F. K. Wilhelm, Simple pulses for elimination of leakage in weakly nonlinear qubits, Phys. Rev. Lett. **103**, 110501 (2009).
[S50] J. M. Chow, L. DiCarlo, J. M. Gambetta, F. Motzoi, L. Frunzio, S. M. Girvin, and R. J. Schoelkopf, Optimized driving of superconducting artificial atoms for improved single-qubit gates, Phys. Rev. A **82**, 040305 (2010).
[S51] J. M. Gambetta, F. Motzoi, S. T. Merkel, and F. K. Wilhelm, Analytic control methods for high-fidelity unitary operations in a weakly nonlinear oscillator, Phys. Rev. A **83**, 012308 (2011).
[S52] A. M. Bozkurt, J. Brookman, V. Fatemi, and A. R. Akhmerov, Double-fourier engineering of josephson energy-phase relationships applied to diodes, arXiv:2307.04830 [cond-mat.supr-con] (2023).
[S53] M. D. Reed, L. DiCarlo, B. R. Johnson, L. Sun, D. I. Schuster, L. Frunzio, and R. J. Schoelkopf, High-fidelity readout in circuit quantum electrodynamics using the jaynes-cummings nonlinearity, Phys. Rev. Lett. **105**, 173601 (2010).
[S54] S. E. Nigg, H. Paik, B. Vlastakis, G. Kirchmair, S. Shankar, L. Frunzio, M. H. Devoret, R. J. Schoelkopf, and S. M. Girvin, Black-box superconducting circuit quantization, Phys. Rev. Lett. **108**, 240502 (2012).
[S55] U. Vool and M. Devoret, Introduction to quantum electromagnetic circuits, Int. J. Circ. Theor. Appl. **45**, 897 (2017).
[S56] D. Kafri, C. Quintana, Y. Chen, A. Shabani, J. M. Martinis, and H. Neven, Tunable inductive coupling of superconducting qubits in the strongly nonlinear regime, Phys. Rev. A **95**, 052333 (2017).
[S57] M. H. Ansari, Superconducting qubits beyond the dispersive regime, Phys. Rev. B **100**, 024509 (2019).
[S58] M. Naghiloo, Introduction to experimental quantum measurement with superconducting qubits, arXiv:1904.09291 (2019).
[S59] R.-P. Riwar and D. P. DiVincenzo, Circuit quantization with time-dependent magnetic fields for realistic geometries, npj Quantum Inf. **8**, 36 (2022).
[S60] A. Miano, V. R. Joshi, G. Liu, W. Dai, P. D. Parakh, L. Frunzio, and M. H. Devoret, Hamiltonian extrema of an arbitrary flux-biased josephson circuit, arXiv:2302.03155 (2023).
[S61] J. Clarke and A. Braginski, *The SQUID Handbook: Applications of SQUIDs and SQUID Systems* (Wiley, 2006).
[S62] M. Rymarz and D. P. DiVincenzo, Consistent quantization of nearly singular superconducting circuits, arXiv:2208.11767 (2022).
[S63] A. Hall, Kepler's problem, The Analyst **10**, 65 (1883).
[S64] Z. K. Minev, Z. Leghtas, S. O. Mundhada, L. Christakis, I. M. Pop, and M. H. Devoret, Energy-participation quantization of Josephson circuits, npj Quantum Inf. **7**, 1 (2021).
[S65] J. Koch, V. Manucharyan, M. H. Devoret, and L. I. Glazman, Charging effects in the inductively shunted josephson junction, Phys. Rev. Lett. **103**, 217004 (2009).

[S66] W. C. Smith, A. Kou, U. Vool, I. M. Pop, L. Frunzio, R. J. Schoelkopf, and M. H. Devoret, Quantization of inductively shunted superconducting circuits, Phys. Rev. B **94**, 144507 (2016).

[S67] D. Thanh Le, J. H. Cole, and T. M. Stace, Building a bigger Hilbert space for superconducting devices, one Bloch state at a time, Phys. Rev. Research **2**, 013245 (2020).

[S68] F. Hassani, M. Peruzzo, L. N. Kapoor, A. Trioni, M. Zemlicka, and J. M. Fink, A superconducting qubit with noise-insensitive plasmon levels and decay-protected fluxon states, arXiv:2202.13917 (2022).

[S69] S. Sheldon, M. Sandberg, H. Paik, B. Abdo, J. M. Chow, M. Steffen, and J. M. Gambetta, Characterization of hidden modes in networks of superconducting qubits, Appl. Phys. Lett. **111**, 222601 (2017).

[S70] M. Göppl, A. Fragner, M. Baur, R. Bianchetti, S. Filipp, J. M. Fink, P. J. Leek, G. Puebla, L. Steffen, and A. Wallraff, Coplanar waveguide resonators for circuit quantum electrodynamics, J. Appl. Phys. **104**, 113904 (2008).

[S71] C. Berke, E. Varvelis, S. Trebst, A. Altland, and D. P. DiVincenzo, Transmon platform for quantum computing challenged by chaotic fluctuations, Nat. Commun. **13**, 2495 (2022).

[S72] S. Diamond, V. Fatemi, M. Hays, H. Nho, P. D. Kurilovich, T. Connolly, V. R. Joshi, K. Serniak, L. Frunzio, L. I. Glazman, and M. H. Devoret, Distinguishing parity-switching mechanisms in a superconducting qubit, PRX Quantum **3**, 040304 (2022).

[S73] A. V. Zaitsev, Quasiclassical equations of the theory of superconductivity for contiguous metals and the properties of constricted microcontacts, Sov. Phys. JETP **59**, 1015 (1984).

[S74] A. Barone and G. Paternò, *Physics and Applications of the Josephson Effect* (Wiley, 1982).

[S75] S. Friedland, Inverse eigenvalue problems, Linear Algebra Appl. **17**, 15 (1977).

[S76] S. Friedland, J. Nocedal, and M. L. Overton, The formulation and analysis of numerical methods for inverse eigenvalue problems, SIAM J. Numer. Anal. **24**, 634 (1987).

[S77] M. T. Chu, Inverse eigenvalue problems, SIAM Rev. **40**, 1 (1998).

[S78] A. C. Downing and A. S. Householder, *Some Inverse Characteristic Value Problems Which Arise in the Study of Simple Molecules*, Tech. Rep. CF-55-10-95 (Oak Ridge National Lab., Tenn., 1955).

[S79] S. Toman and J. Plíva, Multiplicity of solutions of the inverse secular problem, J. Mol. Spectrosc. **21**, 362 (1966).

[S80] P. J. Brussaard, P. W. M. Glaudemans, and A. Klein, *Shell-Model Applications in Nuclear Spectroscopy* (North-Holland Pub. Co., Amsterdam, New York, New York, 1977).

[S81] M. Abadi, A. Agarwal, P. Barham, E. Brevdo, Z. Chen, C. Citro, G. S. Corrado, A. Davis, J. Dean, M. Devin, S. Ghemawat, I. Goodfellow, A. Harp, G. Irving, M. Isard, Y. Jia, R. Jozefowicz, L. Kaiser, M. Kudlur, J. Levenberg, D. Mané, R. Monga, S. Moore, D. Murray, C. Olah, M. Schuster, J. Shlens, B. Steiner, I. Sutskever, K. Talwar, P. Tucker, V. Vanhoucke, V. Vasudevan, F. Viégas, O. Vinyals, P. Warden, M. Wattenberg, M. Wicke, Y. Yu, and X. Zheng, TensorFlow: A system for large-scale machine learning, in *12th {USENIX} Symposium on Operating Systems Design and Implementation ({OSDI} 16)* (2016) pp. 265–283, software available from tensorflow.org.

[S82] Wolfram Research, Inc, Mathematica, version 13.1 (2022), Champaign, IL, 2022.

[S83] W. H. Press, S. A. Teukolsky, W. T. Vetterling, and B. P. Flannery, *Numerical Recipes 3rd Edition: The Art of Scientific Computing* (Cambridge University Press, New York, USA, 2007).

[S84] J. E. Dennis and R. B. Schnabel, *Numerical Methods for Unconstrained Optimization and Nonlinear Equations* (Society for Industrial and Applied Mathematics, 1996).

[S85] J. Nocedal and S. J. Wright, *Numerical Optimization*, Springer Series in Operations Research and Financial Engineering (Springer New York, NY, 2006).

[S86] TensorFlow Developers, TensorFlow v2.8.0, TensorFlow Probability v0.15.0 (2022).

[S87] Jülich Supercomputing Centre, JUWELS Cluster and Booster: Exascale Pathfinder with Modular Supercomputing Architecture at Juelich Supercomputing Centre, J. of Large-Scale Res. Facil. **7**, A138 (2021).

[S88] R. Shillito, A. Petrescu, J. Cohen, J. Beall, M. Hauru, M. Ganahl, A. G. Lewis, G. Vidal, and A. Blais, Dynamics of transmon ionization, Phys. Rev. Appl. **18**, 034031 (2022).

[S89] J. Cohen, A. Petrescu, R. Shillito, and A. Blais, Reminiscence of classical chaos in driven transmons, PRX Quantum **4**, 020312 (2023).

[S90] F. Hund, Zur Deutung der Molekelspektren. I, Z. Physik **40**, 742 (1927).

[S91] J. von Neumann and E. P. Wigner, On the behaviour of eigenvalues in adiabatic processes, Phys. Z. **30**, 467 (1929).

[S92] F. Uhlig, Coalescing eigenvalues and crossing eigencurves of 1-parameter matrix flows, arXiv:2002.01274 (2020).

[S93] F. Friedrich, P. Winkel, K. Borisov, H. Seeger, C. Sürgers, I. M. Pop, and W. Wernsdorfer, Onset of phase diffusion in high kinetic inductance granular aluminum micro-squids, Superconductor Science and Technology **32**, 125008 (2019).

[S94] G. J. Dolan, Offset masks for lift-off photoprocessing, Appl. Phys. Lett. **31**, 337 (1977).

[S95] P. Winkel, K. Borisov, L. Grünhaupt, D. Rieger, M. Spiecker, F. Valenti, A. V. Ustinov, W. Wernsdorfer, and I. M. Pop, Implementation of a transmon qubit using superconducting granular aluminum, Phys. Rev. X **10**, 031032 (2020).

[S96] P. Winkel, I. Takmakov, D. Rieger, L. Planat, W. Hasch-Guichard, L. Grünhaupt, N. Maleeva, F. Foroughi, F. Henriques, K. Borisov, J. Ferrero, A. V. Ustinov, W. Wernsdorfer, N. Roch, and I. M. Pop, Nondegenerate parametric amplifiers based on dispersion-engineered josephson-junction arrays, Phys. Rev. Appl. **13**, 024015 (2020).

[S97] J. Braumüller, J. Cramer, S. Schlör, H. Rotzinger, L. Radtke, A. Lukashenko, P. Yang, S. T. Skacel, S. Probst, M. Marthaler, L. Guo, A. V. Ustinov, and M. Weides, Multiphoton dressing of an anharmonic superconducting many-level quantum circuit, Phys. Rev. B **91**, 054523 (2015).

[S98] Q. Ficheux, S. Jezouin, Z. Leghtas, and B. Huard, Dynamics of a qubit while simultaneously monitoring its relaxation and dephasing, Nat. Commun. **9**, 1926 (2018).

[S99] J. Krause, C. Dickel, E. Vaal, M. Vielmetter, J. Feng, R. Bounds, G. Catelani, J. M. Fink, and Y. Ando, Magnetic Field Resilience of Three-Dimensional Transmons with Thin-Film Al/AlO_x/Al Josephson Junctions Approaching 1 T, Phys. Rev. Appl. **17**, 034032 (2022).

[S100] L. Sun, L. DiCarlo, M. D. Reed, G. Catelani, L. S. Bishop, D. I. Schuster, B. R. Johnson, G. A. Yang, L. Frunzio, L. Glazman, M. H. Devoret, and R. J. Schoelkopf, Measurements of quasiparticle tunneling dynamics in a band-gap-engineered transmon qubit, Phys. Rev. Lett. **108**, 230509 (2012).

[S101] D. C. McKay, T. Alexander, L. Bello, M. J. Biercuk, L. Bishop, J. Chen, J. M. Chow, A. D. Córcoles, D. Egger, S. Filipp, J. Gomez, M. Hush, A. Javadi-Abhari, D. Moreda, P. Nation, B. Paulovicks, E. Winston, C. J. Wood, J. Wootton, and J. M. Gambetta, Qiskit Backend Specifications for OpenQASM and OpenPulse Experiments, arXiv:1809.03452 (2018).

[S102] T. Alexander, N. Kanazawa, D. J. Egger, L. Capelluto, C. J. Wood, A. Javadi-Abhari, and D. McKay, Qiskit Pulse: Programming Quantum Computers Through the Cloud with Pulses, arXiv:2004.06755 (2020).

[S103] T. P. Senftle, S. Hong, M. M. Islam, S. B. Kylasa, Y. Zheng, Y. K. Shin, C. Junkermeier, R. Engel-Herbert, M. J. Janik, H. M. Aktulga, T. Verstraelen, A. Grama, and A. C. T. van Duin, The ReaxFF reactive force-field: development, applications and future directions, npj Comput. Mater. **2**, 1 (2016).

[S104] S. Hong and A. C. T. van Duin, Molecular dynamics simulations of the oxidation of aluminum nanoparticles using the ReaxFF reactive force field, J. Phys. Chem. C **119**, 17876 (2015).

[S105] J. D. Gale and A. L. Rohl, The general utility lattice program (GULP), Mol. Simul. **29**, 291 (2003).

[S106] XCrySDen: a crystalline and molecular structure visualisation program.

[S107] A. R. Sean Fleming, GDIS: A visualization program for the display, manipulation, and analysis of isolated molecules and periodic structures.

[S108] M. J. Cyster, J. S. Smith, N. Vogt, G. Opletal, S. P. Russo, and J. H. Cole, Simulating the fabrication of aluminium oxide tunnel junctions, npj Quantum Inf. **7**, 12 (2021).
